# Supplementary material for: Defining the identity and the niches of epithelial stem cells with highly pleiotropic multilineage potency in the human thymus
Source: Dev Cell. 2023 Nov 20;58(22):2428–2446.e9. doi: 10.1016/j.devcel.2023.08.017 (PMC10957394; doi:10.1016/j.devcel.2023.08.017)
Supplement: Document S1. Figures S1–S9 and Tables S1–S3 [file mmc1.pdf]

Developmental Cell, Volume *58*

Supplemental information

Defining the identity and the niches of epithelial stem cells with highly pleiotropic multilineage potency in the human thymus

Roberta Ragazzini, Stefan Boeing, Luca Zanieri, Mary Green, Giuseppe D'Agostino, Kerol Bartolovic, Ana Agua-Doce, Maria Greco, Sara A. Watson, Antoniana Batsivari, Linda Ariza-McNaughton, Asllan Gjinovci, David Scoville, Andy Nam, Adrian C. Hayday, Dominique Bonnet, and Paola Bonfanti

Figure S1

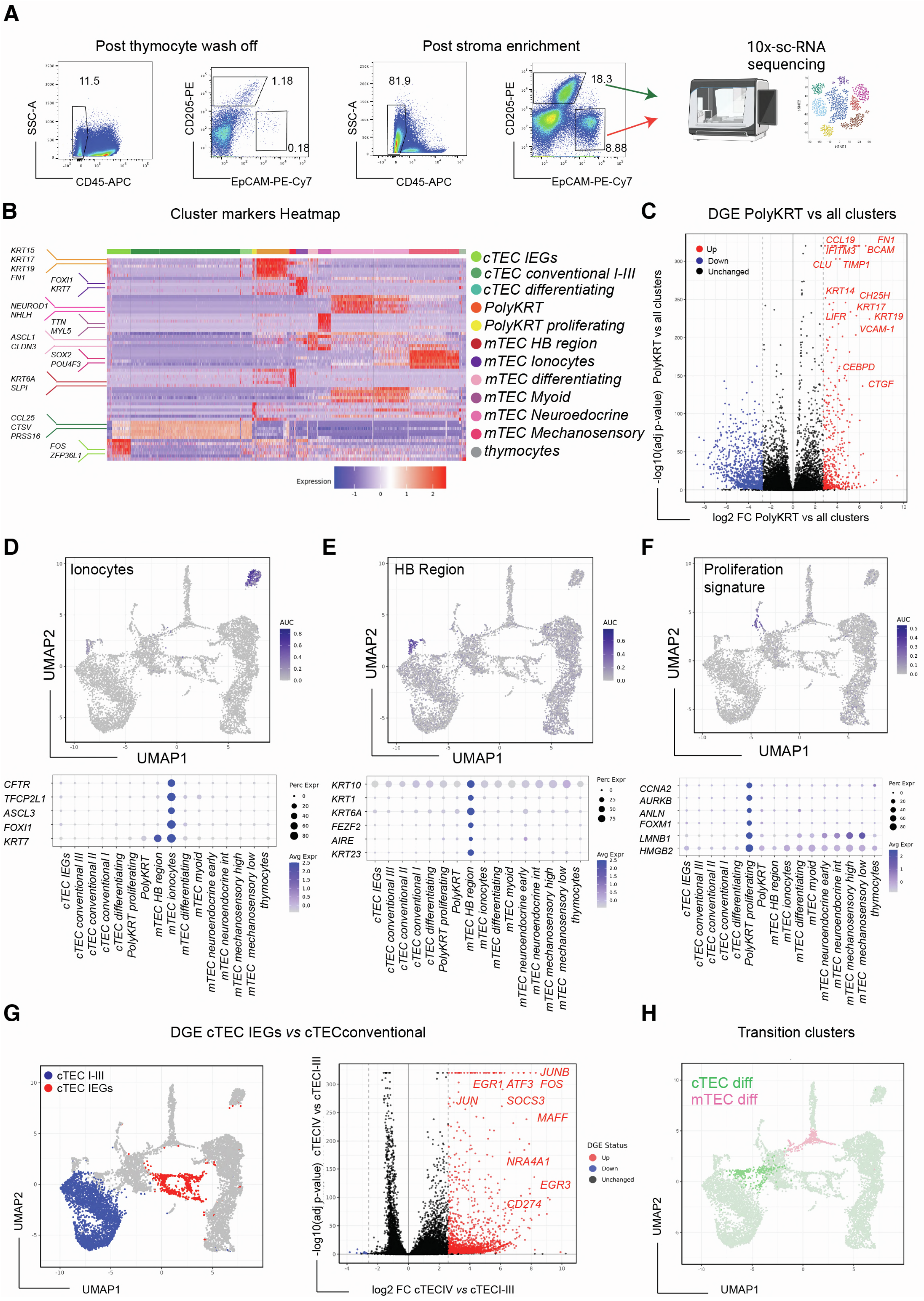

A
Post thymocyte wash off
Post stroma enrichment
10x-sc-RNA sequencing
SSC-A
CD45-APC
CD205-PE
EpCAM-PE-Cy7
11.5
1.18
0.18
81.9
18.3
8.88
B
Cluster markers Heatmap
KRT15
KRT17
KRT19
FN1
FOXI1
KRT7
NEUROD1
NHLH
TTN
MYL5
ASCL1
CLDN3
SOX2
POU4F3
KRT6A
SLPI
CCL25
CTSV
PRSS16
FOS
ZFP36L1
cTEC IEGs
cTEC conventional I-III
cTEC differentiating
PolyKRT
PolyKRT proliferating
mTEC HB region
mTEC Ionocytes
mTEC differentiating
mTEC Myoid
mTEC Neuroedocrine
mTEC Mechanosensory
thymocytes
Expression
C
DGE PolyKRT vs all clusters
Up
Down
Unchanged
-log10(adj p-value) PolyKRT vs all clusters
log2 FC PolyKRT vs all clusters
CCL19
FN1
IFITM3
BCAM
CLU
TIMP1
KRT14
CH25H
KRT17
LIFR
KRT19
VCAM-1
CEBPD
CTGF
D
Ionocytes
E
HB Region
F
Proliferation signature
UMAP1
UMAP2
AUC
CFTR
TFCP2L1
ASCL3
FOXI1
KRT7
KRT10
KRT1
KRT6A
FEZF2
AIRE
KRT23
CCNA2
AURKB
ANLN
FOXM1
LMNB1
HMGB2
Perc Expr
Avg Expr
cTEC IEGs
cTEC conventional III
cTEC conventional II
cTEC conventional I
cTEC differentiating
PolyKRT proliferating
PolyKRT
mTEC HB region
mTEC ionocytes
mTEC differentiating
mTEC myoid
mTEC neuroendocrine early
mTEC neuroendocrine int
mTEC mechanosensory high
mTEC mechanosensory low
thymocytes
G
DGE cTEC IEGs vs cTECconventional
cTEC I-III
cTEC IEGs
JUNB
EGR1
ATF3
FOS
JUN
SOCS3
MAFF
NRA4A1
EGR3
CD274
DGE Status
Up
Down
Unchanged
-log10(adj p-value) cTECIV vs cTECI-III
log2 FC cTECIV vs cTECI-III
H
Transition clusters
cTEC diff
mTEC diff

Figure S1. Human postnatal thymic epithelial cells (TECs) are highly heterogenous including a cluster with stemness traits, related to Figure 1.

(A) Representative FACS plot showing stromal CD45- (left) and cTEC and mTEC populations (right) after several washes to remove thymocytes (left panels) and after stromal enrichment (right panels). Sorted cTECs and mTECs underwent 10X-sequencing. Scheme created with *Biorender.com*.

(B) Comprehensive heatmap showing top upregulated markers for each 16 epithelial clusters identified in the UMAP; cluster names and corresponding colors are indicated on the x axes and on the right side.

(C) Volcano plot analysis of PolyKRT versus all clusters. Each dot represents one gene. A p-value of 0.05 and a fold-change of 2 are indicated by gray lines highlighting the most significantly upregulated (red) and downregulated (blue) genes.

(D-F) UMAP category feature-view and dot plots showing gene category for Ionocytes, mTEC-HB region and proliferation signature genes (*ANLN, AURKB, CCNA2, FOXM1, HMGB2, LMNB1*).

(G) Differential gene expression (DGE) analysis between cTEC-IEGs versus canonical cTEC I-III clusters (left panel). Volcano plot analysis (right panel) of cTEC-IEGs versus canonical cTEC clusters. Each dot represents one gene. A p-value of 0.05 and a fold-change of 2 are indicated by gray lines highlighting the most significantly upregulated (red) and downregulated (blue) genes.

(H) UMAP plot showing cTEC (green) and mTEC (pink) transition (differentiating) clusters.

Figure S2

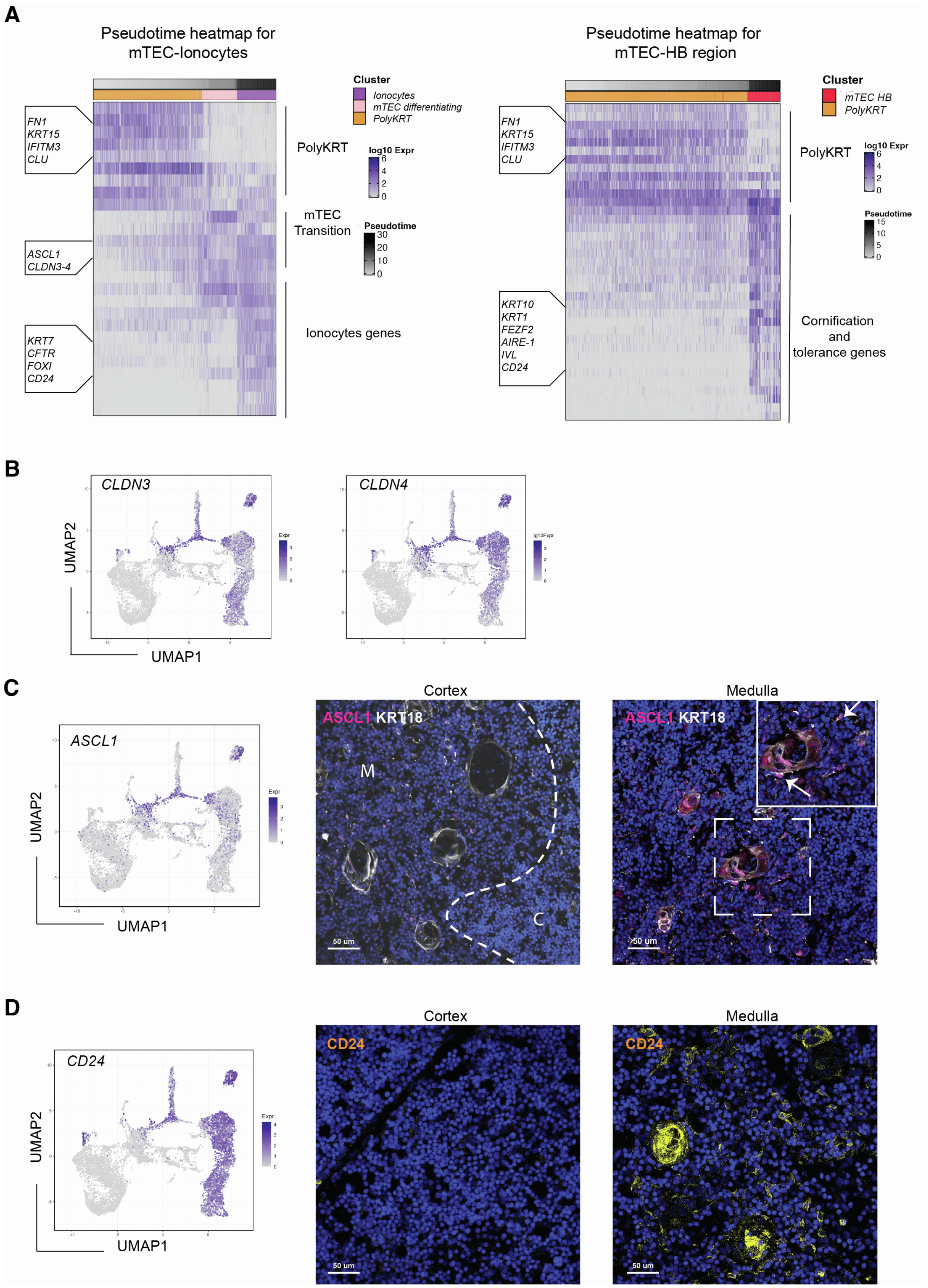
A
Pseudotime heatmap for mTEC-Ionocytes
Cluster
Ionocytes
mTEC differentiating
PolyKRT
FN1
KRT15
IFITM3
CLU
ASCL1
CLDN3-4
KRT7
CFTR
FOXI
CD24
PolyKRT
log10 Expr
mTEC Transition
Pseudotime
Ionocytes genes
Pseudotime heatmap for mTEC-HB region
Cluster
mTEC HB
PolyKRT
KRT10
KRT1
FEZF2
AIRE-1
IVL
CD24
PolyKRT
log10 Expr
Pseudotime
Cornification and tolerance genes
B
CLDN3
CLDN4
UMAP2
UMAP1
C
ASCL1
UMAP2
UMAP1
Cortex
Medulla
ASCL1 KRT18
M
C
50 um
D
CD24
UMAP2
UMAP1
Cortex
Medulla
CD24
50 um

Figure S2. Trajectory analysis reveals that specialized mTECs and cTECs differentiate from PolyKRT, related to Figure 2.

(A) Pseudotime Heatmap depicting the most time-variable genes along the single-cell trajectory from PolyKRT to mTEC-differentiating and mTEC-Ionocytes (left) and mTEC-HB region (right). Cluster colors and Pseudotime are indicated at the top of x axes.

(B) UMAP featureview plot of $\log_{10}$ expression of mTEC differentiating marker *CLDN3* (left) and *CLDN4* (right) showing transient expression from PolyKRT to medullary clusters.

(**C)** UMAP featureview plot of $\log_{10}$ expression and IHC (immunohistochemistry) of ASCL1 (magenta) in the human thymus showing co-staining with KRT18 (white). ASCL1 is scattered within medulla and is also found in proximity of HB, while not in cortex. Zoom-in inset in medullary panel highlights nuclear localisation of the transcription factor. Nuclei counterstained with DAPI. Scale bar, 50 µm (n=3, human thymi).

(**D)** UMAP featureview plot of $\log_{10}$ expression and IHC of CD24 (yellow) in medullary areas included HB and not in cortex. Nuclei counterstained with DAPI. Scale bar, 50 µm (n=3, human thymi).

Figure S3

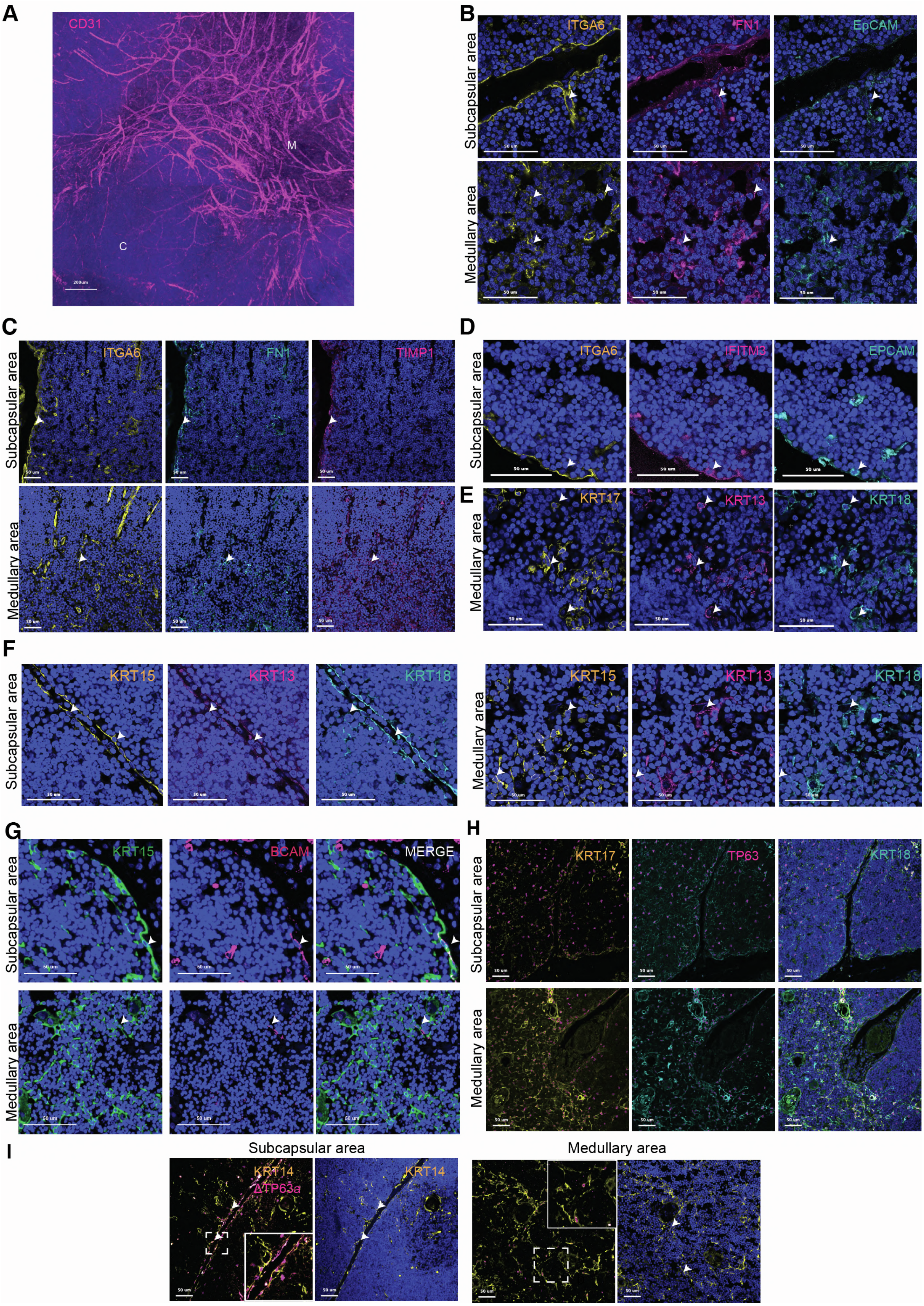
A
CD31
M
C
200um
B
ITGA6
FN1
EpCAM
Subcapsular area
Medullary area
C
ITGA6
FN1
TIMP1
Subcapsular area
Medullary area
D
ITGA6
IFITM3
EPCAM
Subcapsular area
E
KRT17
KRT13
KRT18
Medullary area
F
KRT15
KRT13
KRT18
Subcapsular area
KRT15
KRT13
KRT18
Medullary area
G
KRT15
BCAM
MERGE
Subcapsular area
Medullary area
H
KRT17
TP63
KRT18
Subcapsular area
Medullary area
I
Subcapsular area
KRT14
ΔTP63a
KRT14
Medullary area

Figure S3. PolyKRT cells reside within subcapsular and perivascular niches *in vivo,* related to Figure 3.

(A) 3D reconstruction of thick sections (300µm) of human post-natal thymus stained with endothelial marker CD31 (magenta) in cortical (C) and medullary areas (M). CD31 stains vascular structures present mostly in the medulla. Nuclei counterstained with DAPI. Scale bar, 200 µm.

(B) High magnification images showing immunofluorescence labeling of thymic epithelial cells in human thymi co-stained with anti-ITGA6 (CD49f) antibody (yellow), FN1 (magenta) and EpCAM (cyan). Top panels show co-staining in the subcapsular region; bottom panels show co-staining in medullary area. Arrows (white) highlight individual triple positive cells. Scale bars, 50 µm (n=4, human thymi).

(C) Immunofluorescence labeling of thymic epithelial stem cells in human post-natal thymus identified by co-staining with CD49f/ITGA6 (yellow), FN1 (cyan) and TIMP1 (magenta). Top panels show co-staining in the subcapsular region. Bottom panels show staining in medullary area. Arrows (white) highlight individual triple positive cells (n=3). Scale bars, 50 µm.

(D) Co-staining of CD49f (yellow), EpCAM (cyan) and IFITM3 (magenta) at high magnification in the subcapsular region. Arrows (white) highlight individual triple positive cells. Scale bars, 50 µm.

(E) Immunostaining with anti-KRT17 antibody (yellow), KRT13 (magenta) or KRT8-18 (cyan) at high magnification mark triple positive cells in medulla. Arrows (white) highlight individual cells with stem cell signature (n=4). Scale bars, 50 µm.

(F) Immunofluorescence labeling of human post-natal thymic sections by co-staining of anti-KRT15 antibody (yellow), KRT13 (magenta) or KRT8-18 (cyan) at high magnification. Left panel shows co-staining in subcapsular area and right panel in medulla. Arrows (white) highlight individual cells with triple co-staining (n=4). Scale bars, 50 µm.

(G) Immunofluorescence labeling of thymic epithelial cells co-stained with anti-KRT15 antibody (green) and BCAM (magenta) at high magnification. Top panels show co-staining in the subcapsular region; bottom panels show co-staining in medullary area. Arrows (white) highlight individual double positive cells (n=4). Scale bars, 50 µm.

(H) Immunofluorescence labelling of thymic epithelial cells with anti-KRT17 antibody (yellow), KRT18 (cyan) and TP63 (magenta), while in **(I)** KRT14 (yellow) colocalizes with deltaNTP63alpha variant (magenta) in rare cells indicated by white arrows in both subcapsular and medullary areas (n=3). All nuclei counterstained with DAPI. Scale bar, 50 µm.

Figure S4

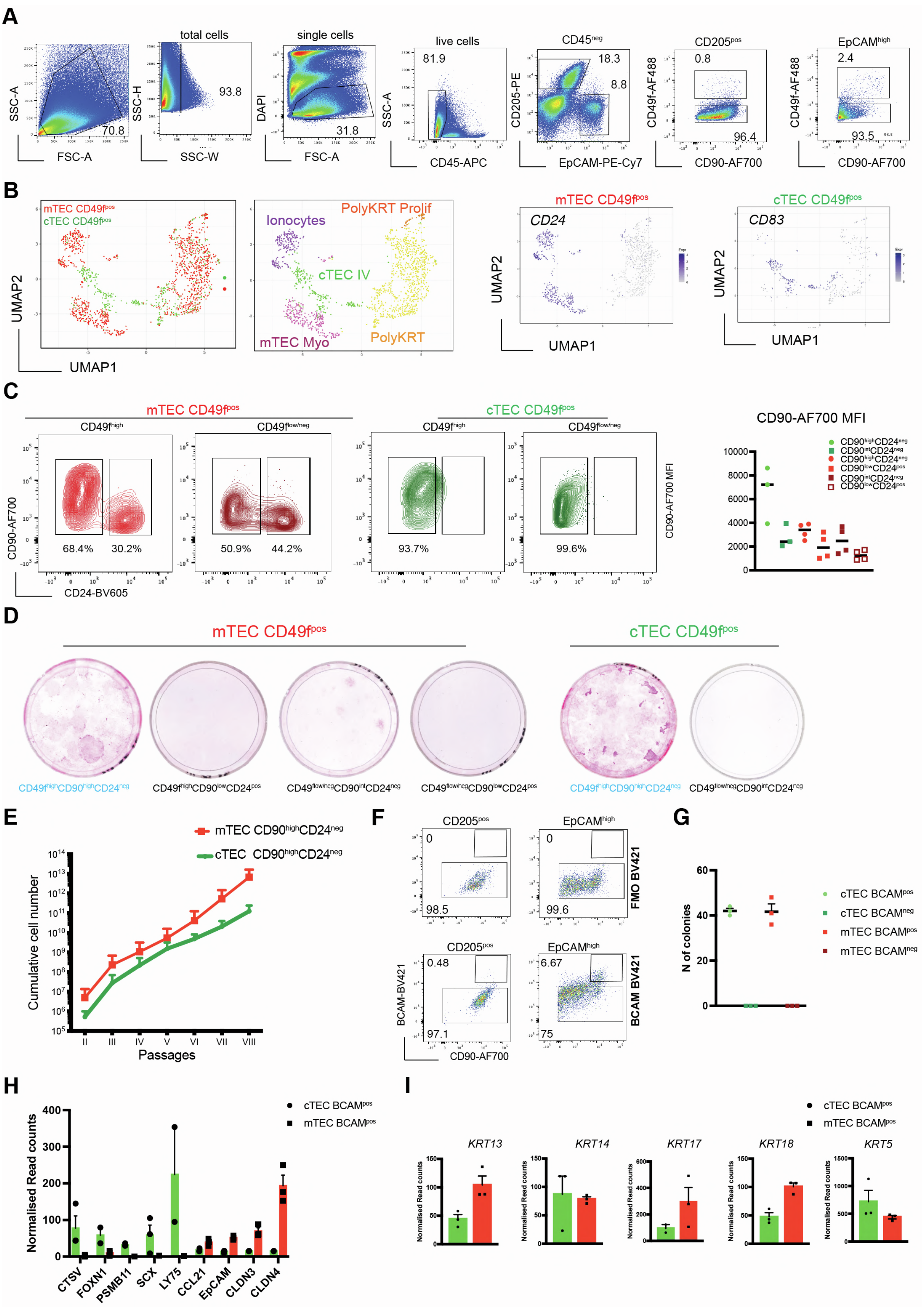

A
total cells
single cells
live cells
CD45neg
CD205pos
EpCAMhigh
SSC-A
FSC-A
SSC-H
SSC-W
DAPI
CD45-APC
CD205-PE
EpCAM-PE-Cy7
CD49f-AF488
CD90-AF700
70.8
93.8
31.8
81.9
18.3
8.8
0.8
96.4
2.4
93.5
B
mTEC CD49fpos
cTEC CD49fpos
Ionocytes
PolyKRT Prolif
cTEC IV
mTEC Myo
PolyKRT
CD24
CD83
UMAP1
UMAP2
C
mTEC CD49fpos
cTEC CD49fpos
CD49fhigh
CD49flow/neg
CD90-AF700
CD24-BV605
68.4%
30.2%
50.9%
44.2%
93.7%
99.6%
CD90-AF700 MFI
CD90highCD24neg
CD90intCD24neg
CD90highCD24neg
CD90lowCD24pos
CD90intCD24neg
CD90lowCD24pos
10000
8000
6000
4000
2000
0
D
mTEC CD49fpos
cTEC CD49fpos
CD49fhighCD90highCD24neg
CD49fhighCD90lowCD24pos
CD49flow/negCD90intCD24neg
CD49flow/negCD90lowCD24pos
CD49fhighCD90highCD24neg
CD49flow/negCD90intCD24neg
E
mTEC CD90highCD24neg
cTEC CD90highCD24neg
Cumulative cell number
Passages
II
III
IV
V
VI
VII
VIII
F
CD205pos
EpCAMhigh
0
98.5
0
99.6
FMO BV421
0.48
97.1
6.67
75
BCAM BV421
BCAM-BV421
CD90-AF700
G
N of colonies
60
40
20
0
cTEC BCAMpos
cTEC BCAMneg
mTEC BCAMpos
mTEC BCAMneg
H
cTEC BCAMpos
mTEC BCAMpos
Normalised Read counts
400
300
200
100
0
CTSV
FOXN1
PSMB11
SCX
LY75
CCL21
EpCAM
CLDN3
CLDN4
I
cTEC BCAMpos
mTEC BCAMpos
KRT13
KRT14
KRT17
KRT18
KRT5
Normalised Read counts

Figure S4. Prospective isolation of mTEC and cTEC PolyKRT, related to Figure 4.
(A) Representative FACS plots showing the gating strategy applied to separate and sort cTEC and mTEC, CD49f^{pos} (7 months old human postnatal thymus). Percentage of cells in each population is shown on the graphs.
(B) Left panels: UMAP dotplot showing scRNAseq of CD49f^{pos} mTEC (red dots) and cTEC (green dots) populations; UMAP dotplot of CD49f^{pos} mTEC and cTEC populations coloured by clusters demonstrating that PolyKRT (yellow and orange clusters) segregate with the CD49f^{pos} sorted fraction. Right panels: UMAP featureview plots of CD49f^{pos} populations show that differentiated clusters are positive for the surface marker CD24 in the medullary CD49f^{pos} (left) and for CD83 in the cortical CD49f^{pos} cells (right), while PolyKRT are negative for either CD24 or CD83.
(C) Representative FACS plots of dissociated and enriched thymic cells for N=10 human postnatal thymi (3 days to 5 years old donors). cTEC and mTEC cells were gated for CD49f^{high} expression and further subdivided and sorted according to CD24 expression. A total of four mTEC populations were isolated: CD49F^{high}CD90^{high}CD24^{neg}, CD49F^{high}CD90^{low}CD24^{pos}, CD49F$^{low/neg}$CD90^{int}CD24^{neg}, CD49F$^{low/neg}$CD90^{low}CD24^{pos}; and two cTEC populations: CD49F^{high}CD90^{high}CD24^{neg} and CD49F$^{low/neg}$CD90^{int}CD24^{neg}. On the right: Median Fluorescence Intensity (MFI) of CD90 was calculated for each of the subpopulations (n=4, human thymi).
(D) Rhodamine-B staining of each mTEC and cTEC sorted population after two passages in culture (n=4, donor-derived cultures).
(**E**) Growth curve of sorted and cultured cTEC (green) and mTEC (red) over serial passaging (x axis, number of weekly passages; y axis, cumulative cell number), mean and error plotted (n = 4, donor derived cultures).
(**F**) Representative FACS plots indicating cTEC and mTEC populations sorted for BCAM expression, (n=13, human thymi. Fluorescence Minus One Controls, FMO, included).
(**G**) Number of colonies counted for BCAM^{pos} and BCAM^{neg} expanded cells at passage II-III: 100-500 cells have been plated at day 0. (n=3, donor-derived cultures).
(H) Gene expression profiles of cortical and medullary markers expressed by BCAM^{pos} TEC fraction via nCounter NanoString Technologies (n=3, human thymi).
(I) Keratin (KRT) genes expression profile of cortical and medullary BCAM^{pos} TEC fractions via nCounter NanoString Technologies (n=3, human thymi). Differences in KRT genes expression is not significant after performing Mann-Whitney test.

Figure S5

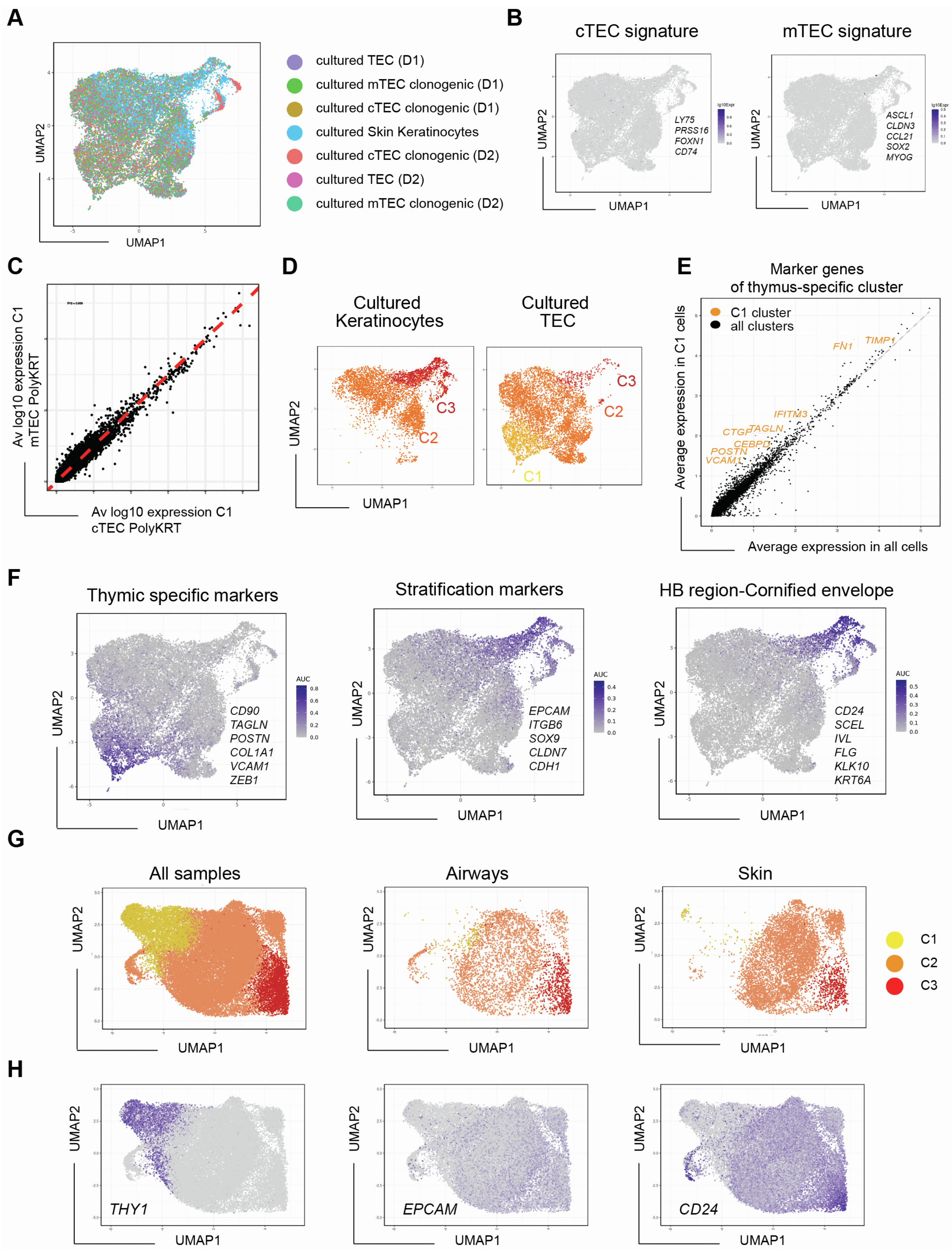
A
UMAP2
UMAP1
cultured TEC (D1)
cultured mTEC clonogenic (D1)
cultured cTEC clonogenic (D1)
cultured Skin Keratinocytes
cultured cTEC clonogenic (D2)
cultured TEC (D2)
cultured mTEC clonogenic (D2)
B
cTEC signature
mTEC signature
LY75
PRSS16
FOXN1
CD74
ASCL1
CLDN3
CCL21
SOX2
MYOG
UMAP2
UMAP1
C
Av log10 expression C1
mTEC PolyKRT
Av log10 expression C1
cTEC PolyKRT
D
Cultured
Keratinocytes
Cultured
TEC
C1
C2
C3
UMAP2
UMAP1
E
Marker genes
of thymus-specific cluster
C1 cluster
all clusters
FN1
TIMP1
IFITM3
CTGF
TAGLN
CEBPD
POSTN
VCAM1
Average expression in C1 cells
Average expression in all cells
F
Thymic specific markers
CD90
TAGLN
POSTN
COL1A1
VCAM1
ZEB1
Stratification markers
EPCAM
ITGB6
SOX9
CLDN7
CDH1
HB region-Cornified envelope
CD24
SCEL
IVL
FLG
KLK10
KRT6A
AUC
UMAP2
UMAP1
G
All samples
Airways
Skin
C1
C2
C3
UMAP2
UMAP1
H
THY1
EPCAM
CD24
UMAP2
UMAP1

Figure S5. Single-cell RNA-seq analysis defines a thymus-specific cell cluster *in vitro*, related to Figure 5.

(A) UMAP plot showing the distribution of each sequenced epithelial cell in culture by similarity. Analysis has been performed on two donor samples (D1 and D2) for each thymic culture type.

(B) UMAP featureview plot of $\log_{10}$ expression of specialized cortical (left panel) and medullary (right panel) markers, absent in all thymic cultures.

(C) Comparison of average expression levels between cluster C1 mTEC PolyKRT and C1 cTEC PolyKRT. The red dashed line is the identity line.

(D) UMAP plot visualization of skin keratinocytes and thymic cells colored by cell cluster group per each representative sample. Cluster C1 is absent in skin keratinocytes.

(E) Average expression of C1 genes is plotted against average expression in all the other clusters. Upregulated genes in cluster C1 are displayed in orange.

(F) UMAP category featureview plots showing $\log_{10}$ expression of genes belonging to C1 thymic specific cluster: *THY1/CD90, TAGLN, POSTN, COL1A1, VCAM-1, ZEB1* (left panel); genes belonging to C2 stratified cell cluster *EPCAM, ITGB6, SOX9, CLDN7, CDH1*, and C3 cornified/differentiated cluster *CD24, SCEL, IVL, FLG, KLK10, KRT6A*.

(G) UMAP plot visualization of cultured epithelial cells colored by cell cluster group per each representative sample (thymic, skin keratinocytes and airways basal cells).

(H) UMAP plot visualisation ($\log_{10}$ expression) of marker genes across clusters confirmed expression of *THY1* in C1, *EPCAM* in C2/C3 and *CD24* in C2/C3 clusters.

Figure S6

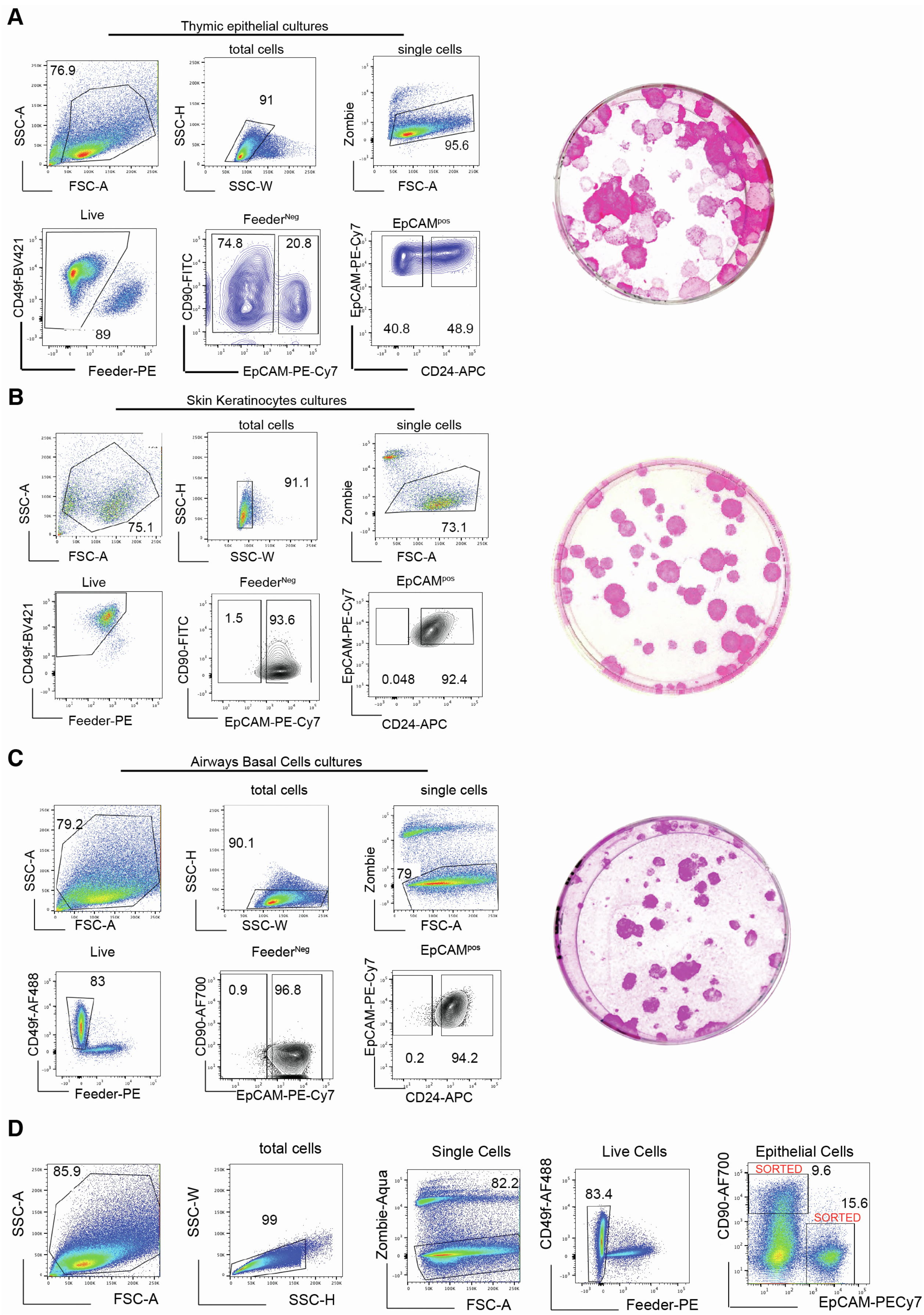
A
Thymic epithelial cultures
total cells
single cells
SSC-A
FSC-A
76.9
SSC-H
SSC-W
91
Zombie
95.6
Live
CD49f-BV421
Feeder-PE
89
FeederNeg
CD90-FITC
EpCAM-PE-Cy7
74.8
20.8
EpCAMpos
CD24-APC
40.8
48.9
B
Skin Keratinocytes cultures
total cells
single cells
75.1
91.1
73.1
Live
FeederNeg
EpCAMpos
1.5
93.6
0.048
92.4
C
Airways Basal Cells cultures
total cells
single cells
79.2
90.1
79
Live
FeederNeg
EpCAMpos
CD49f-AF488
83
CD90-AF700
0.9
96.8
0.2
94.2
D
total cells
Single Cells
Live Cells
Epithelial Cells
85.9
SSC-W
SSC-H
99
Zombie-Aqua
82.2
83.4
SORTED
9.6
15.6
SORTED
EpCAM-PECy7

Figure S6. FACS analysis of epithelial SC *in vitro*, related to Figure 5.

(A) Representative FACS analysis of thymic SC showing gating strategy for live cells analysis and CD49f, CD90 (THY1), EpCAM, CD24 distribution (n=8, donor-derived cultures). Right panel: rhodamine-B staining of clonogenic TEC: 500 cells were seeded in a dish for colony-forming efficiency (CFE) assay; the dish was fixed and stained with rhodamine-B after 12 days of culture. Cells gave rise to colonies of variable sizes that stained either strongly or dim with rhodamine-B (n=8, donor-derived cultures).

(B) Representative FACS analysis of expanding cultivated skin keratinocytes reveals a homogeneous population expressing CD49f, EpCAM, CD24 and negative for CD90. Right panel: rhodamine-B staining of skin keratinocytes, performed in the same conditions as per described in (A), showed strong rhodamine-B staining (n=3).

(C) Representative FACS analysis of expanding cultivated airways basal cells shows also a homogeneous population expressing CD49f, EpCAM, CD24 and negative for CD90. Right panel: rhodamine-B staining of airways basal cells, performed in the same conditions as described in (A), showed strong rhodamine B staining (n=3).

(D) Representative FACS analysis of gating strategy for thymic SC sorted cultures (n=8, donor-derived cultures).

Figure S7

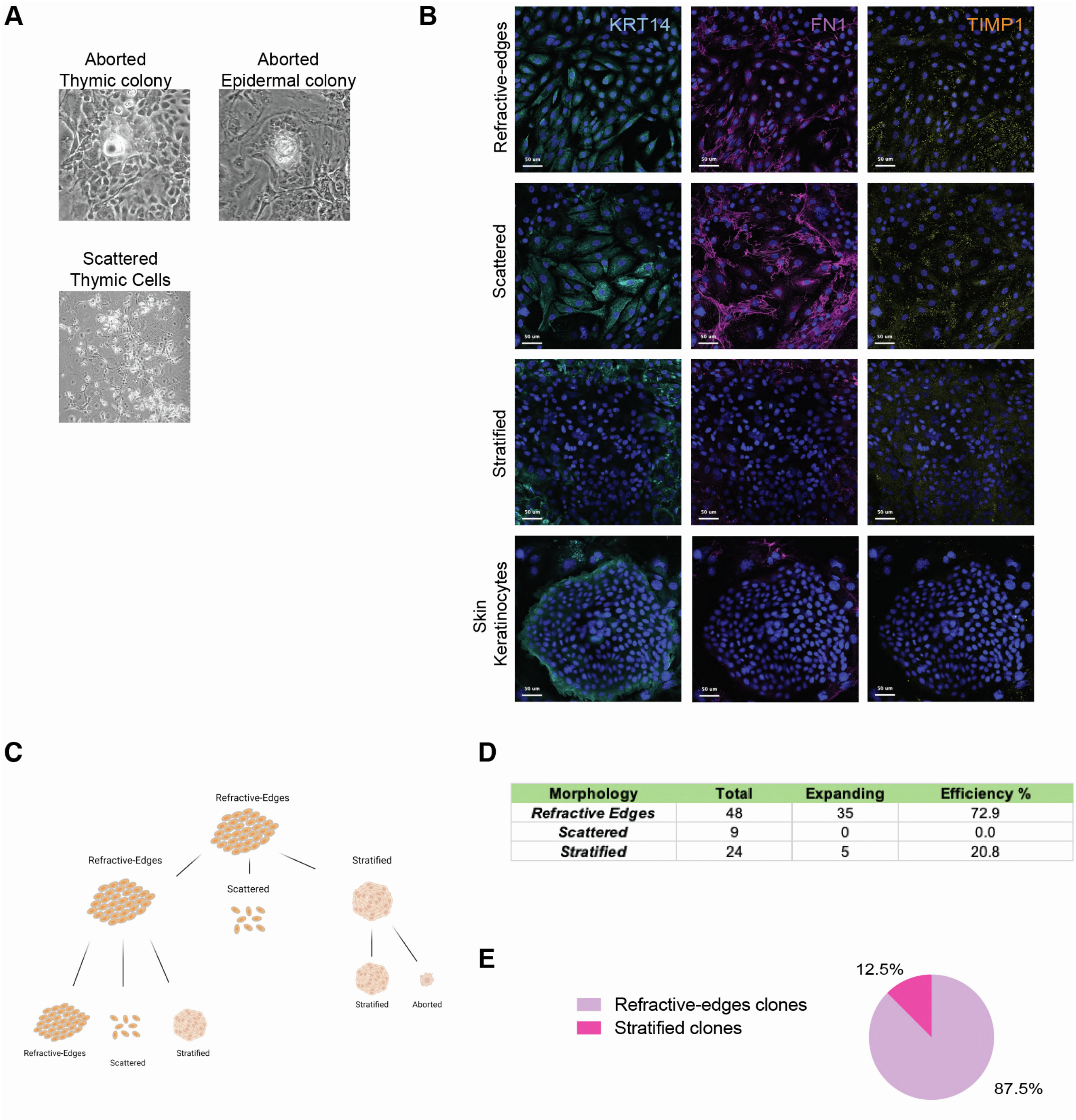

Morphology	Total	Expanding	Efficiency %
Refractive Edges	48	35	72.9
Scattered	9	0	0.0
Stratified	24	5	20.8

Figure S7. Single-cell cloning of thymic SCs, related to Figure 6.
(A) Phase contrast images of individual aborted thymic cells, keratinocyte colonies and thymic scattered cells, respectively.
(B) Immunofluorescence staining of cultivated TECs showing KRT14 (cyan), FN1 (magenta) and TIMP1 (metalloprotease inhibitor protein, in yellow) expression in refractive-edges and scattered, while FN1 and TIMP1 were not detected in stratified and keratinocyte colonies. Nuclei counterstained with DAPI. n=3, donor-derived cultures. Scale bars, 50 µm.
(C) Schematic representing the hierarchical relationship of thymic colonies created with *Biorender.com*.
(D) Table summarizing total number of single-cell clones that were amplified and relative efficiency of expansion per morphology type. Results include four independent cloning experiments (n=4, donor-derived cultures).
(E) Pie chart representing the percentage of expanding clones classified per morphology: light pink corresponds to refractive-edges colonies (87.5%), while purple to stratified clones (12.5%) in n=4 donor-derived cultures.

Figure S8

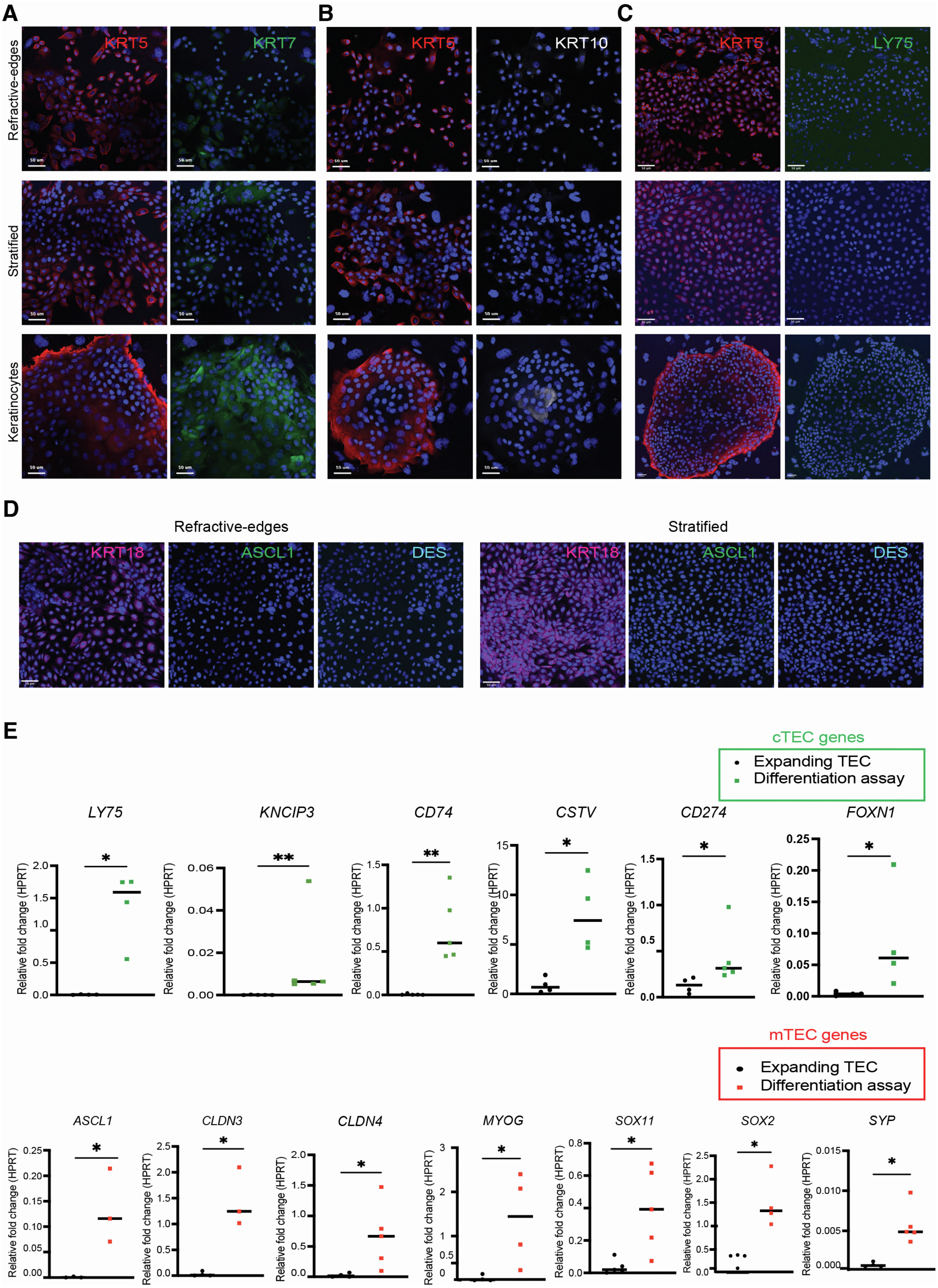

A
B
C
KRT5
KRT7
KRT10
LY75
Refractive-edges
Stratified
Keratinocytes
D
Refractive-edges
Stratified
KRT18
ASCL1
DES
E
cTEC genes
Expanding TEC
Differentiation assay
LY75
KNCIP3
CD74
CSTV
CD274
FOXN1
Relative fold change (HPRT)
mTEC genes
Expanding TEC
Differentiation assay
ASCL1
CLDN3
CLDN4
MYOG
SOX11
SOX2
SYP

Figure S8. Thymic SCs differentiate into both cortical and medullary fates *in vitro*, related to Figure 7.

(A) Immunofluorescence staining of cultivated thymi and keratinocytes showing expression of KRT5 (red) and KRT7 (green). Thymic SC do not express KRT7 in expansion, while they express KRT5**.** Scale bars, 50 µm.

(B) Immunofluorescence staining of cultivated thymic SC and keratinocytes showing expression of KRT5 (red) and KRT10 (white). Thymic SC do not express KRT10 in expansion, while they express KRT5. Scale bars, 50 µm.

(C) Immunofluorescence staining of cultivated TEC and keratinocytes showing expression of KRT5 (red) and LY75 (green). Neither TECs nor skin keratinocytes express LY75 in expansion. Scale bars, 50 µm.

(D) Immunofluorescence staining of cultivated thymic cells showing expression of KRT18 (magenta), ASCL1 (green) and DES (cyan). Thymic SC do not express either ASCL1 and DES proteins in expansion. Nuclei counterstained with DAPI. n=3, Scale bar, 50 µm**.**

(E) RT-qPCR analysis of expanded thymic cells in 2D expansion (black dots) versus differentiation showing upregulation of cortical genes (*LY75, KCNIP3, CD74, CSTV, CD274, FOXN1*) in green (top panel) and medullary genes (*ASCL1, CLDN3, CLDN4, MYOG, ,SOX11, SOX2, SYP*) in red (bottom panel). Relative gene expression to *HPRT* housekeeping is shown in y axis; significance: Mann-Whitney test, non-parametric; *p<0.05; **p<0.01, n=5, independent clones; mean ±S.E.M.

Figure S9

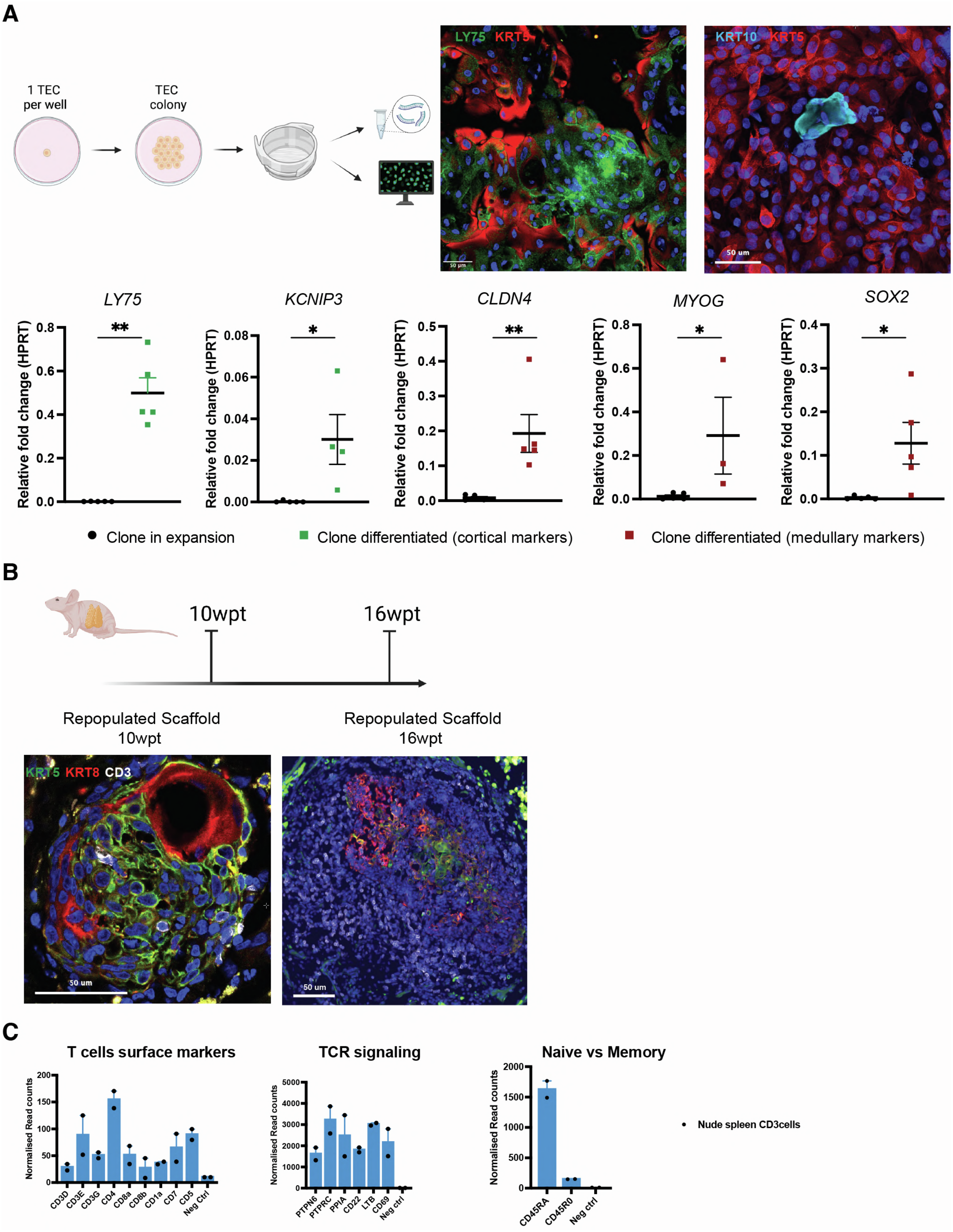
A
1 TEC per well
TEC colony
LY75 KRT5
KRT10 KRT5
50 um
LY75
KCNIP3
CLDN4
MYOG
SOX2
Relative fold change (HPRT)
Clone in expansion
Clone differentiated (cortical markers)
Clone differentiated (medullary markers)
B
10wpt
16wpt
Repopulated Scaffold 10wpt
Repopulated Scaffold 16wpt
KRT5 KRT8 CD3
50 um
C
T cells surface markers
TCR signaling
Naive vs Memory
Normalised Read counts
CD3D CD3E CD3G CD4 CD8a CD8b CD1a CD7 CD5 Neg Ctrl
PTPN6 PTPRC PPIA CD22 LTB CD69 Neg ctrl
CD45RA CD45R0 Neg ctrl
Nude spleen CD3cells

Figure S9. Thymic SCs differentiate into both cortical and medullary fates *in vitro* and *in vitro,* related to Figure 7.

(A) Schematic workflow for TEC differentiation assay from a single TEC clone. Image created with *Biorender.com*. TEC clone, differentiated after expansion, shows cells positive for KRT5 (red), cortical cells (LY75-positive, green) on the left and areas with Hassall's Bodies (HB) structures positive for KRT10 (cyan) on the right. Nuclei counterstained with DAPI. Scale bar, 50 µm (representative image, n=5, independent clones). Bottom panels: RT-qPCR analysis of cultivated TEC clones in 2D expansion (black dots) versus TEC clones after differentiation indicated upregulation of cortical genes (*LY75, KCNIP3*) in green dots and medullary genes (*CLDN4, MYOG, SOX2*) in red squares. Relative gene expression to *HPRT* housekeeping is shown in y axis; significance: Mann-Whitney test, non-parametric; *p<0.05; **p<0.01, n=5, independent clones; mean ±S.E.M.

(B) Schematic illustrating timeline of *in vivo* transplantation of scaffolds repopulated with TEC and TIC (thymic interstitial cells) into humanized nude athymic mice (top panel). Immunofluorescent images of thymic scaffold grafts sections (7µm) at week 10 and 16 post transplantation stained for human KRT5 (green), human KRT8-18 (red) and human CD3 (gray). CD3 cells progressively colonize (10 weeks) and increase their presence within repopulated human epithelial areas in the scaffolds by 16 weeks. Nuclei counterstained with DAPI. Scale bars, 50 µm (n=8, scaffolds per time point).

(C) Single gene expression profiles of T cell surface markers (left), TCR signaling components (centre) and naïve versus memory markers (right) expressed in $CD3^+$ cells sorted from spleen samples of grafted NSG-Nude mice and analyzed by nCounter NanoString (n=4 implanted scaffolds grouped for each time point).

Table S1. Pseudotime of thymic SC *in vitro,* related to Figure 5
Pseudotime Heatmap (Monocle) depicting the most time-variable genes along the single-cell trajectory from cluster C1 to C2 and C3. Single genes along the trajectory are indicated on the right side of the graph. Log_{10} expression, Pseudotime value and clusters are indicated in the legends on the right side. Cluster colors and Pseudotime are indicated at the top of x axes (N=8, thymus-derived cultures).

Table S2. Antibody List, related to STAR Methods

Table S3. qPCR probes, related to STAR Methods

Table S1. Pseudotime of thymic SC *in vitro*, related to Figure 5

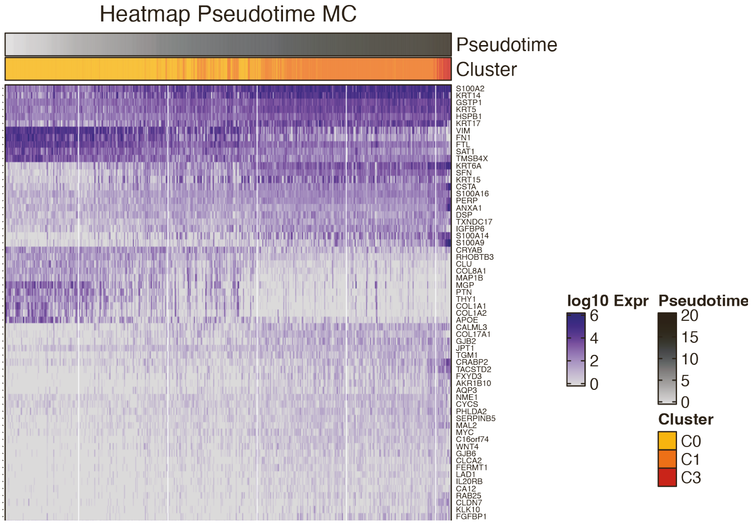

Table S1 related to STAR METHODS

FACS antibodies list

Antigen	Fluorocrome	Dilution	Company	Cat.No.
BCAM	BV421	1:100	BD Bioscience	748007
CD24	APC	1:100	BioLegend	311117
CD24	BV605	1:100	BioLegend	311124
CD205	PE	1:400	BioLegend	342203
CD235ab (glycophorin A and B)	Biotin	1:100	BioLegend	306618
CD45	Biotin	1:100	BioLegend	103104
CD45	APC	1:200	BioLegend	304011
CD49f	AF488	1:200	BioLegend	313608
CD49f	BV421	1:200	BioLegend	313624
CD90	AF700	1:200	Biolegend	328119
CD90	FITC	1:200	Biolegend	328108
EpCAM (CD326)	PE-Cy7	1:100	BioLegend	324222
Feeder	PE	1:100	Miltenyi	130120166
Zombie Aqua™ Fixable Viability Kit		1:200	Biolegend	423102
DAPI		1:300	Sigma	D9542

Primary and Secondary Antibodies list

Antigen	Host	Dilution	Company	Cat.No.
CD49f- AF488 conjugated	Rat	1:100	Biolegend	313608
Ly75 (CD205)	Rabbit	1:100	Abcam	AB124897
CK5	Mouse	1:100	Abcam	AB17130
CK7	Rabbit	1:100	Sigma Aldrich	HPA007272-100UL
CK10	Mouse	1:200	Santa Cruz	SC-53252
CK13	Mouse	1:100	Abcam	AB16112
CK14	Chicken	1:200	Biolegend	905301
CK15	Rabbit	1:100	Sigma Aldrich	HPA023910
CK17	Rabbit	1:100	Sigma Aldrich	HPA000453
CK8	Mouse	1:50	Abcam	AB9023
CK8/18	Guinea pig	1:100	Acris/2BeScientific	BP5075
EpCAM- efluor660 cojugated	Mouse	1:100	eBioscience	50-9326-42
p63	Mouse	1:50	Abcam	AB735
IFITM3	Rabbit	1:100	Thermofisher	MA5-32798
FN1	Rabbit	1:300	proteintech	15613-1-AP
THY-1 (CD90)	Mouse	1:100	Biolegend	328108
deltaNTP63alpha	Rabbit	1:100000		kindly donated by Michele De Luca
BCAM	Mouse	1:100	BD Bioscience	748007
BCAM	Rabbit	1:100	Novus Biological	NBP2-31994
CD24-APC conjugated	Mouse	1:100	BioLegend	311117
ASCL1	Rabbit	1:100	Abcam	AB74065
TIMP1	Mouse	1:50	Invitrogen	MA1-773
SOX2	Goat	1:100	R&D Systems	AF2018
CD45	Rabbit	1:200	Abcam	AB40763
CD45	Mouse	1:100	Novus Biological	NBP2-34528
CD3E	Mouse	1:100	Origene	UM500048
CD3 APC conjugated	Mouse	1:100	BioLegend	300411
Desmin	Mouse	1:100	Agilent/DAKO	M0760

Secondary Antibodies

Anti-Guinea Pig AF594	Donkey	1:500	Jackson Immuno	706-165-148
Anti-Mouse AF488	Donkey	1:500	Jackson Immuno	715-545-150
Anti-Mouse AF594	Donkey	1:500	Jackson Immuno	715-165-150
Anti-Mouse AF647	Donkey	1:500	Jackson Immuno	715-605-150
Anti-Rabbit AF488	Donkey	1:500	Jackson Immuno	711-545-152
Anti-Rabbit AF594	Donkey	1:500	Jackson Immuno	711-585-152
Anti-Rabbit AF647	Donkey	1:500	Jackson Immuno	711-605-152
Anti-Rat AF488	Donkey	1:500	Jackson Immuno	712-545-150
Anti-Rat AF647	Donkey	1:500	Jackson Immuno	712-605-150
Anti-Chicken AF488	Donkey	1:500	Jackson Immuno	703-225-155
Anti-Chicken AF594	Donkey	1:500	Jackson Immuno	705-585-155
Anti-Goat AF488	Donkey	1:500	Jackson Immuno	705-545-147
Anti-Goat AF594	Donkey	1:500	Jackson Immuno	705-165-147

Table S2 related to STAR METHODS

qPCR probes list

Gene	Assay name	Probe	Primer 1	Primer 2
HPRT1	Hs.PT.SSv.45621572	5'-/56-FAM/ AGCCT AAGATGAGAGTTCAAGTTGAGTTTGG /36-TAMSp/-3'	5'-TTGTTGT AGGAT ATGCCCTTGA-3'	5'-GCGATGTCAATAGGACTCCAG-3'
LY75	Hs.PT.583240196	5'-/56-FAM/ TGATAGCTATTGCTGTGTAATCAGGGCC /36-TAMSp/-3'	5'-CCGCCCATGAGAACTAAGATAC-3'	5'-GGTTTTGGAAGAGTTGTCTGC-3'
KCNIP3	Hs.PT.58.20750533	5'-/56-FAM/ CCTGGGCGCTGAAGAAAACTGTTG /36-TAMSp/-3'	5'-TGTGAAGTGGGGAGGCT-3'	5'-ACTTGATACCCTCCTTCTTGC-3'
CD74	Hs.PT.58.4651852	5'-/56-FAM/ CCAGCGCGACCTT ATCTCCAACA /36-TAMSp/-3	5'-GGAAGATCAGAAGCCAGTCAT-3'	5'-CAGAGTCACCAGGATGGAAA-3'
CTSV	Hs.PT.58.370453	5'-/56-FAM/ AGCCAACCACCAGAACACCATGAT /36-T AMSp/-3'	5'-CCATTCGTCCTTCCAGTTCTAC-3'	5'-TTGACGAGCCAATACTTGCT-3'
CLDN4	Hs.PT.58.1326185.g	5'-/56-FAM/ ACCAGTTCCTTCCAGCCGACAG /36-TAMSp/-3'	5'-CCATATAACTGCTCAACCTGTCC-3'	5'-AGATAAAGCCAGTCCTGATGC-3'
MYOG	Hs.PT.58.38897870	5'-/56-FAM/ ATGCCCGGCTTGGAAGACAATCT /36-TAMSp/-3'	5'-GACAGCATCACAGTGGAAGA-3'	5'-AGAAGTAGTGGCATCTGTGG-3'
SOX2	Hs.PT.58.237897.g	5'-/56-FAM/ CACCTACAGCATGTCCTACTCGCA /36-TAMSp/-3'	5'-GTACAACTCCATGACCAGCTC-3'	5'-CTTGACCACCGAACCCAT-3'
SYP	Hs.PT.58.27207712	5'-/56-FAM/ TGTACTTTGATGCACCCACCTGCC /36-TAMSp/-3'	5'-AGTCCCCAACTAAGAAGACCT-3'	5'-ACAAGACCGAGAGTGACCT-3'
ASCL1	Hs.PT.56a.23464.gs	5'-/56-FAM/ CGATCACCCTGCTTCCAAAGTCCA /36-TAMSp/-3'	5'-AGCTTCTCGACTTCACCAAC-3'	5'-CAACGCCACTGACAAGAAAG-3'
CLDN3	Hs.PT.58.45414673.g	5'-/56-FAM/ TCCACAGGCCCTCCCAGATG /36-TAMSp/-3'	5'-CAACATCATCACGTCGCAGA-3'	5'-GAGTCGTACACCTTGCACTG-3'
SOX11	Hs.PT.58.2452961 5.g	5'-/56-FAM/ ACACAACAGCCTCACCACCGAA /36-TAMSp/-3'	5'-ACTGAT AGAAACTCGCATCGC-3'	5'-GAATTCTCTCCTGCCACCTC-3'
CD274	Hs.PT.58.20308441	5'-/56-FAM/ CCCAAGGACCTATATGTGGTAGAGTATGGT /36-TAMSp/-3'	5'-CTACTGGCATTTGCTGAACG-3'	5'-CCAATAGACAATTAGTGCAGCCA-3'
FOXN1	Hs.PT.58.40389553	5'-/56-FAM/ TCTCGCTGACGGGAAAGGCTC /36-TAMSp/-3'	5'-CCCAAAACCCATCTATTCCTACA-3'	5'-GTCTTGAAGTAAGGAAAGTGCTC-3'
