# Supplementary material for: Defining the identity and the niches of epithelial stem cells with highly pleiotropic multilineage potency in the human thymus
Source: Dev Cell. 2023 Nov 20;58(22):2428–2446.e9. doi: 10.1016/j.devcel.2023.08.017 (PMC10957394; doi:10.1016/j.devcel.2023.08.017)
Supplement: Document S2. Article plus supplemental information [file mmc2.pdf]

Developmental Cell

Article

Defining the identity and the niches of epithelial stem cells with highly pleiotropic multilineage potency in the human thymus

Graphical abstract

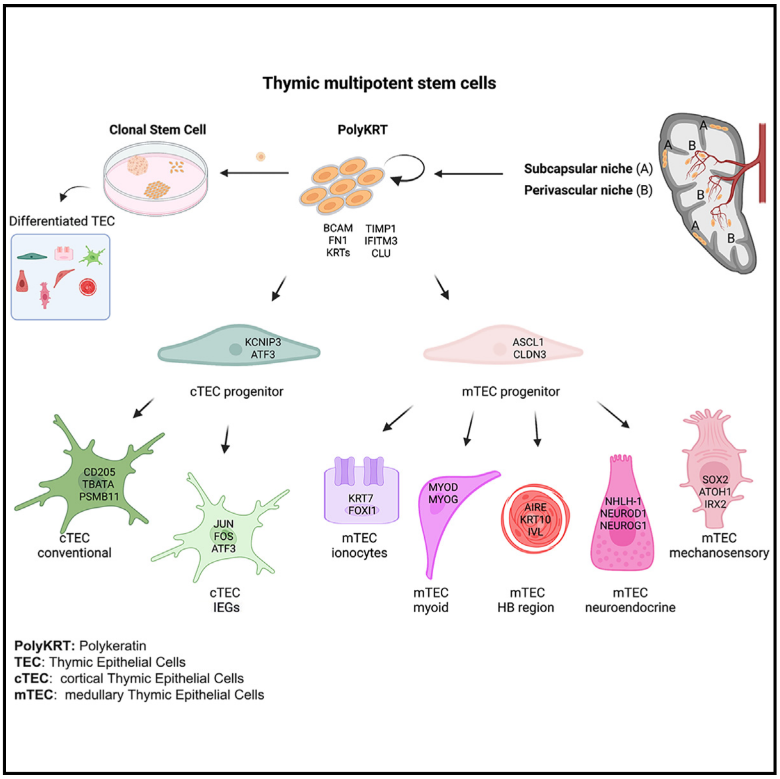

Highlights

- The human thymus contains *bona fide* epithelial SCs with atypical signature
- Subcapsular and perivascular spaces are the niches of multipotent thymic SCs
- Thymic SCs extensively self-renew *in vitro* and show diverse clone morphologies
- Cultured SCs retain multilineage differentiation potency and self-organize *in vitro*

Authors

Roberta Ragazzini, Stefan Boeing, Luca Zanieri, ..., Adrian C. Hayday, Dominique Bonnet, Paola Bonfanti

Correspondence

p.bonfanti@ucl.ac.uk

In brief

Ragazzini et al. identify pleiotropic multipotent stem cells (SCs) and define their cortical and medullary niches in human pediatric thymi by combining scRNA-seq, spatial transcriptomics, and multiplex phenotyping. Thymic SCs display Polykeratin traits, extensively expand as clones in culture, and retain self-organizing capacity upon functional differentiation.

Ragazzini et al., 2023, Developmental Cell *58*, 2428–2446
November 20, 2023 © 2023 The Author(s). Published by Elsevier Inc.
https://doi.org/10.1016/j.devcel.2023.08.017

CellPress
OPEN ACCESS

Developmental Cell

Article

Defining the identity and the niches of epithelial stem cells with highly pleiotropic multilineage potency in the human thymus

Roberta Ragazzini,[1,2] Stefan Boeing,[3] Luca Zanieri,[1,2] Mary Green,[4] Giuseppe D'Agostino,[5,6] Kerol Bartolovic,[7] Ana Agua-Doce,[7] Maria Greco,[8] Sara A. Watson,[1] Antoniana Batsivari,[5] Linda Ariza-McNaughton,[5] Asllan Gjinovci,[1,2] David Scoville,[9] Andy Nam,[9] Adrian C. Hayday,[10,11] Dominique Bonnet,[5] and Paola Bonfanti[1,2,12,*]
[1]Epithelial Stem Cell Biology & Regenerative Medicine Laboratory, The Francis Crick Institute, 1 Midland Road, London NW1 1AT, UK
[2]Institute of Immunity & Transplantation, Division of Infection & Immunity, UCL, Pears Building, Rosslyn Hill, London NW3 2PP, UK
[3]Bioinformatics & Biostatistics, The Francis Crick Institute, 1 Midland Road, London NW1 1AT, UK
[4]Experimental Histopathology Laboratory, The Francis Crick Institute, 1 Midland Road, London NW1 1AT, UK
[5]Haematopoietic Stem Cell Laboratory, The Francis Crick Institute, 1 Midland Road, London NW1 1AT, UK
[6]Plasticell Limited, Stevenage Bioscience Catalyst, Gunnels Wood Road, Stevenage SG1 2FX, UK
[7]Flow Cytometry Core, The Francis Crick Institute, 1 Midland Road, London NW1 1AT, UK
[8]Single Cell Facility, MRC WIMM, University of Oxford, Oxford OX3 9DS, UK
[9]NanoString Technologies Inc., Seattle, WA, USA
[10]Immunosurveillance Laboratory, The Francis Crick Institute, 1 Midland Road, London NW1 1AT, UK
[11]Peter Gorer Department of Immunobiology, School of Immunology & Microbial Sciences, King's College London, London, UK
[12]Lead contact
*Correspondence: p.bonfanti@ucl.ac.uk
https://doi.org/10.1016/j.devcel.2023.08.017

SUMMARY

Thymus is necessary for lifelong immunological tolerance and immunity. It displays a distinctive epithelial complexity and undergoes age-dependent atrophy. Nonetheless, it also retains regenerative capacity, which, if harnessed appropriately, might permit rejuvenation of adaptive immunity. By characterizing cortical and medullary compartments in the human thymus at single-cell resolution, in this study we have defined specific epithelial populations, including those that share properties with *bona fide* stem cells (SCs) of lifelong regenerating epidermis. Thymic epithelial SCs display a distinctive transcriptional profile and phenotypic traits, including pleiotropic multilineage potency, to give rise to several cell types that were not previously considered to have shared origin. Using here identified SC markers, we have defined their cortical and medullary niches and shown that, *in vitro*, the cells display long-term clonal expansion and self-organizing capacity. These data substantively broaden our knowledge of SC biology and set a stage for tackling thymic atrophy and related disorders.

INTRODUCTION

Epithelial tissues constantly renew during development, homeostasis, and regeneration, albeit at different rates. These processes are driven by self-renewing epithelial stem cells (SCs), the only *bona fide* SCs that can to date be extensively expanded *in vitro*.[1–7] Maintenance of stemness largely relies on SC cross-talk with their specialized *in vivo* microenvironment, known as the niche. Co-cultures with irradiated feeder fibroblasts for stratified epithelial SCs or Paneth cells for intestinal SCs provide evidence of the importance of the niche signals to expand functional SCs for several generations *in vitro*.[8,9] With rare exceptions (i.e., *LGR5* in the gut crypt epithelium), most *bona fide* epithelial SCs do not present unique markers but rather are defined by multiple phenotypic and functional features.[10–12] Nonetheless, high expression of the TP63 transcription factor (TF)—especially its ΔNTP63α isoform—has been correlated with epithelial stemness.[13–15] More recently, a transcriptional signature has been reported for the epidermal "holoclone"—i.e., the keratinocyte clonogenic SCs that are capable of self-renewal *in vitro* and *in vivo*.[6,16]

The thymus stands out among organs for the unique three-dimensional (3D) morphological complexity of its epithelium and for its undergoing progressive atrophy during postnatal life that is, however, reversible.[17,18] The essential capacity of the thymus to generate and select for a diverse yet self-tolerant T cell repertoire reflects critical spatial and temporal interactions of developing thymocytes with the thymic stroma. The stroma is itself highly complex, comprising different types of thymic epithelial cells (TECs) (medullary thymic epithelial cells [mTECs] and cortical thymic epithelial cells [cTECs]); myoid and neuroendocrine cells; thymic interstitial cells; endothelial cells; and

This is an open access article under the CC BY license (http://creativecommons.org/licenses/by/4.0/).

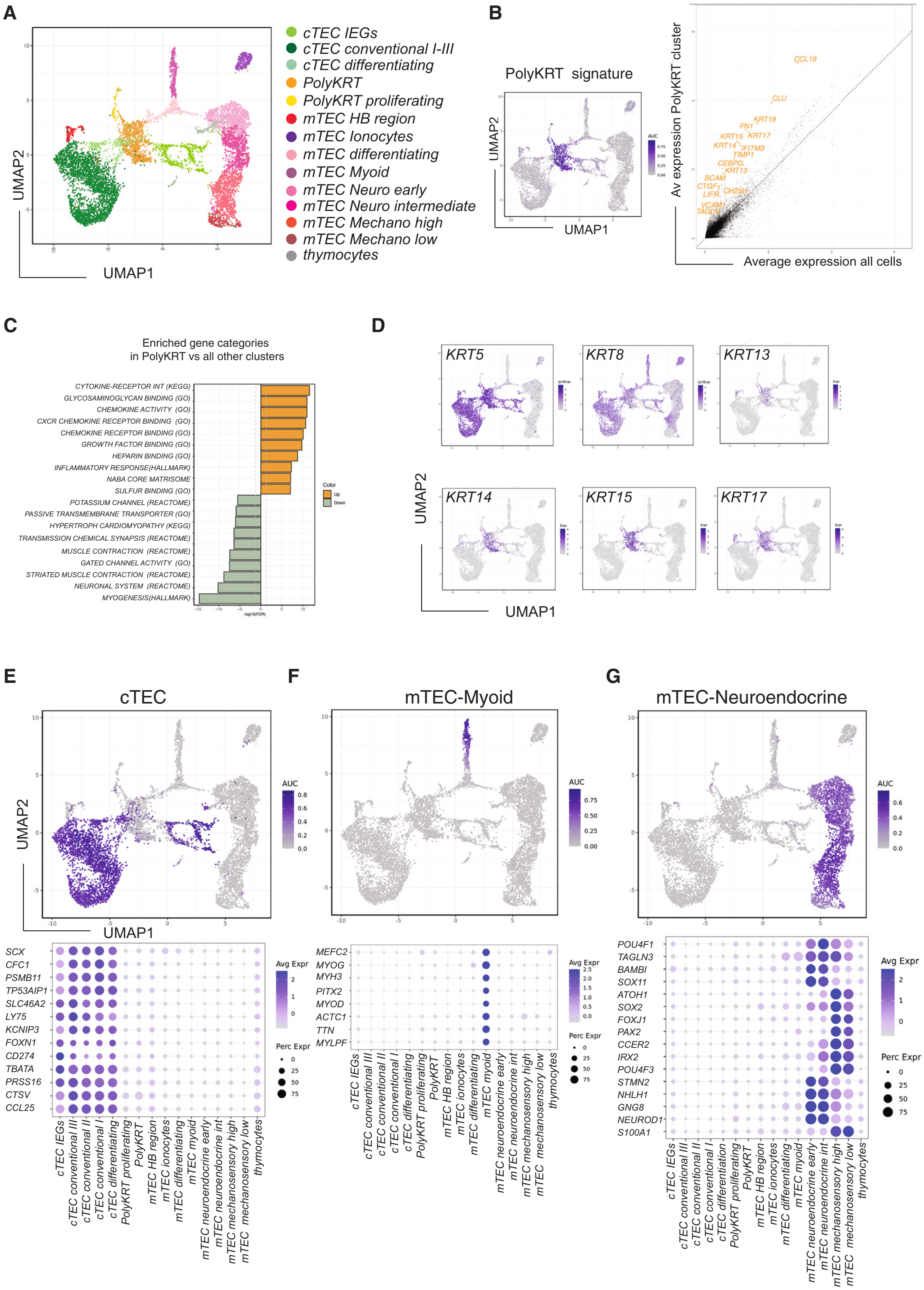

(legend on next page)

hematopoietic subtypes such as dendritic cells, B cells, and macrophages. Hematopoietic cells colonize the epithelial-interstitial thymic anlagen during development and promote lympho-stroma crosstalk that orchestrates both thymocyte development and epithelial differentiation and morphogenesis. Failure of thymic epithelial specification during development results in congenital thymic agenesis and leads to severe combined immunodeficiency and autoimmunity.[19,20]

Embryonic thymic epithelial progenitors and their differentiation into cTECs and mTECs have been extensively studied using lineage tracing, reporter systems, and transplantation in mouse models.[21,22] Recently, distinct cortical and medullary bipotent progenitors were identified based on their developmental dynamics via CRISPR-Cas9 barcoding in mice supporting progenitor heterogeneity.[23] By contrast, the mechanisms contributing to postnatal thymus repair and regeneration are very poorly understood, with the origin of the multiple stromal subtypes that shape the tolerogenic medullary microenvironment remaining unelucidated.[24] Current models for thymic regeneration that may revert its involution are based on inducing injury (e.g., by irradiation and lymphocyte depletion) or modulating hormonal regulation (castration).[25] The underpinning mechanisms of these procedures and their pathophysiologic relevance are uncertain. Nonetheless, tissue regeneration implies the existence of long-lasting, self-renewing SCs.

Indeed, despite reports of various putative epithelial progenitors and claims regarding marker genes to identify them,[22,26–31] the identification of SCs in the postnatal human thymus has not been achieved.[32] To address this, we combined high-resolution *in vivo* and *in vitro* single-cell analysis with prospective isolation, clonal expansion, and differentiation assays. We integrated these findings with spatial phenotyping and transcriptomics. This has permitted the identification of postnatal human thymic SCs, the resolution of their niches, and a comprehensive definition of specialized TECs in the postnatal cortex and medulla, providing important insights into mechanisms driving thymic epithelial regeneration and multilineage specification. In addition to those insights into thymus biology, these findings have implications in addressing disorders of the adaptive immune system and its functional decline with age.

RESULTS

Identification of a thymic cell population with an atypical epithelial signature and stemness traits

Although single-cell RNA sequencing (scRNA-seq) allows cellular heterogeneity to be defined in an organ microenvironment, full characterization of thymic stroma is confounded by several factors, including the limiting representation of epithelial cells (<0.02%) that need to be efficiently isolated from an organ where over 99% of the cells are developing thymocytes.[33–36] To identify all epithelial cells of the postnatal thymus at high resolution, we performed independent scRNA-seq analysis of cortical and medullary populations that were sorted based on EpCAM^{low}CD205^{pos} (cortex) and EpCAM^{high}CD205^{neg} (medulla) after several rounds of stromal cell enrichment (Figure S1A). All TECs were visualized in a UMAP (uniform manifold approximation and projection) plot where cTEC clusters are depicted in light/dark green colors and mTEC clusters in pink/red (Figure 1A). The most upregulated genes for each of these 16 clusters are shown in a comprehensive cluster marker heatmap (Figure S1B)

Independent scRNA-seq of each of the cTEC and mTEC sorted populations allowed us to define a specific cluster present in both cortex and medulla that is visualized in orange in the center of the UMAP plot (Figure 1A). We then studied the transcriptional profile of this cluster and defined a distinctive signature that is visualized in the average gene expression scatter plot, in the feature view UMAP (Figure 1B), and in the volcano plot (Figure S1C). Of note, transcripts of several genes encoding for extracellular matrix (ECM) proteins or molecules for anchoring to ECM (including fibronectin [*FN1*], *TIMP1,* basal cell adhesion molecule [*BCAM*], and *VCAM1*) argued that these cells could produce components contributing to their own niche (Figure 1B). In addition, we noted expression of genes mediating anti-viral response (i.e., *CH25H* and *IFITM3*), which confers high viral infection resistance to various tissue SCs.[37] Enrichment analysis of this cluster versus all other thymic clusters confirmed the activation of ECM-binding as well as of inflammatory response pathways in these cells (Figure 1C). Strikingly, genes such as *CEBPD, CLU*, and *LIFR* involved in cell-cycle regulation and self-renewal of limbal, intestinal, and embryonic SC were also featured genes of this cluster (Figure 1B).[38–40]

In addition, these cells expressed multiple cytokeratins (KRTs), which ordinarily define the specific lineage differentiation of simple, stratified, and glandular epithelial cell types, respectively, found in different tissues.[41,42] The expressed keratins included *KRT5*, *KRT8*, *KRT13*, *KRT14*, *KRT15*, *KRT17*, *KRT18*, and *KRT19*. Of note, cells in this cluster co-expressed KRTs that in other tissues are associated with either proliferating SC (e.g., *KRT15*) or differentiated layers (e.g., *KRT13*) (Figures 1B, 1D, S1B, and S1C). They also co-expressed KRTs that are usually found only in simple (e.g., *KRT8/18*) or in basal layers (e.g., *KRT5/14*) of stratified epithelia.[43] The KRT profile of this cluster shows unprecedented breadth, but was nonetheless selective, as indicated by the finding that some KRTs that characterize other differentiated cell types, e.g., *KRT7* (lung ionocytes [Io])

Figure 1. Human postnatal thymic epithelial cells (TECs) are highly heterogeneous

(A) UMAP plot visualization of TECs colored by cell cluster group.

(B) UMAP category feature view plot of Polykeratin (PolyKRT) cluster marker genes $\log_{10}$ expression (left); average expression of PolyKRT markers identified by linear regression in orange against expression in all the other cells (right): *KRT13*, *KRT14*, *KRT15*, *KRT17*, *KRT19*, *CCL19*, *CEBPD*, *CLU*, *FN1*, *IFITM3*, *TIMP1*, *VCAM1*, *CTFG*, *TAGLN*, *BCAM*, *LIFR*, and *CH25H*.

(C) Category enrichment analysis of differentially expressed genes (DEGs) of PolyKRT cluster versus all the other clusters. Color code illustrates ten mostly upregulated categories in PolyKRT cluster (orange) and downregulated ones in gray. Hypergeometric test was performed on the top upregulated and downregulated genes to identify overrepresented gene categories.

(D) UMAP visualization of $\log_{10}$ expression of cytokeratin genes (*KRT5*, *KRT8*, *KRT13*, *KRT14*, *KRT15*, and *KRT17*) expressed in the PolyKRT cluster.

(E–G) UMAP category feature view plots and dot plots showing gene category for cTEC, mTEC-myoid, mTEC-neuroendocrine-early/intermediate, and mTEC-mechanosensory-high/low clusters.

CellPress
OPEN ACCESS

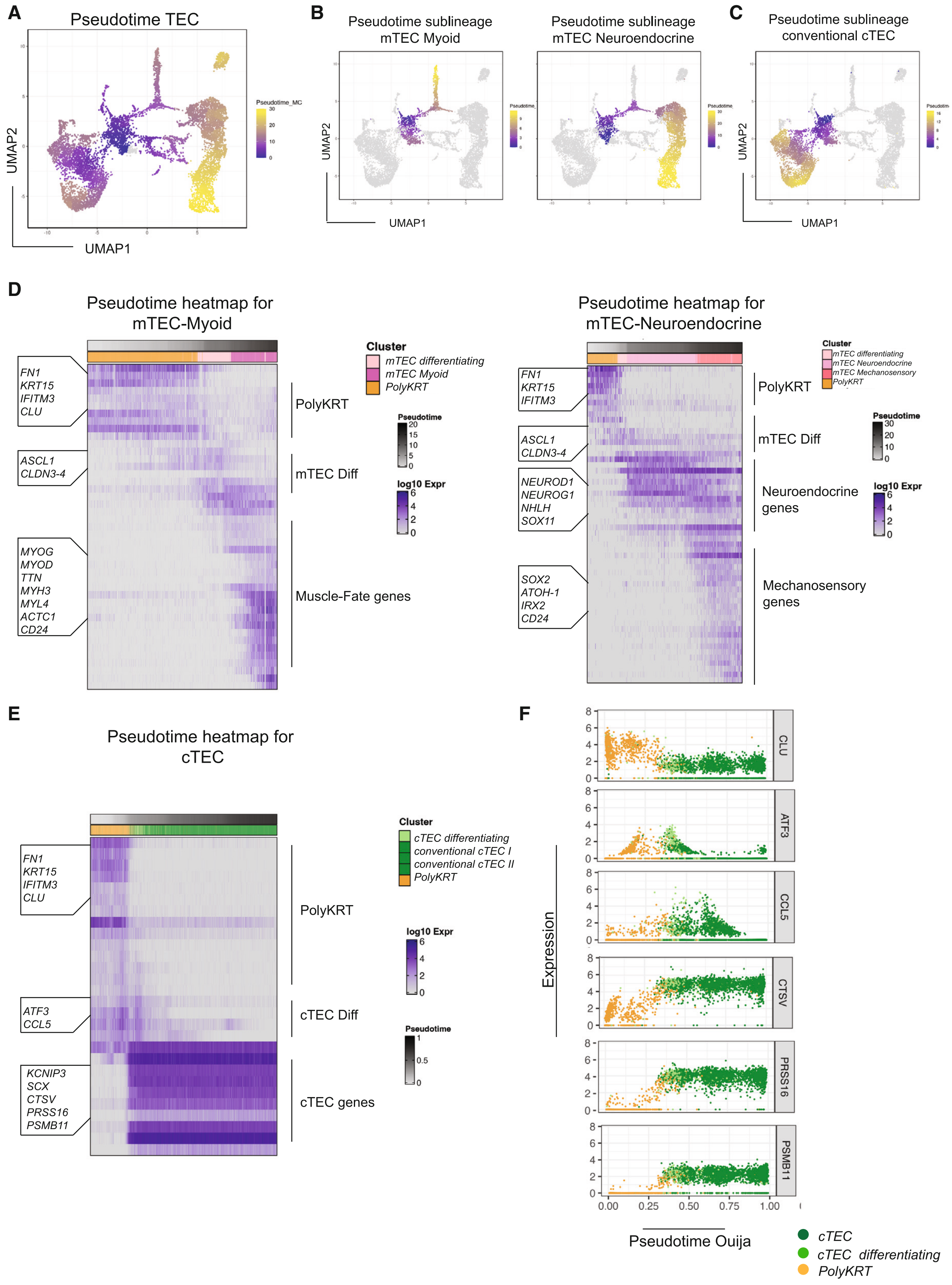

Figure 2. Single-cell trajectory analysis reveals that PolyKRT cells differentiate into specialized mTECs and cTECs

(A and B) UMAP plot of cells colored according to pseudotime analysis run with Monocle software: PolyKRT cells differentiate toward cortical and medullary clusters. PolyKRT cells differentiate along trajectory toward mTEC-myoid (left) and mTEC-neuroendocrine/-mechanosensory clusters (right).

(legend continued on next page)

and *KRT1/KRT10* (upper layers of epidermis), were expressed only by specialized thymic clusters, i.e., Io and cornified Hassall's body (HB), respectively (Figures S1D and S1E). Thus, we named the cluster Polykeratin (PolyKRT) rather than pankeratin.

A second small cluster shared PolyKRT genes but was also characterized by genes related to active proliferation and holoclone signature[16] (Figures 1A and S1F). We hypothesized that PolyKRT cells and PolyKRT-proliferating cells might jointly represent putative SCs of the postnatal thymus.

High-resolution scRNA-seq defines diverse functional epithelial clusters

Before studying the putative SC populations further, we needed to define the identities and gene signatures of all other clusters in the cortex and medullary datasets. First, we found four cortical clusters that were clearly identified by a functional cortical cell signature (e.g., *TBATA*, *PRSS16*, *CTSV*, and *KCNIP3*) as shown in the feature view UMAP and dot plots (Figure 1E). Conventional cTECs I–III confirmed cortical clusters that were previously described by us and others,[33–35] whereas identification of the fourth cTEC cluster reflected the higher level of resolution of this study. When we determined the marker genes of this cluster, we identified a group of immediate-early genes (IEGs) (i.e., *JUN, FOS, ATF3*—so-called AP-1 family genes—and *EGR1*), associated with rapid responses to regulatory signals, such as immune responses or cellular stress[44] (Figure S1G). This signature may reflect a specific functional status of cortical cells (cTEC-IEGs), which also express high levels of *CD274* (encoding PD-L1) in addition to genes established as contributing to cortical function, e.g., *FOXN1, TBATA*, and *LY75* (Figure 1E).[45] Of note, the AP-1 family (c-JUN, c-FOS, and ATF3) has been previously implicated in CD274 (PD-L1) gene regulation in relation to its upregulation in thymic and other solid neoplasia.[46–48]

Second, we defined seven specialized medullary cell types that we grouped into five categories: (1) myoid cells (mTEC-myoid), characterized by molecules and TFs of smooth, skeletal, and cardiac muscle cells, i.e., *MYH3*, *MYOD*, *MYOG*, and *TTN* (Figure 1F). (2) Io were defined by, among other genes, *CFTR*, *FOXI1*, and *KRT7*, evoking the CFTR-expressing pulmonary Io population[49] (Figure S1D). (3) HB-region cluster cells, which are crucial for tolerance induction, were defined by the expression of *KRT1*, *KRT6A*, *KRT10*, *AIRE*, and *FEZF2* (Figure S1E). Finally, (4) and (5), two main groups of neuroendocrine (*NEUROD1*, *NHLH1*, *STMN2*, *GNG8*, and *NLRP1*) and mechanosensory (*ATOH1*, *IRX2*, *SOX2*, *PAX2*, *POU4F3*, *CCER2*, and *S100A1*) cells were identified. We studied the heterogeneity of these clusters: mTEC-neuroendocrine-early characterized by the preferential expression of *SOX11*, *NKX6-2*, *MGP*, and *AVP* and mTEC-neuroendocrine-intermediate by the expression of *POU4F1*, *HIGD1B*, and *GKAP1*. Similarly, we named mTEC-mechanosensory-high and mTEC-mechanosensory-low on the basis of the *SOX2* expression level (Figure 1G). These clusters displayed distinct signatures at the single-cell level, thus representing different *bona fide* neuroendocrine cell types within the thymus gland.

In short, we have identified several specialized cells and putative SCs in addition to two "transition" clusters that projected from the PolyKRT cluster toward either medullary or cortical differentiated cells in the UMAP plot (Figures 1A and S1H).

Multilineage differentiation of thymic epithelial PolyKRT cells

To begin to test our hypothesis that the PolyKRT clusters comprised human postnatal thymic SCs, we sought to investigate their differentiation toward cells with pre-medullary and pre-cortical progenitor signatures. By single-cell trajectory analysis (pseudotime), we obtained a UMAP plot with an unsupervised Monocle algorithm that highlighted different trajectories from the PolyKRT cluster toward all specialized cell types via the two transition clusters (Figures 2A–2C).

The pseudotime heatmaps detail sequential steps of differentiation with progressive gene regulation patterns. The heatmaps clearly displayed differentiation of the PolyKRT cluster toward medullary mTEC-myoid, mTEC-neuroendocrine, mTEC-mechanosensory, mTEC-Io, and mTEC-HB fates (Figures 2D and S2A). Therefore, we studied which genes were upregulated in mTEC-myoid, mTEC-neuroendocrine, and mTEC-mechanosensory cells derived from the mTEC-transitioning cluster characterized by *ASCL1, CLDN3*, and *CLDN4* upregulation (Figures 2D and S2B). The expression of TF *ASCL1* is notable in that it plays a key role for activating neuronal pathways[50]; thus, it was an unexpected trait of a progenitor population differentiating into both myoid and neuroendocrine fates. We therefore confirmed ASCL1 protein expression in the postnatal thymus medulla by using immunohistochemistry (IHC) (Figure S2C). Interestingly, the trajectory toward the Io cluster also passed through the mTEC-transition cluster and similarly expressed *ASCL1, CLDN3*, and *CLDN4* (Figure S2A), supporting the view that mTEC-myoid, mTEC-neuroendocrine, mTEC-mechanosensory, and mTEC-Io cell types all derived from a common mTEC progenitor.

Conversely, trajectories toward the HB region appeared to adopt a more direct differentiation path, with enhanced expression of genes already expressed in the PolyKRT cluster and with progressive acquisition of a cornified epithelial phenotype that is typical of stratified HB cells (Figure S2A). We noticed that *CD24* was expressed in all mTEC differentiated clusters (HB region, Io, mTEC-transition, mTEC-neuroendocrine, mTEC-mechanosensory, and mTEC-myoid clusters), with its broad medullary-specific expression confirmed by IHC and contrasting with the more restricted expression of ASCL1 in sparsely represented cells within the same region (Figures S2C and S2D).

(C) PolyKRT cells differentiate toward cortical clusters.
(D) Pseudotime heatmap depicting the most time-variable genes along the single-cell trajectory from PolyKRT to mTEC-differentiating, mTEC-myoid, mTEC-neuroendocrine/-mechanosensory clusters. Highlighted genes along the trajectory and the category of genes are indicated on the left and right side of the graph, respectively.
(E) Ouija pseudotime heatmap indicating the trajectory from PolyKRT to cTEC clusters.
(F) Single-gene $\log_{10}$ expression plots are showed for relevant genes categories, expressed along Ouija pseudotime trajectory: PolyKRT (*CLU*), cTEC-differentiating (*ATF3* and *CCL5*), and mature cTECs (*CTSV*, *PRSS16*, and *PSMB11*).

CellPress
OPEN ACCESS

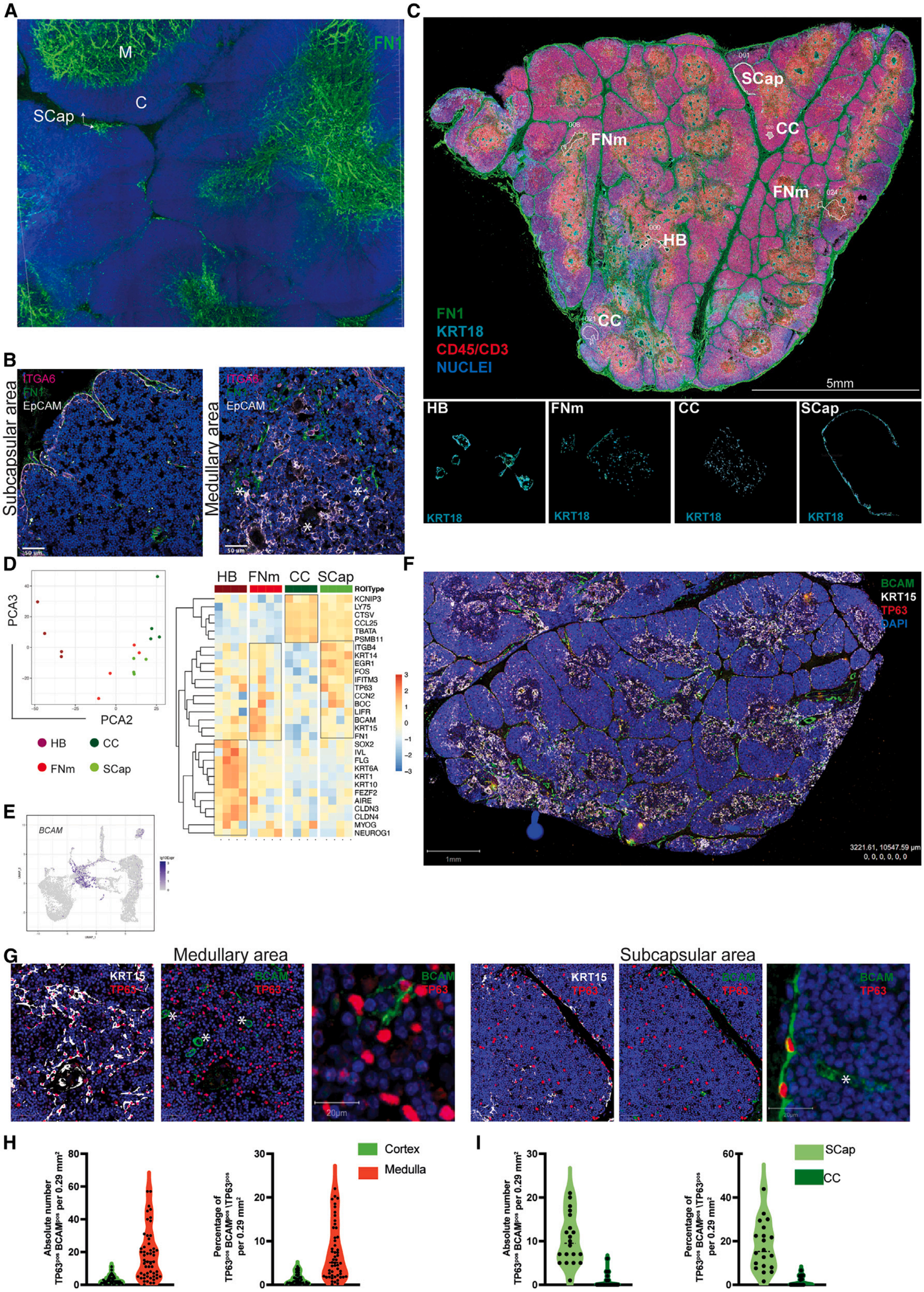

Figure 3. Thymic SCs reside within the subcapsular and perivascular niches and express BCAM

(A) 3D reconstruction of thick sections (300 μM) of human postnatal thymus stained with fibronectin (FN1, green). FN1 stains highly dense tubular structures in the medulla (M) and subcapsular (SCap) regions in the cortex (C). Nuclei are counterstained with 4′,6-diamidino-2-phenylindole (DAPI). Scale bars, 200 μm.

(legend continued on next page)

The Ouija algorithm[51] allowed us to retrospectively confirm the accuracy of the unsupervised pseudotime and to infer from a set of genes how cTEC fate was acquired. Interestingly, cTEC fate was determined by coordinated upregulation of functional cortical genes that remained stably expressed in the mature cortex as shown by the pseudotime heatmap (Figure 2E), which was further supported by selected single-gene plots, e.g., *PSMB11*, *PRSS16*, and *CTSV* (Figure 2F). Concomitantly, PolyKRT genes such as *CLU* were progressively downregulated and others, e.g., *ATF3* and *CCL5*, were transiently expressed in the transition cluster and then downregulated when the cells acquired cTEC identity (Figure 2F). Thus, acquisition of cTEC fate appeared to be determined by synchronous activation of cortical TFs and marker genes (e.g., *KCNIP3*, *SCX*, *PSMB11*, *CTSV*, and *PRSS16*) that established both differentiation commitment and traits associated with cortical function.

Viewed collectively, our data provide a first line of support for the hypothesis that the PolyKRT epithelial population comprises multipotent SCs of the postnatal thymus that are distinct from cortical and medullary intermediate progenitors.

Subcapsular and perivascular spaces are niches for PolyKRT cells

We next sought to identify the localization of PolyKRT cells within the complex 3D architecture of the human thymus. Because the expression of ECM-related genes was one of the identifying traits of PolyKRT cells, we used the expression of ECM-related transcripts and their protein products as markers of PolyKRT cells.

The ECM and, in particular, the basal laminae represent important components of epithelial SC niches in several tissues.[12] We therefore performed 3D reconstruction of confocal images of 300 μm thick sections for FN1, which is one of the main ECM components (also expressed by PolyKRT cells) to define the spatial distribution of the basal laminae of the postnatal human thymus. FN1 immunostaining clearly defined the subcapsular (SCap) and the perivascular spaces within the cortex and medulla; of note, the 3D reconstruction revealed that vascular structures were strikingly more abundant and larger in the medulla (Figures 3A, S3A, and S3B)

Next, we took advantage of GeoMx Digital Spatial Profiling (DSP) that allows whole-transcriptomics expression profiling of selected, immunostained regions of paraffin-embedded tissue sections.[52] This technology represents an unbiased solution to profiling anatomical regions by spatial resolution based on morphological markers. In 2D thin (5 μm) sections, it is challenging to define 3D vascular structures that in the medulla are organized in a dense, twisted net as shown by FN1 staining (Figures 3A and 3B). Therefore, guided by the IHC (above), we identified FN1-enriched medullary regions and SCap spaces as candidate areas to be enriched for PolyKRT transcripts. The challenge of this approach for the thymus tissue resides in the very high density of thymocytes intercalated within a network of TECs with branching morphology that are also relatively underrepresented. Anti-KRT8/18 immunostaining-defined epithelial cells and CD3/CD45 antibodies were used to maximize exclusion of the highly dense nuclei of thymocytes in each of these areas (segmentation). We also segmented regions of the central cortex (CC) as well as HB regions enriched for specialized cortical and medullary epithelial cells, respectively. SCap, CC, FN1-medulla (FNm), and HB were segmented for KRT8/18-positive and CD3/CD45-negative as regions of interest (ROIs) (Figure 3C). Four ROIs for each tissue section of three biological samples were then processed for sequencing (Figure 3C). The dimensional reduction analysis (principal-component analysis [PCA] plot) clearly illustrates how the selected ROIs clustered accordingly, evidently profiling functionally distinct regions (Figure 3D). Strikingly, the PolyKRT signature (including *FN1*, *BCAM*, *LIFR*, and *IFITM3*) was enriched in both SCap and FNm but neither in the CC nor in the HB, which were instead defined by their specialized signatures (Figure 3D).

The BCAM emerged in the scRNA-seq dataset as well as in GeoMx DSP as one of the ECM-related genes defining the PolyKRT signature (Figures 1C and 3E). BCAM is the receptor of laminin-A5 (LAMA5), an ECM glycoprotein of the basal laminae,[53,54] and its expression was detected together with other PolyKRT-specific proteins (FN1, IFITM3, TIMP1, and KRTs) in SCap spaces and perivascular regions (Figures 3E

(B) Immunofluorescence labeling of thymic epithelial cells co-stained with anti-ITGA6 (CD49f) antibody (magenta), FN1 (green), and EpCAM (gray). Left: co-staining in the subcapsular region; right: co-staining in the medullary area. Asterisks (*) indicate areas of colocalization. Nuclei counterstained with DAPI. Scale bars, 50 μm (n = 4, human thymi).

(C) Top: representative immunofluorescence image of an entire human thymus paraffin section (5 μm) analyzed for spatial transcriptomics (n = 4). FN1 staining is shown in green, CD45 and CD3 in red, while KRT18 areas in cyan. Nuclei counterstained with SYTO85 dye. Regions of interest (ROIs) localization is displayed and named in white for each type. Scale bars, 5 mm. Bottom: representative image of each ROI segmented for KRT18-positive cells is displayed in white and labeled as follows: HB (Hassall's body), FN1 medulla (FNm), central cortex (CC), and subcapsular (SCap).

(D) Dimension reduction analysis displays transcriptional variation of ROIs along PCA2 versus PCA3. The heatmap graph shows the gene expression of PolyKRT and specialized cortical and medullary signature per each ROI, HB (dark red), FNm (red), CC (dark green), and SCap (green). Scale $\log_{10}$ (−3,3).

(E) UMAP feature view plot of *BCAM* showing the expression in the PolyKRT cluster.

(F) Representative immunofluorescence of a human thymus entire paraffin section (5 mm) analyzed for multiplex multispectral imaging (AKOYA): KRT15 (white), BCAM (green), and TP63 (red). Nuclei counterstained with DAPI. Scale bars, 1 mm (n = 6, human thymi).

(G) Higher magnification of subcapsular and medullary areas show rare cells with triple colocalization of KRT15, BCAM, and TP63. Scale bars, 50 μm. BCAM-single-positive endothelium is marked by asterisks (*). Third panel of each area highlights $BCAM^{pos}TP63^{pos}$ single cells in the subcapsular (left) and medullary areas (right). Scale bars, 20 μm.

(H) Triple-positive ($KRT15^{pos}$, $BCAM^{pos}$, and $TP63^{pos}$) single cells have been quantified as both absolute number per annotated area and percentage of total epithelial cells ($TP63^{pos}$) in each cortical and medullary area (10–15 cortical and medullary annotated areas, average annotation size 0.29 mm^2, n= 6 human thymi). Medulla shows a higher number of triple-positive cells than cortex. Significance: Mann-Whitney test, non-parametric; ****$p < 0.0001$.

(I) Triple-positive cells were quantified as both absolute number per annotated area and percentage of total epithelial cells ($TP63^{pos}$) in CC and SCap areas. SCap areas are enriched for triple-positive cells. Significance: Mann-Whitney test, non-parametric; ****$p < 0.0001$.

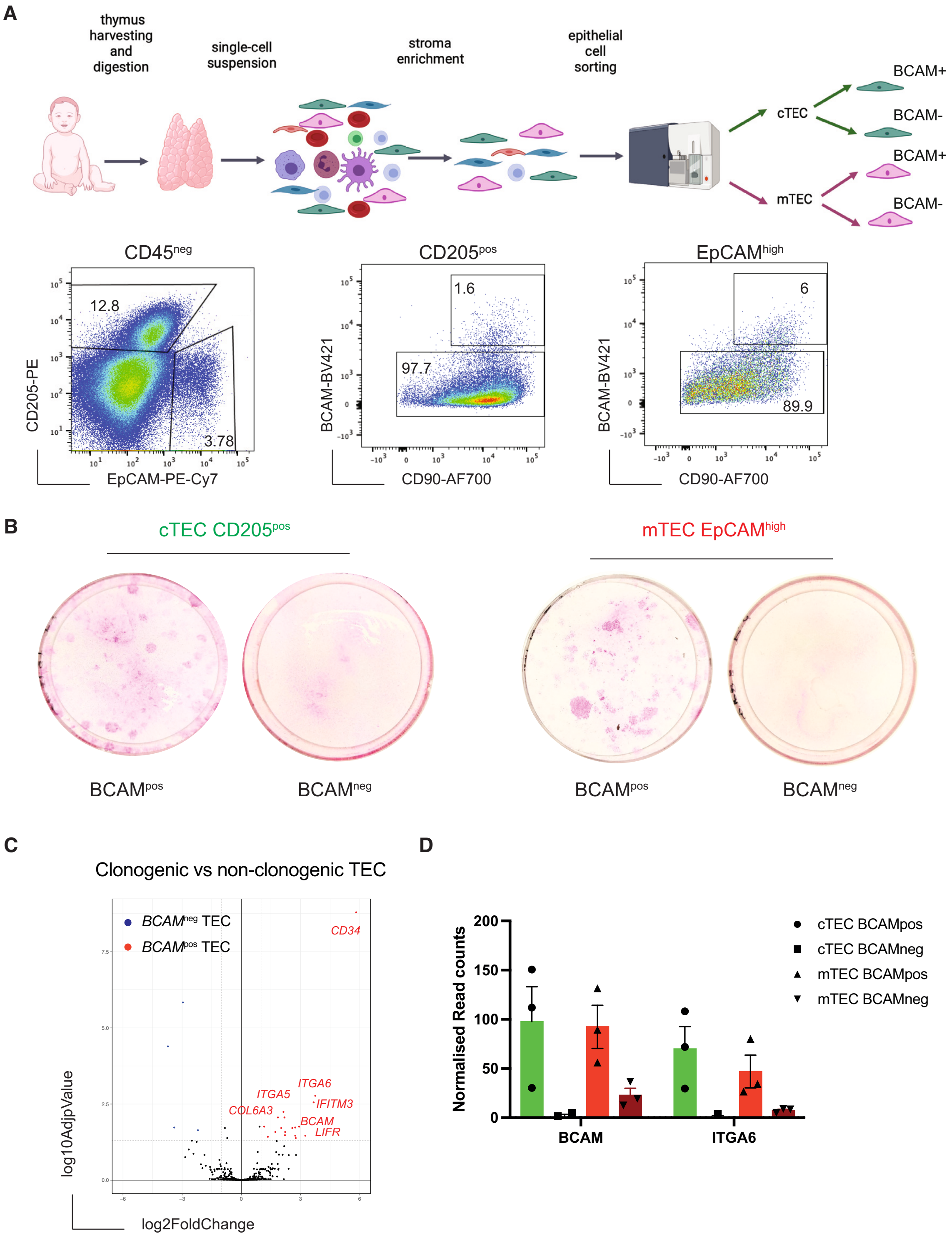

Figure 4. Prospective isolation of mTEC and cTEC PolyKRT

(A) Workflow of thymic tissue digestion, stromal cell enrichment, and fluorescence-activated cell sorting (FACS) for cortical and medullary epithelial populations. Image created with Biorender.com. Representative FACS plots of dissociated and enriched thymic cells from human postnatal thymi (n = 13). cTECs and mTECs were gated for BCAM expression. Four TEC populations were sorted.

(B) Rhodamine-B staining of sorted cTEC and mTEC populations after two passages in culture demonstrating high clonogenic potential of BCAM^{pos} cells that gave rise to colonies of variable sizes that stained either strongly or dimly with rhodamine-B (n = 4, donor-derived cultures).

(legend continued on next page)

and S3C–S3G). We next investigated the pattern of expression of the TP63 TF and of its isoform ΔNTP63α that is expressed in the basal layer of the epidermis and that was previously associated with stemness in various epithelia, including that of the thymus.[13,15,55] We found that TP63 antibody (recognizing all TP63 isoforms) detected a very broad expression pattern in the thymus epithelium, with some brightly stained cells in both the cortex and medulla that colocalized with KRT17 and KRT18 (Figure S3H). Conversely, the ΔNTP63α isoform displayed a more restricted expression pattern, largely reflecting that of BCAM-expressing epithelial cells (Figure S3I).

Therefore, we used the single-cell spatial phenotyping technology by Akoya Biosciences to confirm PolyKRT cells across whole-tissue sections of thymi aged from 3 months to 10 years. Using the PhenoImagerHT multispectral system, we validated the co-expression of the marker gene BCAM with KRT15 and TP63 (all isoforms).[56] PolyKRT cells were detected in both cortical and medullary compartments as a fraction of TP63-positive nuclei (Figures 3F and 3G). Single cells positive for BCAM, KRT15, and TP63 were detected in the areas where the basal laminae are more strongly represented, thus demonstrating a higher proportion of BCAM^{+} in the medulla compared with the cortex (Figures 3H and 3I).

In sum, the combined use of spatial transcriptomics and immunophenotyping allowed us to define the niches of PolyKRT SC as the SCap and perivascular regions.

PolyKRT cells are the clonogenic SCs of human thymus

Clonogenicity is a feature defining epithelial SCs in several tissues and was first established for skin keratinocyte SCs.[6,16] Our previous data indicated that thymic clonogenic cells were included in the mTEC and cTEC populations that are CD49f^{pos} (ITGA6).[35] We therefore performed scRNA-seq of the CD49f^{pos}-sorted population and found that PolyKRT cluster segregated in this sorted population along with other specialized TEC clusters (Figures S4A and S4B). In this dataset, we observed that PolyKRT cells were negative for CD24, a surface marker expressed by specialized mTECs and for CD83, a marker expressed by specialized cTECs (Figure S4B). CD90 was previously described as expressed by TECs *in vivo* and *in vitro*.[35,57] When we validated the CD24 protein expression pattern in the CD49f^{pos} population using flow cytometry, we also observed that the CD24^{neg} population was CD49f^{high} and CD90^{high} as shown by mean fluorescence intensity (MFI) quantification (Figure S4C). Indeed, when cortical (CD205^{pos}EpCAM^{low}) and medullary (CD205^{neg}EpCAM^{high}) cells were further subdivided based on the level of expression of CD49f, CD90, and CD24 (Figure S4C) and assessed independently for clonogenicity in culture, only CD49f^{high}CD90^{high}CD24^{neg} cells gave rise to expanding colonies, irrespective of being derived from either the medulla or the cortex (Figures S4D and S4E).

To confirm whether clonogenicity trait was displayed by the PolyKRT cells, we endeavored to purify them based on the level of expression of the just validated marker, BCAM. cTECs and mTECs were further subdivided into BCAM^{pos} and BCAM^{neg} TEC; all four cell fractions were sorted and plated to test their clonogenic capacities (Figures 4A and S4F). Following flow cytometry sorting, we found that only BCAM^{pos} cells in both cortex and medulla were clonogenic and gave rise to epithelial colonies that could be extensively sub-cultured and expanded *in vitro* (Figures 4B and S4G).

Freshly isolated clonogenic (BCAM^{pos}) and non-clonogenic (BCAM^{neg}) TECs were subjected to nCounter automated analysis (NanoString) that allows multiplex gene expression profiling. Of note, clonogenic BCAM^{pos} cells when freshly isolated, retained some of their cortical or medullary identity reflected by the differential expression of compartment-specific genes such as cortical *CTSV, FOXN1, PSMB11*, *SCX*, and *LY75* and medullary *CCL21*, *EpCAM*, *CLDN3*, and *CLDN4*, respectively (Figure S4H). Furthermore, they retained the expression of keratins (*KRT5, KRT13, KRT14, KRT17,* and *KRT18*) typical of PolyKRT cells (Figure S4I). Of note, the volcano plot displaying the differentially expressed genes (DEGs) between clonogenic and non-clonogenic TECs provided an independent confirmation, using an independent method, of PolyKRT gene expression in the clonogenic fractions, together with *BCAM* (Figures 4C and 4D).

It has been observed that once isolated from their niche *in vivo* and challenged for *in vitro* expansion under defined conditions, SCs activate and adapt to the culture microenvironment as reflected in modified gene expression profiles.[58,59] To investigate whether clonogenic SCs retained cortical and medullary identities, respectively, together with PolyKRT traits in culture, we performed scRNA-seq of cTEC and mTEC expansion *in vitro*. Such analysis also allowed us to address whether clonogenic cells expanding in culture would differ depending on their compartment of origin (cortex or medulla) and/or method of isolation. Cells were expanded for several passages and sub-confluent (i.e., still expanding) cultures were harvested and processed for 10× transcriptomic single-cell sequencing (Figure 5A). For comparison with cells of known long-term regenerative potential, we also included a culture of epidermis-derived clonogenic SCs, i.e., skin keratinocytes.[16] All samples were further processed for sub-clustering to eliminate contaminating mouse feeder cells (Figure S5A).

All thymic cultures, independent of their derivation, showed comparable profiles in the UMAP plots with identification of three main cell groups i.e., cluster 1 (C1), C2, and C3 (Figure 5B). Of note, none of the cultures expressed cortex- or medulla-specific transcripts, e.g., *LY75*, *PRSS16*, *FOXN1*, *CD74*, *ASCL1*, *CLDN3*, *CCL21*, *SOX2*, and *MYOG* (Figure S5B). Thus, the transcriptional memory of the cortical or medullary origin was essentially silenced when clonogenic TECs were seeded and expanded in 2D culture (Figures 5B and S5C).

When we compared scRNA-seq datasets of SCs growing *in vitro* and the PolyKRT cluster *in vivo*, the degree of transcriptional overlap was striking, with the PolyKRT signature equally

(C) Volcano plot analysis of freshly sorted clonogenic versus non-clonogenic thymic epithelial cells. All genes present on the StemCell NanoString nCounter Panel-Plus have been plotted. Each dot represents one gene. A p value of 0.05 and a fold change of 2 are indicated by gray, highlighting the most significantly upregulated (red) and downregulated (blue) genes (n = 3, thymic samples).
(D) Single-gene expression profiles of adhesion molecules and surface markers expressed by BCAM^{pos} and BCAM^{neg} TECs via nCounter NanoString Technologies (n= 3, thymic samples).

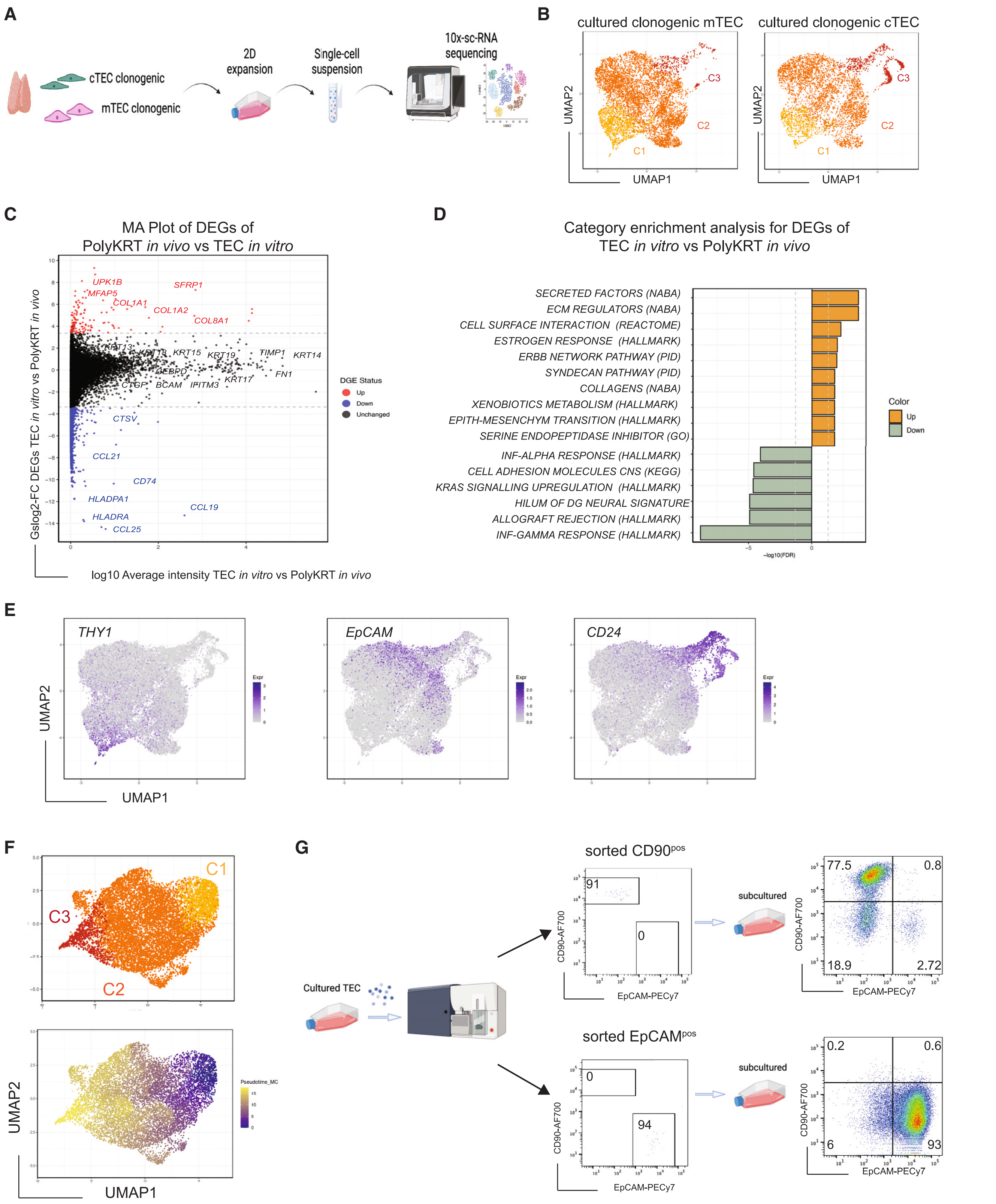

Figure 5. Single-cell RNA-seq analysis defines a thymus-specific cell signature *in vitro*

(A) Workflow of cell preparation for single-cell transcriptome profiling of cultivated human cTEC and mTEC cultures. Image created with Biorender.com.

(B) UMAP plot visualization of cultured mTECs and cTECs colored by cell cluster group per each representative sample (n = 2, donor-derived cultures of mTECs and cTECs).

(legend continued on next page)

expressed in both conditions, as shown by key signature genes lying along the middle line in the MA plot (Figure 5C). This integration analysis also highlighted a few differences between these populations, driven by their two highly distinct environments. *In vitro*-expanding SCs unsurprisingly activated genes associated with proliferation, cell motility, and ECM-regulator pathways, e.g., *COL1A1, COL1A2*, *UPK1B*, *MFAP5*, and *SFRP1* (red data points), while *in vivo* PolyKRT were characterized by the expression of chemokines and receptors, e.g., *CCL19, CCL21, CCL25,* and *CD74* (blue data points), most likely facilitating the crosstalk with thymocytes and other components of their niche that are missing *in vitro* (Figures 5C and 5D). Thus, PolyKRT are the clonogenic SCs of human thymus and retain their signature while expanding in the *in vitro* microenvironment.

Clonogenic PolyKRT SCs display thymus-specific traits in culture

Clustering analysis showed that *in vitro*-expanding PolyKRT and epidermal cells shared C2 and C3 clusters, although cluster C1 emerged as thymus specific, strikingly illustrating its atypical epithelial signature, i.e., *FN1*, *TIMP1*, *IFITM3*, and *VCAM1* in addition to *CD90* (*THY1*) (Figures 5E and S5D–S5F). The C1 cluster was further confirmed to be a distinctive, thymus-specific feature in another dataset, which also included a culture of airway basal cells (Figure S5G). By contrast, clusters C2 and C3 were common to TEC, epidermal keratinocyte, and airway cell cultures and included established markers of stratified and cornified epithelia (Figure S5H).

Finally, using single-cell trajectory analysis (Monocle pseudotime), we investigated whether TEC clusters were hierarchically organized. Indeed, the thymus-specific cluster C1 appeared to give rise to all the TEC types (C2 and C3) (Figure 5F; Table S1). Therefore, using flow cytometry, we analyzed thymic cultures stained for the surface markers that were differentially expressed by each cluster (i.e., CD90, EpCAM, and CD24), excluding mouse 3T3-J2 feeder cells by staining with a Feeder-PE antibody; thus, cultured TECs were identified as CD49f^{pos}Feeder-PE^{neg} (Figure S6A). In line with transcriptomic data, most cultured TECs did not express EpCAM, whereas a proportion of EpCAM^{neg} cells was positive for CD90, consistent with the C1 cluster signature. In contrast, CD24 was only expressed by a subpopulation of EpCAM^{pos} cells as reflected in the C2 and C3 clusters, whereas skin keratinocytes and airway cultures co-expressed EpCAM and CD24 but were all CD90^{neg} (Figures S6B and S6C). Enhanced heterogeneity of TEC cultures was also evident from staining for rhodamine-B, a dye that is used to evaluate epidermal cell keratinization that is a correlate of its intensity in cultured cells.[60] Whereas some TEC colonies stained strongly with rhodamine, others had a dim stain, which was in stark contrast with the homogeneous, dark-rhodamine colony morphology displayed by skin and airway cell colonies (Figures S6A–S6C).

To witness how the heterogeneity of TECs in culture is established, we flow sorted to high purity expanding clonogenic TECs based on their specific surface markers CD90^{pos}EpCAM^{neg} and CD90^{neg}EpCAM^{pos} (Figures 5G and S6D). We then assessed the phenotypic profile of these sorted populations by flow cytometry upon culture and serial passages. After only one passage, sorted CD90^{pos} cells gave rise to all populations of the original culture including CD90^{pos}EpCAM^{neg}, CD90^{neg}EpCAM^{neg}, and CD90^{neg}EpCAM^{pos} TEC. On the contrary, CD90^{neg}EpCAM^{pos} maintained their phenotype, although they also generated a small proportion of CD90^{neg}EpCAM^{neg} cells, thus confirming the capacity of TECs to downregulate EpCAM protein in culture (Figure 5G).

In summary, the experiments have further strengthened the case for PolyKRT being the human postnatal clonogenic SCs showing the notable trait of colony heterogeneity and unconventional epithelial traits.

Distinct morphological traits of clones *in vitro* define SC properties

To investigate whether the nature of thymic culture heterogeneity may reflect a differential self-renewal capacity of clonogenic PolyKRT SCs, we performed single-cell clonal analysis (Figure 6A). Such analysis has previously established the functional heterogeneity of *ex vivo* human keratinocyte SCs that permanently repair and sustain human epidermal grafts as life-saving therapies for large burns and congenital genodermatosis.[6,61] Once a keratinocyte clone is expanded, its capacity to sustain growth (self-renewal) can be evaluated from plating a fraction of its cells in an indicator dish (i.e., definition of holoclone, meroclone, and paraclone).[6] Furthermore, this approach enabled us to classify TECs based on colony diversity that had emerged (above) as a thymus-specific trait (Figures S7A–S7C). Phase-contrast images of individual colonies highlighted a morphological heterogeneity, permitting us to define the following: (1) "refractive-edges" colonies that were composed of cells with refractive borders under phase-contrast imaging and (2) "stratified" morphology for its similarity to the colony morphology of cultured stratified epithelia, e.g., epidermal keratinocytes (Figure 6B). Colonies that displayed a piled-up differentiation phenotype were named "aborted" as a reference to the aborted keratinocyte colonies, whereas cells that were highly mobile were called "scattered" (Figure S7A).

To evaluate which type(s) of colony was expressing the thymus-specific signature identified by scRNA-seq, i.e., the C1

(C) MA plot of differentially expressed genes of *in vitro* thymic SCs versus *in vivo* PolyKRT cluster. The $\log_2$ fold change indicates the mean expression level for each gene. Each dot represents one gene.
(D) Category enrichment analysis for differentially expressed genes between *in vitro* thymic SCs and *in vivo* PolyKRT cluster, showing the most significantly upregulated and downregulated pathways. Hypergeometric test was performed on the top upregulated and downregulated genes to identify overrepresented gene categories.
(E) UMAP plot visualization ($\log_{10}$ expression) of surface marker genes across clusters indicates heterogeneous transcriptional profiles in thymic SCs upregulating *THY1* (C1), *EpCAM*, and *CD24* (C2 and C3) markers.
(F) Top: UMAP plot visualization of TEC cultures colored by the cell cluster group shows three main clusters C1 (yellow)-C2 (orange)-C3 (dark red). Bottom: UMAP plot graph colored according to Monocle pseudotime: cells in C1 transition to C2 and C3 (n = 8, thymus-derived cultures).
(G) Representative FACS analysis of sorted and sub-cultured CD90^{pos} and EpCAM^{pos} populations. Schematic on the left; purity check of sorted populations, center; and the analysis of the phenotype of sorted populations, right (n= 5, donor-derived cultures).

CellPress
OPEN ACCESS

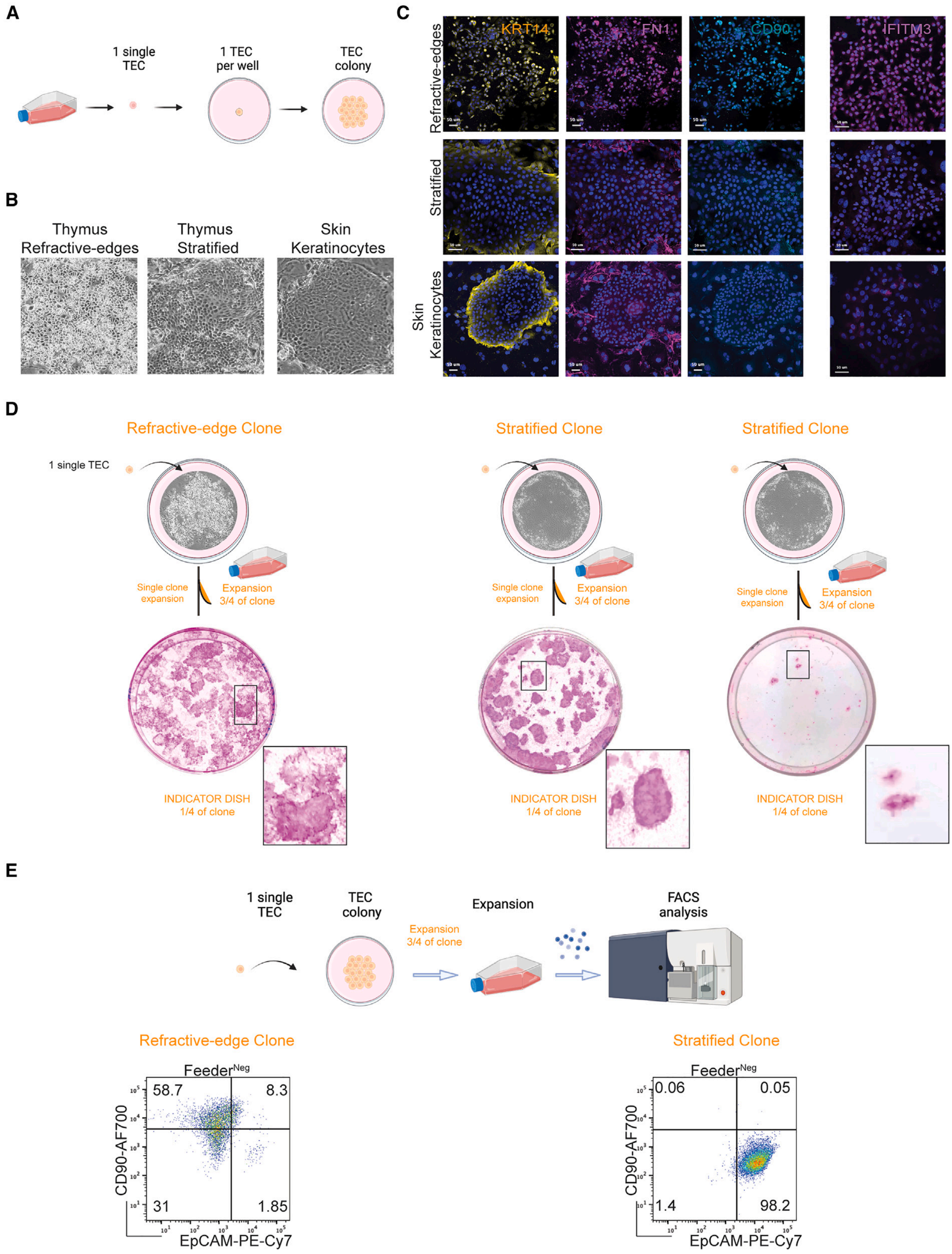

(legend on next page)

cluster, we stained the different clonal colonies for KRT14 and the identified proteins such as FN1, IFITM3, TIMP1, and CD90. We found the expression of these proteins only in refractive-edges morphologies and not in the stratified colonies derived from either thymus or epidermis (Figures 6C and S7B).

Next, we expanded single clones to study the growth potential and the hierarchical relationship of each morphological cell type. After 1 week of clonal culture, each clone was trypsinized and three-quarters of each clonal colony was replated for further expansion, whereas one-quarter was seeded in an indicator dish fixed and stained for rhodamine-B after 12 days to examine growth potency and morphology. The dim rhodamine-B-stained colonies corresponded to refractive-edges morphology, while stratified clones were strongly stained with rhodamine-B. These results confirmed that single cells with refractive-edges colony-forming capacity were able to generate all morphology types upon subculture, while stratified produced only stratified and/or terminally differentiated (aborted) colonies (Figures 6D and S7C). When we analyzed the profile of the progeny of expanded single clones by flow cytometry, we observed that refractive-edges clones gave rise to distinctive $CD90^{pos}EpCAM^{neg}$, $CD90^{neg}EpCAM^{neg}$, and $CD90^{neg}EpCAM^{pos}$ subpopulations, while stratified clones gave rise only to $CD90^{neg}EpCAM^{pos}$ population (Figure 6E). Of note, the refractive-edges colonies could be expanded with an average efficiency of 72.9% (with a peak of 81%), while stratified clones had a lower efficiency (20.8%, with a peak of 33%) and gave rise to aborted colonies, reminiscent of meroclones and paraclones (Figures S7D and S7E). Thus, the properties of refractive-edges cells were consistent with those expected of a clonal thymic SC population in culture that self-renews while giving rise to progenitors with more limited expansion potency.

Clonogenic SCs retain PolyKRT multilineage differentiation potency

Our bioinformatic-based analyses of the scRNA-seq datasets, described at the start of this study, indicated that PolyKRT cells harbored multilineage differentiation potential *in vivo*. To determine if *in vitro* expanded SCs retained multilineage differentiation potency of PolyKRT, we developed an assay that favored the differentiation of clonogenic SCs obtained by seeding only one expanded epithelial cell type, with no support from other stromal or hematopoietic cells. Differentiated cells were then either fixed for whole-mount IHC or lysate processed for real-time quantitative PCR analysis (Figure 7A). Cortical differentiation, marked by LY75 and null for KRT5/14, and medullary differentiation, marked by KRT5/14 and null for LY75, were achieved independently from the compartment of origin of the cells (clonogenic cTECs or mTECs) that initiated the culture (Figure 7A). Of note, differentiating cells were able to generate and organize mTEC-HB-like regions ($KRT10^{+}$), sparse Io ($KRT7^{+}$), medullary progenitors ($ASCL1^{+}$), and myoid cells (DES^{+}) that evoked medullary areas of the native thymus (Figures 7B–7D), whereas the same markers were not expressed by clonogenic SCs in expansion (Figures S8A–S8D). Medullary fates were further confirmed by the upregulation of mTEC transient progenitor cluster genes (*ASCL1*, *CLDN3*, and *CLDN4*), myoid (*MYOG*), neuroendocrine, and mechanosensory (*SOX11*, *SOX2*, and *SYP*) cell lineage genes, whereas a cortical fate was evidenced by the upregulation of *LY75*, *KCNIP3*, *CD74*, *CTSV*, *CD274*, and *FOXN1* genes in differentiated cultures, compared with counterpart cells undergoing expansion (Figure S8E). To conclusively demonstrate the intrinsic multipotency of thymic SCs, we expanded single clones and assessed their progenies by the same assay described above. All the clones capable of expansion growth demonstrated multilineage differentiation potency, giving rise to multiple medullary and cortical fates (Figure S9A).

Finally, we assessed whether multilineage differentiation of clonogenic SCs would also be achieved *in vivo* using a whole-organ thymus reconstitution assay that we had previously developed.[35] Thymus reconstitution by expanded SCs was characterized by stroma compartmentalized into cortical and medullary regions including HB formation. Thymus reconstitution was characterized by the progressive maturation of the stroma and its compartmentalization into cortical and medullary regions, while seeding of hematopoietic progenitors followed by thymocyte ($CD3^{+}$cells) development demonstrated the appropriate functioning of the reconstituted organs (Figures 7E, S9B, and S9C). Strikingly, the reconstituted tissues displayed cortical and medullary epithelial subtypes derived from expanded clonogenic SCs with HB formation ($KRT10^{+}$), medullary progenitors ($ASCL1^{+}$), and neuroendocrine cells ($SOX2^{+}$), which reflected the spatial phenotype of the native thymus (Figures 7F and 7G).

Thus, we could conclude that similarly to PolyKRT *in vivo*, clonogenic SCs retained multipotency after isolation and *in vitro* expansion, with the capacity to reconstitute multiple thymic compartments even from a single clone. These are akin to the defining criteria for human multipotent SCs, which include the proven capability of self-organization and organ reconstitution.

DISCUSSION

Our data collectively identify and characterize *bona fide* epithelial SCs with multilineage differentiation potency in the human

Figure 6. Single-cell cloning of thymic SCs *in vitro*

(A) Schematic showing single-cell cloning of cultured thymic SCs, created with Biorender.com.

(B) Phase-contrast images of individual thymic colonies classified according to their cell morphology and colony pattern as follows: refractive-edges and stratified. Keratinocytes are classified only as stratified colonies.

(C) Immunofluorescence staining for CD90 (THY1), IFITM3, and FN1 of expanding thymic epithelial ($KRT14^{pos}$, yellow) colonies. IFITM3 or FN1 (magenta) and CD90 (THY1, cyan) were expressed by refractive-edges, but not by stratified or keratinocyte colonies; FN1 was detected also in mouse feeder cells. n= 4, donor-derived cultures. Scale bars, 50 μm.

(D) Single-cell clonal expansion of refractive-edges and stratified clones. Refractive-edges clones gave rise to colonies of different levels of rhodamine-B staining; stratified clones display colonies with high intensity of rhodamine-B staining and gave rise to both stratified and aborted colonies. Bottom: high magnification of colonies displaying strong versus dim rhodamine-B staining. n= 5 independent cultures.

(E) Schematic showing FACS analysis after clone expansion. Representative FACS analysis showing CD90 and EpCAM profile in expanded clones: refractive-edges gave rise to $CD90^{pos}$, $CD90^{neg}$, and $EpCAM^{pos}$ cells, while stratified colonies only to $EpCAM^{pos}$ cells (n = 7 refractive-edges and n = 4 stratified clones).

CellPress
OPEN ACCESS

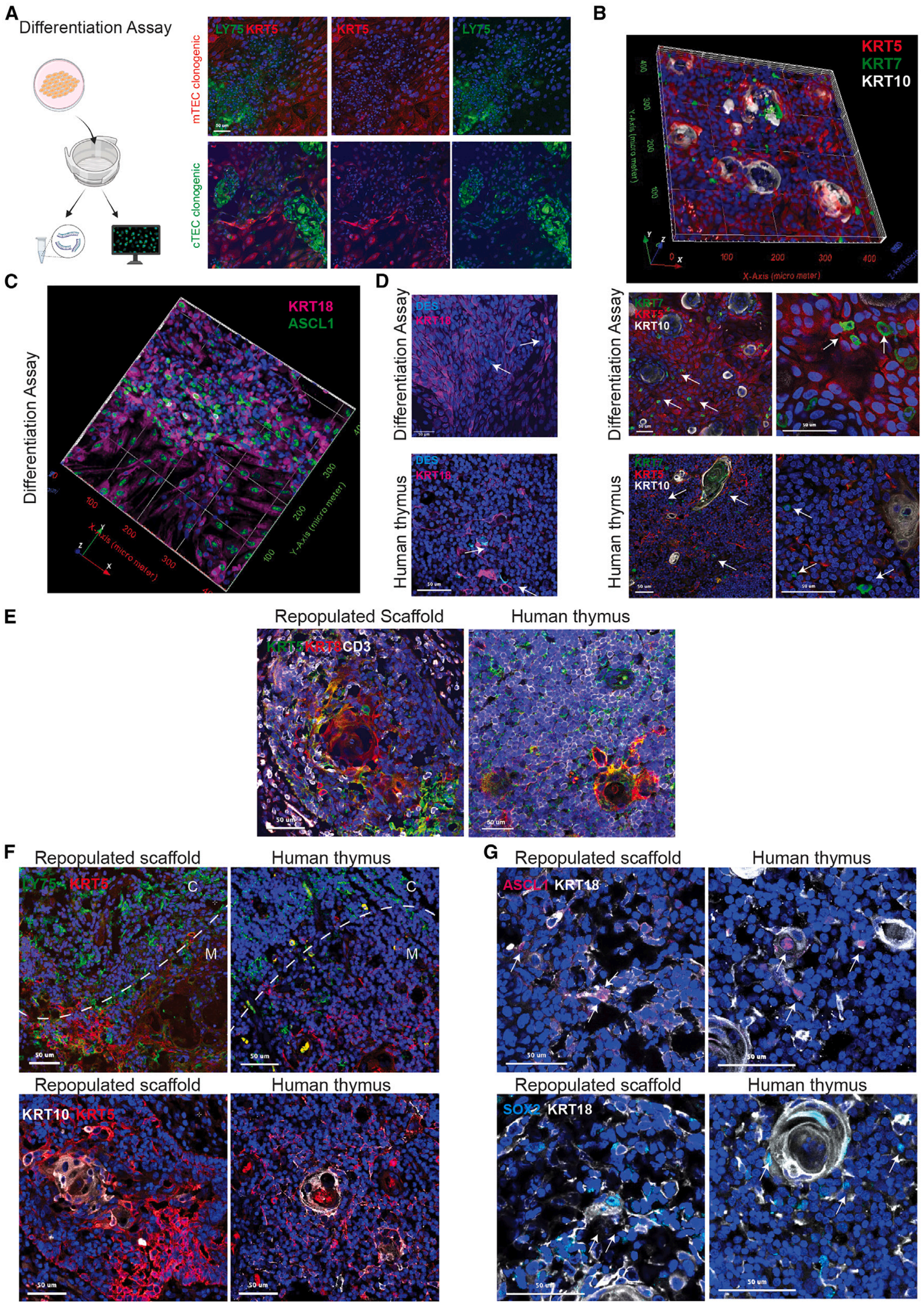

(legend on next page)

postnatal thymus. Thymic SCs have some unanticipated traits and are defined by gene expression signatures that reflect their capacity to sustain multiple functions and resist stress and insults of various types. Indeed, our data support a model whereby postnatal SCs can be isolated from both human cortical and medullary compartments based on common features and defined surface molecules. They have additional key properties, which contrasts them with the prior definition of "thymic progenitors".[62,63] Hitherto, putative "progenitors" were poorly characterized,[24,33–35] while we define both SCs and progenitor cells based upon specific signatures that were experimentally validated.

Thymic SCs give rise to cTECs including a functionally distinct cell cluster (cTEC-IEGs) that constitutively express PD-L1 and may play a role in controlling the activation of immature thymocytes; they also generate specialized medullary cell types, including myoid and neuroendocrine cells that were hitherto not assigned to the epithelial lineage and previously considered of uncertain origin.[33]

Although studied essentially as the site of T cell development, the thymus is an endocrine gland. It is also known that the immune system is functionally linked to neuroendocrine axes, constituting an integrated homeostatic network.[64] Here, we characterize two main subtypes of thymic cells with neuroendocrine features. The first cell type expresses TFs, e.g., *NEUROD1*, that are essential for endocrine cell development in other tissues such as the gut.[65] A second neuroendocrine cluster upregulates transcripts (e.g., *IRX2, SOX2*, and *ATOH1*) involved in the development of sensory/neuroendocrine cells of epithelial origin such as Merkel cells of the skin[66,67] or the inner ear hair cells.[68–70] These thymic sensory/neuroendocrine cells, as well as myoid and Io subtypes, derive from a common ASCL1-expressing epithelial progenitor. ASCL1 is critical for the ordered development of neuronal populations[71,72] and also of neuroendocrine cells in the lung.[73] Binding of ASCL1 often precedes the appearance of regions of open chromatin that are associated with *de novo* gene expression during neuronal differentiation.[74] Its function in transcription and chromatin landscape regulation might be critical also in the thymus. This trait seems germane to recent claims that the murine thymic medulla contains cells that mimic peripheral tissue antigen presentation for tolerance induction and that are defined by lineage-specific TFs.[24] Thus, our finding of thymic epithelial SCs with pleiotropic multilineage differentiation may indicate that the medullary cells are *bona fide* diverse cell types and may represent a key to understanding mechanisms shaping the medullary tolerogenic microenvironment. Additionally, they may determine the high heterogeneity of thymic tumors (i.e., thymoma and thymic carcinoma), including the most aggressive rhabdomyosarcoma and primary neuroendocrine tumors of the thymus (NETTs), often associated with autoimmunity.[75]

Once isolated from healthy tissue, thymic SCs are clonogenic and can be extensively expanded, fulfilling the key property of SCs of constantly renewing tissues such as the epidermis. This seems paradoxical, given that the epidermis is characterized by high cell turnover and is entirely replaced every 3 weeks lifelong, whereas the thymic epithelium is not actively proliferating, and the thymus itself involutes with progressively decreasing functional outputs throughout postnatal life. Nevertheless, we isolated clonogenic SCs from pediatric to young adult age groups when involution mechanisms are already active. Of note, thymic SCs display the striking capacity to phenocopy cortical and medullary compartments *ex vivo* without developmental stromal instructive signals, thus fulfilling the property of somatic SCs.

Additionally, the thymic SCs display some distinctive traits that provide significant insights into the broader field of epithelial SC biology. First, thymic multipotent SCs express ECM-binding proteins, such as BCAM, that are important for polarization and adherence to the basal laminae[54,76] and contribute to their niche by producing ECM proteins, e.g., FN1. In the thymus, the basal laminae are distributed along a complex 3D architecture, with the SCap spaces and the perivascular regions to be the areas where PolyKRT SCs reside. Of note, our findings define their localization to a much broader area than the cortical-medullary junction (CMJ) where thymic progenitors had been hypothesized to reside.[77] The importance of epithelial adhesion to specific ECM for stemness maintenance is further demonstrated by the capacity of proteins such as FN1 to inhibit human keratinocyte terminal differentiation.[78] Reflecting the abundance and distribution of the basal laminae, thymic SCs reside both in the cortical and medullary compartments but are more abundant in the medulla. Our 3D imaging data also unraveled a striking, hitherto unreported difference of blood vessel density between the two compartments.

Second, thymic epithelial SCs constitutively express several genes involved in immune and inflammatory responses. For

Figure 7. Thymic SCs retain the ability to differentiate in multiple fates *in vitro* and *in vivo*

(A) Schematic workflow for thymic SC differentiation assay and downstream analysis, created with Biorender.com. Immunofluorescence staining of differentiated cTECs and mTECs show differentiation potency toward both cortex (LY75, green) and medulla (KRT5, red); n = 5, TEC cultures. Nuclei counterstained with DAPI. Scale bars, 50 μm.

(B) Immunofluorescence z stack composite of differentiated TECs: cells positive for KRT5 (red), scattered ionocytes (KRT7 positive in green), and areas with Hassall's bodies (HBs) KRT10-positive structures (white). Immunofluorescence of differentiated TECs show maturation into KRT5 (red), KRT7 (green, indicated by white arrows), and/or KRT10 (white) cells, as in the native human thymus (low and high magnification, n= 5 differentiation assays and n = 3 donor thymi). Nuclei counterstained with DAPI; scale bars, 50 μm.

(C) Immunofluorescence z stack of differentiated thymic SCs shows ASCL1 expression (green) in KRT18-positive cells (magenta) (n = 4, differentiated cultures).

(D) Immunostaining of TECs upon differentiation assay indicates myoid KRT18-positive DES$^+$ cells (white arrows), with pattern similar to human postnatal thymus. Nuclei counterstained with DAPI. Scale bars, 50 μm (n = 3, differentiated cultures).

(E) Immunofluorescence of thymic scaffold graft sections (7 μm) at 16 weeks post transplantation and human thymus stained against human KRT5 (green), KRT8-18 (red), and CD3 (gray). Nuclei counterstained with DAPI. Scale bars, 50 μm (n = 8, repopulated scaffolds per time point).

(F) Immunofluorescence of thymic scaffold grafts (16 weeks post transplantation [wpt]): sections (7 μm) stained for cortical (LY75, green), medullary (KRT5, red), and HB regions (KRT10, gray). Scale bars, 50 μm.

(G) Immunofluorescence of 16 wpt graft sections: ASCL1 (magenta) and KRT18 (gray) for medullary transition cells and KRT18 (gray), SOX2 (cyan) for neuroendocrine/mechanosensory cells. Nuclei counterstained with DAPI. Scale bars, 50 μm (n = 8, repopulated scaffolds per time point).

CellPress
OPEN ACCESS

example, IFITM3 likely represents an increased resistance of these cells to viral infections, a property that has been observed in other SCs.[37] Indeed, its constitutive expression in unstimulated epithelial SCs is striking when compared with its strictly regulated expression in infected or inflamed tissues or in non-epithelial stromal cells.[79–81] Nonetheless, we note that *in vivo*, the thymus is a site of constitutive type I interferon (IFN) α expression[82] that may contribute to IFITM3 regulation.

Third, thymic SCs co-express several KRTs that are typically known for their specificity and lineage restriction.[41,42] Thus, thymic SCs display a PolyKRT signature, not described so far among any other epithelia. This might underpin the plasticity of epithelial SCs—the capacity to increase their potency during differentiation—that has been reported for TECs in the context of transplantation.[11,83]

These data have important implications for future cell replacement therapies (i.e., transplantation in athymic patients) and may facilitate translational uses similar to epidermal and limbal SCs that are currently in clinical use.[61,84] Thus, by studying how thymic SCs and their niches adapt during the progressive atrophy of thymus and how they respond to exogenous factors both *in vivo* and *in vitro*, we shall be able to study the mechanisms of thymic involution and design future strategies for increasing thymic output, e.g., to augment vaccination responses in vulnerable subjects or improve the immune response against cancer.

Limitations of the study

Further studies will be required to dissect the specific role of PolyKRT signature genes, for example, via genetic manipulation. The challenge of this approach would be to manipulate clonogenic SCs without affecting their self-renewal capacity in culture prior to differentiation assays.

STAR★METHODS

Detailed methods are provided in the online version of this paper and include the following:

- KEY RESOURCES TABLE
- RESOURCE AVAILABILITY
  - Lead contact
  - Materials availability
  - Data and code availability
- EXPERIMENTAL MODEL AND STUDY PARTICIPANT DETAILS
  - Human tissues
  - Animal models
- METHOD DETAILS
  - Thymic epithelial cell (TEC) isolation and sorting
  - Epithelial stem cell culture (thymus, skin and airways cells)
  - Differentiation of thymic epithelial cultures
  - Flow cytometry analysis
  - *In vivo* assay: grafting thymic rat scaffolds into NSG-Nude mice
  - RNA isolation and real-time quantitative PCR
  - Single-cell RNA sequencing – analysis of thymic fresh tissue and cultivated cells
  - Bioinformatics data analysis of single-cell data
  - Differential gene expression and Trajectory analyses
  - Gene expression profile nCounter analysis
  - Histology
  - Immunostaining
  - 3D Immunostaining of human thymic tissue
  - Spatial profiling with GeoMx
  - AKOYA Multiplex Imaging analysis
  - Imaging
- QUANTIFICATION AND STATISTICAL ANALYSIS

SUPPLEMENTAL INFORMATION

Supplemental information can be found online at https://doi.org/10.1016/j.devcel.2023.08.017.

ACKNOWLEDGMENTS

We are grateful to Caetano Reis e Sousa, Francois Guillemot, Roberta Azzarelli, Andreas Wack, Giulio Cossu, Hans Stauss, Julia Rodrigues and Gitta Stockinger for helpful discussions. We thank Sahira Khalaf, Alexandra Kreins, Evey Howley, and Pei-Shi Chia for obtaining consents from patients for tissue donation and Laura De Rosa and Michele De Luca for providing anti-deltaNTP63-alpha antibody and epidermal cells. We thank Robert Goldstone; Jerome Nicod; Daniel Snell; Olga O'Neill of the Crick Advanced Sequencing team; the flow cytometry (Andy Riddell); experimental histopathology (Emma Nye and Susan Oguejiofor); and light microscopy (Donald Bell, Rocco D'Antuono, and Camille Charoy) science technology platforms. We thank the NanoString specialists Yan Liang and Mathias Holpert for their support with GeoMx analysis. P.B. would like to thank Yann Barrandon for introducing her to the thymus biology and for being an inspiring mentor. P.B. received funding from the European Research Council (ERC-Stg Agreement no. 639429), the Rosetrees Trust (M362; M362-F1; M553), the London Advanced Therapies – Research England (C2N-AT.006), the MRC Confidence in Concept scheme (award: MC_PC_17180), the Innovate UK (Smart grant no. 10005465), the CF Trust (SRC006; SRC020), the Duchenne Parent Project, and the National Institute for Health Research Biomedical Research Centre at Great Ormond Street Hospital for Children NHS Foundation Trust (NIHR GOSH BRC). R.R. is supported by a Marie Skłodowska-Curie Individual Fellowship 896014. S.A.W. is supported by London Interdisciplinary Biosciences Consortium (BBSRC). D.B. is supported by the Francis Crick Institute, which receives its core funding from Cancer Research UK (FC001045), the UK Medical Research Council (FC001045), and the Wellcome Trust (FC001045). A.B. is supported by the Junior EHA fellowship. A.C.H. is supported by funds from the Francis Crick Institute (FC001093) and by a WT Investigator Award (106292/Z/14/Z).

AUTHOR CONTRIBUTIONS

R.R. and P.B. designed experiments and analyzed the data. R.R. conducted experiments with the assistance of L.Z. and S.A.W. S.B. and G.D'A. performed bioinformatic analysis. M. Green conducted multiplex Akoya experiments. K.B. and A.A-D. conducted FACS sorting and supported analysis of flow-cytomentry data. A.B. provided guidance and analysis of 3D imaging of thick thymic sections. M. Greco performed nCounter NanoString analysis. L.A.-M. undertook i.v. injection of hematopoietic stem cells and animal model experiments. A.G. performed thymus microsurgery for scaffold preparation. D.S. and A.N. conducted GeoMx experiments and supported the analysis. R.R., A.C.H., D.B., and P.B. evaluated the results. The manuscript was written by P.B. and R.R. and was edited by A.C.H. and D.B., with input from all authors. Funding acquisition, P.B.; supervision, A.C.H., D.B., and P.B.

DECLARATION OF INTERESTS

P.B. and R.R. are named inventors of patent application no. P206246GB. P.B. is named inventor of patent application P124596GB. P.B. and A.G. are named inventors of patent application no. PCT/GB2019/051310.

Received: January 4, 2022
Revised: September 19, 2022
Accepted: August 9, 2023
Published: August 30, 2023

REFERENCES

1. Claudinot, S., Nicolas, M., Oshima, H., Rochat, A., and Barrandon, Y. (2005). Long-term renewal of hair follicles from clonogenic multipotent stem cells. Proc. Natl. Acad. Sci. USA *102*, 14677–14682. https://doi.org/10.1073/pnas.0507250102.
2. Watt, F.M. (2002). Role of integrins in regulating epidermal adhesion, growth and differentiation. EMBO J. *21*, 3919–3926. https://doi.org/10.1093/emboj/cdf399.
3. Rama, P., Bonini, S., Lambiase, A., Golisano, O., Paterna, P., De Luca, M., and Pellegrini, G. (2001). Autologous fibrin-cultured limbal stem cells permanently restore the corneal surface of patients with total limbal stem cell deficiency. Transplantation *72*, 1478–1485. https://doi.org/10.1097/00007890-200111150-00002.
4. Oshima, H., Rochat, A., Kedzia, C., Kobayashi, K., and Barrandon, Y. (2001). Morphogenesis and renewal of hair follicles from adult multipotent stem cells. Cell *104*, 233–245. https://doi.org/10.1016/s0092-8674(01)00208-2.
5. Rochat, A., Kobayashi, K., and Barrandon, Y. (1994). Location of stem cells of human hair follicles by clonal analysis. Cell *76*, 1063–1073. https://doi.org/10.1016/0092-8674(94)90383-2.
6. Barrandon, Y., and Green, H. (1987). Three clonal types of keratinocyte with different capacities for multiplication. Proc. Natl. Acad. Sci. USA *84*, 2302–2306. https://doi.org/10.1073/pnas.84.8.2302.
7. Sato, T., Vries, R.G., Snippert, H.J., van de Wetering, M., Barker, N., Stange, D.E., van Es, J.H., Abo, A., Kujala, P., Peters, P.J., and Clevers, H. (2009). Single Lgr5 stem cells build crypt-villus structures in vitro without a mesenchymal niche. Nature *459*, 262–265. https://doi.org/10.1038/nature07935.
8. Sato, T., van Es, J.H., Snippert, H.J., Stange, D.E., Vries, R.G., van den Born, M., Barker, N., Shroyer, N.F., van de Wetering, M., and Clevers, H. (2011). Paneth cells constitute the niche for Lgr5 stem cells in intestinal crypts. Nature *469*, 415–418. https://doi.org/10.1038/nature09637.
9. Rheinwald, J.G., and Green, H. (1975). Serial cultivation of strains of human epidermal keratinocytes: the formation of keratinizing colonies from single cells. Cell *6*, 331–343. https://doi.org/10.1016/s0092-8674(75)80001-8.
10. Blanpain, C., and Fuchs, E. (2014). Stem cell plasticity. Plasticity of epithelial stem cells in tissue regeneration. Science *344*, 1242281. https://doi.org/10.1126/science.1242281.
11. Bonfanti, P., Barrandon, Y., and Cossu, G. (2012). 'Hearts and bones': the ups and downs of 'plasticity' in stem cell biology. EMBO Mol. Med. *4*, 353–361. https://doi.org/10.1002/emmm.201200220.
12. Donati, G., and Watt, F.M. (2015). Stem cell heterogeneity and plasticity in epithelia. Cell Stem Cell *16*, 465–476. https://doi.org/10.1016/j.stem.2015.04.014.
13. Senoo, M., Pinto, F., Crum, C.P., and McKeon, F. (2007). p63 Is essential for the proliferative potential of stem cells in stratified epithelia. Cell *129*, 523–536. https://doi.org/10.1016/j.cell.2007.02.045.
14. Di Iorio, E., Barbaro, V., Ruzza, A., Ponzin, D., Pellegrini, G., and De Luca, M. (2005). Isoforms of DeltaNp63 and the migration of ocular limbal cells in human corneal regeneration. Proc. Natl. Acad. Sci. USA *102*, 9523–9528. https://doi.org/10.1073/pnas.0503437102.
15. Pellegrini, G., Dellambra, E., Golisano, O., Martinelli, E., Fantozzi, I., Bondanza, S., Ponzin, D., McKeon, F., and De Luca, M. (2001). p63 identifies keratinocyte stem cells. Proc. Natl. Acad. Sci. USA *98*, 3156–3161. https://doi.org/10.1073/pnas.061032098.
16. Enzo, E., Secone Seconetti, A., Forcato, M., Tenedini, E., Polito, M.P., Sala, I., Carulli, S., Contin, R., Peano, C., Tagliafico, E., et al. (2021). Single-keratinocyte transcriptomic analyses identify different clonal types and proliferative potential mediated by FOXM1 in human epidermal stem cells. Nat. Commun. *12*, 2505. https://doi.org/10.1038/s41467-021-22779-9.
17. Gui, J., Mustachio, L.M., Su, D.M., and Craig, R.W. (2012). Thymus Size and Age-related thymic Involution: early Programming, Sexual Dimorphism, Progenitors and stroma. Aging Dis. *3*, 280–290.
18. Wertheimer, T., Velardi, E., Tsai, J., Cooper, K., Xiao, S., Kloss, C.C., Ottmüller, K.J., Mokhtari, Z., Brede, C., deRoos, P., et al. (2018). Production of BMP4 by endothelial cells is crucial for endogenous thymic regeneration. Sci. Immunol. *3*, eaal2736. https://doi.org/10.1126/sciimmunol.aal2736.
19. Kreins, A.Y., Bonfanti, P., and Davies, E.G. (2021). Current and future therapeutic approaches for thymic stromal cell defects. Front. Immunol. *12*, 655354. https://doi.org/10.3389/fimmu.2021.655354.
20. McDonald-McGinn, D.M., Sullivan, K.E., Marino, B., Philip, N., Swillen, A., Vorstman, J.A., Zackai, E.H., Emanuel, B.S., Vermeesch, J.R., Morrow, B.E., et al. (2015). 22q11.2 deletion syndrome. Nat. Rev. Dis. Primers *1*, 15071. https://doi.org/10.1038/nrdp.2015.71.
21. Rossi, S.W., Jenkinson, W.E., Anderson, G., and Jenkinson, E.J. (2006). Clonal analysis reveals a common progenitor for thymic cortical and medullary epithelium. Nature *441*, 988–991. https://doi.org/10.1038/nature04813.
22. Bleul, C.C., Corbeaux, T., Reuter, A., Fisch, P., Mönting, J.S., and Boehm, T. (2006). Formation of a functional thymus initiated by a postnatal epithelial progenitor cell. Nature *441*, 992–996. https://doi.org/10.1038/nature04850.
23. Nusser, A., Sagar, X., Swann, J.B., Krauth, B., Diekhoff, D., Calderon, L., Happe, C., Grun, D., and Boehm, T. (2022). Developmental dynamics of two bipotent thymic epithelial progenitor types. Nature *606*, 165–171. https://doi.org/10.1038/s41586-022-04752-8.
24. Michelson, D.A., Hase, K., Kaisho, T., Benoist, C., and Mathis, D. (2022). Thymic epithelial cells co-opt lineage-defining transcription factors to eliminate autoreactive T cells. Cell *185*, 2542–2558.e18. https://doi.org/10.1016/j.cell.2022.05.018.
25. Sharma, H., and Moroni, L. (2021). Recent advancements in regenerative approaches for thymus rejuvenation. Adv. Sci. (Weinh) *8*, 2100543. https://doi.org/10.1002/advs.202100543.
26. Wong, K., Lister, N.L., Barsanti, M., Lim, J.M., Hammett, M.V., Khong, D.M., Siatskas, C., Gray, D.H., Boyd, R.L., and Chidgey, A.P. (2014). Multilineage potential and self-renewal define an epithelial progenitor cell population in the adult thymus. Cell Rep. *8*, 1198–1209. https://doi.org/10.1016/j.celrep.2014.07.029.
27. Ucar, A., Ucar, O., Klug, P., Matt, S., Brunk, F., Hofmann, T.G., and Kyewski, B. (2014). Adult thymus contains FoxN1(-) epithelial stem cells that are bipotent for medullary and cortical thymic epithelial lineages. Immunity *41*, 257–269. https://doi.org/10.1016/j.immuni.2014.07.005.
28. Sekai, M., Hamazaki, Y., and Minato, N. (2014). Medullary thymic epithelial stem cells maintain a functional thymus to ensure lifelong central T cell tolerance. Immunity *41*, 753–761. https://doi.org/10.1016/j.immuni.2014.10.011.
29. Ulyanchenko, S., O'Neill, K.E., Medley, T., Farley, A.M., Vaidya, H.J., Cook, A.M., Blair, N.F., and Blackburn, C.C. (2016). Identification of a bipotent epithelial progenitor population in the adult thymus. Cell Rep. *14*, 2819–2832. https://doi.org/10.1016/j.celrep.2016.02.080.
30. Depreter, M.G., Blair, N.F., Gaskell, T.L., Nowell, C.S., Davern, K., Pagliocca, A., Stenhouse, F.H., Farley, A.M., Fraser, A., Vrana, J., et al. (2008). Identification of Plet-1 as a specific marker of early thymic epithelial progenitor cells. Proc. Natl. Acad. Sci. USA *105*, 961–966. https://doi.org/10.1073/pnas.0711170105.
31. Swann, J.B., and Boehm, T. (2007). Back to the beginning–the quest for thymic epithelial stem cells. Eur. J. Immunol. *37*, 2364–2366. https://doi.org/10.1002/eji.200737709.

CellPress
OPEN ACCESS

32. Kinsella, S., and Dudakov, J.A. (2020). When the damage is done: injury and repair in thymus function. Front. Immunol. *11*, 1745. https://doi.org/10.3389/fimmu.2020.01745.

33. Bautista, J.L., Cramer, N.T., Miller, C.N., Chavez, J., Berrios, D.I., Byrnes, L.E., Germino, J., Ntranos, V., Sneddon, J.B., Burt, T.D., et al. (2021). Single-cell transcriptional profiling of human thymic stroma uncovers novel cellular heterogeneity in the thymic medulla. Nat. Commun. *12*, 1096. https://doi.org/10.1038/s41467-021-21346-6.

34. Park, J.E., Botting, R.A., Domínguez Conde, C., Popescu, D.M., Lavaert, M., Kunz, D.J., Goh, I., Stephenson, E., Ragazzini, R., Tuck, E., et al. (2020). A cell atlas of human thymic development defines T cell repertoire formation. Science *367*, eaay3224. https://doi.org/10.1126/science.aay3224.

35. Campinoti, S., Gjinovci, A., Ragazzini, R., Zanieri, L., Ariza-McNaughton, L., Catucci, M., Boeing, S., Park, J.E., Hutchinson, J.C., Muñoz-Ruiz, M., et al. (2020). Reconstitution of a functional human thymus by postnatal stromal progenitor cells and natural whole-organ scaffolds. Nat. Commun. *11*, 6372. https://doi.org/10.1038/s41467-020-20082-7.

36. Cowan, J.E., Malin, J., Zhao, Y., Seedhom, M.O., Harly, C., Ohigashi, I., Kelly, M., Takahama, Y., Yewdell, J.W., Cam, M., and Bhandoola, A. (2019). Myc controls a distinct transcriptional program in fetal thymic epithelial cells that determines thymus growth. Nat. Commun. *10*, 5498. https://doi.org/10.1038/s41467-019-13465-y.

37. Wu, X., Dao Thi, V.L., Huang, Y., Billerbeck, E., Saha, D., Hoffmann, H.H., Wang, Y., Silva, L.A.V., Sarbanes, S., Sun, T., et al. (2018). Intrinsic immunity shapes viral resistance of stem cells. Cell *172*, 423–438.e25. https://doi.org/10.1016/j.cell.2017.11.018.

38. Goldsmith, J.R., Spitofsky, N., Zamani, A., Hood, R., Boggs, A., Li, X., Li, M., Reiner, E., Ayyaz, A., Etwebi, Z., et al. (2020). TNFAIP8 controls murine intestinal stem cell homeostasis and regeneration by regulating microbiome-induced Akt signaling. Nat. Commun. *11*, 2591. https://doi.org/10.1038/s41467-020-16379-2.

39. Shapiro, B., Tocci, P., Haase, G., Gavert, N., and Ben-Ze'ev, A. (2015). Clusterin, a gene enriched in intestinal stem cells, is required for L1-mediated colon cancer metastasis. Oncotarget *6*, 34389–34401. https://doi.org/10.18632/oncotarget.5360.

40. Barbaro, V., Testa, A., Di Iorio, E., Mavilio, F., Pellegrini, G., and De Luca, M. (2007). C/EBPdelta regulates cell cycle and self-renewal of human limbal stem cells. J. Cell Biol. *177*, 1037–1049. https://doi.org/10.1083/jcb.200703003.

41. Moll, R., Divo, M., and Langbein, L. (2008). The human keratins: biology and pathology. Histochem. Cell Biol. *129*, 705–733. https://doi.org/10.1007/s00418-008-0435-6.

42. Kim, K.H., Rheinwald, J.G., and Fuchs, E.V. (1983). Tissue specificity of epithelial keratins: differential expression of mRNAs from two multigene families. Mol. Cell. Biol. *3*, 495–502. https://doi.org/10.1128/mcb.3.4.495-502.1983.

43. Bragulla, H.H., and Homberger, D.G. (2009). Structure and functions of keratin proteins in simple, stratified, keratinized and cornified epithelia. J. Anat. *214*, 516–559. https://doi.org/10.1111/j.1469-7580.2009.01066.x.

44. Bahrami, S., and Drabløs, F. (2016). Gene regulation in the immediate-early response process. Adv. Biol. Regul. *62*, 37–49. https://doi.org/10.1016/j.jbior.2016.05.001.

45. Santos, J., González-Sánchez, L., Villa-Morales, M., Ors, I., López-Nieva, P., Vaquero, C., González-Gugel, E., Fernández-Navarro, P., Roncero, A.M., Guenet, J.L., et al. (2010). The stromal gene encoding the CD274 antigen as a genetic modifier controlling survival of mice with gamma-radiation-induced T-cell lymphoblastic lymphomas. Oncogene *29*, 5265–5273. https://doi.org/10.1038/onc.2010.280.

46. Marchevsky, A.M., and Walts, A.E. (2017). PD-L1, PD-1, CD4, and CD8 expression in neoplastic and nonneoplastic thymus. Hum. Pathol. *60*, 16–23. https://doi.org/10.1016/j.humpath.2016.09.023.

47. Padda, S.K., Riess, J.W., Schwartz, E.J., Tian, L., Kohrt, H.E., Neal, J.W., West, R.B., and Wakelee, H.A. (2015). Diffuse high intensity PD-L1 staining in thymic epithelial tumors. J. Thorac. Oncol. *10*, 500–508. https://doi.org/10.1097/JTO.0000000000000429.

48. Jiang, X., Zhou, J., Giobbie-Hurder, A., Wargo, J., and Hodi, F.S. (2013). The activation of MAPK in melanoma cells resistant to BRAF inhibition promotes PD-L1 expression that is reversible by MEK and PI3K inhibition. Clin. Cancer Res. *19*, 598–609. https://doi.org/10.1158/1078-0432.CCR-12-2731.

49. Plasschaert, L.W., Žilionis, R., Choo-Wing, R., Savova, V., Knehr, J., Roma, G., Klein, A.M., and Jaffe, A.B. (2018). A single-cell atlas of the airway epithelium reveals the CFTR-rich pulmonary ionocyte. Nature *560*, 377–381. https://doi.org/10.1038/s41586-018-0394-6.

50. Guillemot, F., Lo, L.C., Johnson, J.E., Auerbach, A., Anderson, D.J., and Joyner, A.L. (1993). Mammalian achaete-scute homolog 1 is required for the early development of olfactory and autonomic neurons. Cell *75*, 463–476. https://doi.org/10.1016/0092-8674(93)90381-y.

51. Campbell, K.R., and Yau, C. (2019). A descriptive marker gene approach to single-cell pseudotime inference. Bioinformatics *35*, 28–35. https://doi.org/10.1093/bioinformatics/bty498.

52. Merritt, C.R., Ong, G.T., Church, S.E., Barker, K., Danaher, P., Geiss, G., Hoang, M., Jung, J., Liang, Y., McKay-Fleisch, J., et al. (2020). Multiplex digital spatial profiling of proteins and RNA in fixed tissue. Nat. Biotechnol. *38*, 586–599. https://doi.org/10.1038/s41587-020-0472-9.

53. Bartolini, A., Cardaci, S., Lamba, S., Oddo, D., Marchiò, C., Cassoni, P., Amoreo, C.A., Corti, G., Testori, A., Bussolino, F., et al. (2016). BCAM and LAMA5 mediate the recognition between tumor cells and the endothelium in the metastatic spreading of KRAS-mutant colorectal cancer. Clin. Cancer Res. *22*, 4923–4933. https://doi.org/10.1158/1078-0432.CCR-15-2664.

54. Udani, M., Zen, Q., Cottman, M., Leonard, N., Jefferson, S., Daymont, C., Truskey, G., and Telen, M.J. (1998). Basal cell adhesion molecule/Lutheran protein. The receptor critical for sickle cell adhesion to laminin. J. Clin. Invest. *101*, 2550–2558. https://doi.org/10.1172/JCI1204.

55. Popa, I., Zubkova, I., Medvedovic, M., Romantseva, T., Mostowski, H., Boyd, R., and Zaitseva, M. (2007). Regeneration of the adult thymus is preceded by the expansion of K5+K8+ epithelial cell progenitors and by increased expression of Trp63, cMyc and Tcf3 transcription factors in the thymic stroma. Int. Immunol. *19*, 1249–1260. https://doi.org/10.1093/intimm/dxm092.

56. Dotto, J., Pelosi, G., and Rosai, J. (2007). Expression of p63 in thymomas and normal thymus. Am. J. Clin. Pathol. *127*, 415–420. https://doi.org/10.1309/2GAYKPDDM85P2VEW.

57. Sun, S., Li, J.Y., Nim, H.T., Piers, A., Ramialison, M., Porrello, E.R., Konstantinov, I.E., Elefanty, A.G., and Stanley, E.G. (2022). CD90 marks a mesenchymal program in human thymic epithelial cells in vitro and in vivo. Front. Immunol. *13*, 846281. https://doi.org/10.3389/fimmu.2022.846281.

58. Hynds, R.E., Bonfanti, P., and Janes, S.M. (2018). Regenerating human epithelia with cultured stem cells: feeder cells, organoids and beyond. EMBO Mol. Med. *10*, 139–150. https://doi.org/10.15252/emmm.201708213.

59. Yamamoto, Y., and Ochiya, T. (2017). Epithelial stem cell culture: modeling human disease and applications for regenerative medicine. Inflamm. Regen. *37*, 3. https://doi.org/10.1186/s41232-017-0034-9.

60. Ward, R.K., Nation, P.N., Maxwell, M., Barker, C.L., and Clothier, R.H. (1997). Evaluation in vitro of epidermal cell keratinization. Toxicol. In Vitro *11*, 633–636. https://doi.org/10.1016/s0887-2333(97)00072-6.

61. Hirsch, T., Rothoeft, T., Teig, N., Bauer, J.W., Pellegrini, G., De Rosa, L., Scaglione, D., Reichelt, J., Klausegger, A., Kneisz, D., et al. (2017). Regeneration of the entire human epidermis using transgenic stem cells. Nature *551*, 327–332. https://doi.org/10.1038/nature24487.

62. Hamazaki, Y., Fujita, H., Kobayashi, T., Choi, Y., Scott, H.S., Matsumoto, M., and Minato, N. (2007). Medullary thymic epithelial cells expressing Aire represent a unique lineage derived from cells expressing claudin. Nat. Immunol. *8*, 304–311. https://doi.org/10.1038/ni1438.

63. Alawam, A.S., Anderson, G., and Lucas, B. (2020). Generation and regeneration of thymic epithelial cells. Front. Immunol. *11*, 858. https://doi.org/10.3389/fimmu.2020.00858.

64. Reggiani, P.C., Morel, G.R., Cónsole, G.M., Barbeito, C.G., Rodriguez, S.S., Brown, O.A., Bellini, M.J., Pléau, J.M., Dardenne, M., and Goya, R.G. (2009). The thymus-neuroendocrine axis: physiology, molecular biology, and therapeutic potential of the thymic peptide thymulin. Ann. N. Y. Acad. Sci. *1153*, 98–106. https://doi.org/10.1111/j.1749-6632.2008.03964.x.

65. Yang, Q., Bermingham, N.A., Finegold, M.J., and Zoghbi, H.Y. (2001). Requirement of Math1 for secretory cell lineage commitment in the mouse intestine. Science *294*, 2155–2158. https://doi.org/10.1126/science.1065718.

66. Perdigoto, C.N., Bardot, E.S., Valdes, V.J., Santoriello, F.J., and Ezhkova, E. (2014). Embryonic maturation of epidermal Merkel cells is controlled by a redundant transcription factor network. Development *141*, 4690–4696. https://doi.org/10.1242/dev.112169.

67. Nguyen, M.B., Valdes, V.J., Cohen, I., Pothula, V., Zhao, D., Zheng, D., and Ezhkova, E. (2019). Dissection of Merkel cell formation in hairy and glabrous skin reveals a common requirement for FGFR2-mediated signaling. Exp. Dermatol. *28*, 374–382. https://doi.org/10.1111/exd.13901.

68. van der Valk, W.H., van Beelen, E.S.A., Steinhart, M.R., Nist-Lund, C., Osorio, D., de Groot, J.C.M.J., Sun, L., van Benthem, P.P.G., Koehler, K.R., and Locher, H. (2023). A single-cell level comparison of human inner ear organoids with the human cochlea and vestibular organs. Cell Rep. *42*, 112623. https://doi.org/10.1016/j.celrep.2023.112623.

69. Petitpré, C., Faure, L., Uhl, P., Fontanet, P., Filova, I., Pavlinkova, G., Adameyko, I., Hadjab, S., and Lallemend, F. (2022). Single-cell RNA-sequencing analysis of the developing mouse inner ear identifies molecular logic of auditory neuron diversification. Nat. Commun. *13*, 3878. https://doi.org/10.1038/s41467-022-31580-1.

70. Wang, S., Lee, M.P., Jones, S., Liu, J., and Waldhaus, J. (2021). Mapping the regulatory landscape of auditory hair cells from single-cell multi-omics data. Genome Res. *31*, 1885–1899. https://doi.org/10.1101/gr.271080.120.

71. Wapinski, O.L., Vierbuchen, T., Qu, K., Lee, Q.Y., Chanda, S., Fuentes, D.R., Giresi, P.G., Ng, Y.H., Marro, S., Neff, N.F., et al. (2013). Hierarchical mechanisms for direct reprogramming of fibroblasts to neurons. Cell *155*, 621–635. https://doi.org/10.1016/j.cell.2013.09.028.

72. Park, N.I., Guilhamon, P., Desai, K., McAdam, R.F., Langille, E., O'Connor, M., Lan, X., Whetstone, H., Coutinho, F.J., Vanner, R.J., et al. (2017). ASCL1 reorganizes chromatin to direct neuronal fate and suppress tumorigenicity of glioblastoma stem cells. Cell Stem Cell *21*, 411. https://doi.org/10.1016/j.stem.2017.08.008.

73. Borges, M., Linnoila, R.I., van de Velde, H.J., Chen, H., Nelkin, B.D., Mabry, M., Baylin, S.B., and Ball, D.W. (1997). An achaete-scute homologue essential for neuroendocrine differentiation in the lung. Nature *386*, 852–855. https://doi.org/10.1038/386852a0.

74. Raposo, A.A.S.F., Vasconcelos, F.F., Drechsel, D., Marie, C., Johnston, C., Dolle, D., Bithell, A., Gillotin, S., van den Berg, D.L.C., Ettwiller, L., et al. (2015). Ascl1 coordinately regulates gene expression and the chromatin landscape during neurogenesis. Cell Rep. *10*, 1544–1556. https://doi.org/10.1016/j.celrep.2015.02.025.

75. Girard, N. (2013). Thymic epithelial tumours: from basic principles to individualised treatment strategies. Eur. Respir. Rev. *22*, 75–87. https://doi.org/10.1183/09059180.00007312.

76. Kaur, P., and Li, A. (2000). Adhesive properties of human basal epidermal cells: an analysis of keratinocyte stem cells, transit amplifying cells, and postmitotic differentiating cells. J. Invest. Dermatol. *114*, 413–420. https://doi.org/10.1046/j.1523-1747.2000.00884.x.

77. Anderson, G., and Jenkinson, W.E. (2015). Border control: anatomical origins of the thymus medulla. Eur. J. Immunol. *45*, 2203–2207. https://doi.org/10.1002/eji.201545829.

78. Adams, J.C., and Watt, F.M. (1989). Fibronectin inhibits the terminal differentiation of human keratinocytes. Nature *340*, 307–309. https://doi.org/10.1038/340307a0.

79. Abdulhasan, M., Ruden, X., Rappolee, B., Dutta, S., Gurdziel, K., Ruden, D.M., Awonuga, A.O., Korzeniewski, S.J., Puscheck, E.E., and Rappolee, D.A. (2021). Stress decreases Host Viral Resistance and Increases Covid Susceptibility in Embryonic Stem Cells. Stem Cell Rev. Rep. *17*, 2164–2177. https://doi.org/10.1007/s12015-021-10188-w.

80. Rajapaksa, U.S., Jin, C., and Dong, T. (2020). Malignancy and IFITM3: friend or foe? Front. Oncol. *10*, 593245. https://doi.org/10.3389/fonc.2020.593245.

81. Kenney, A.D., McMichael, T.M., Imas, A., Chesarino, N.M., Zhang, L., Dorn, L.E., Wu, Q., Alfaour, O., Amari, F., Chen, M., et al. (2019). IFITM3 protects the heart during influenza virus infection. Proc. Natl. Acad. Sci. USA *116*, 18607–18612. https://doi.org/10.1073/pnas.1900784116.

82. Colantonio, A.D., Epeldegui, M., Jesiak, M., Jachimowski, L., Blom, B., and Uittenbogaart, C.H. (2011). IFN-alpha is constitutively expressed in the human thymus, but not in peripheral lymphoid organs. PLoS One *6*, e24252. https://doi.org/10.1371/journal.pone.0024252.

83. Bonfanti, P., Claudinot, S., Amici, A.W., Farley, A., Blackburn, C.C., and Barrandon, Y. (2010). Microenvironmental reprogramming of thymic epithelial cells to skin multipotent stem cells. Nature *466*, 978–982. https://doi.org/10.1038/nature09269.

84. Rama, P., Matuska, S., Paganoni, G., Spinelli, A., De Luca, M., and Pellegrini, G. (2010). Limbal stem-cell therapy and long-term corneal regeneration. N. Engl. J. Med. *363*, 147–155. https://doi.org/10.1056/NEJMoa0905955.

85. Bankhead, P., Loughrey, M.B., Fernández, J.A., Dombrowski, Y., McArt, D.G., Dunne, P.D., McQuaid, S., Gray, R.T., Murray, L.J., Coleman, H.G., et al. (2017). QuPath: open source software for digital pathology image analysis. Sci. Rep. *7*, 16878. https://doi.org/10.1038/s41598-017-17204-5.

86. de Chaumont, F., Dallongeville, S., Chenouard, N., Hervé, N., Pop, S., Provoost, T., Meas-Yedid, V., Pankajakshan, P., Lecomte, T., Le Montagner, Y., et al. (2012). Icy: an open BioImage informatics platform for extended reproducible research. Nat. Methods *9*, 690–696. https://doi.org/10.1038/nmeth.2075.

87. Ahlmann-Eltze, C., and Huber, W. (2021). glmGamPoi: fitting Gamma-Poisson generalized linear models on single cell count data. Bioinformatics *36*, 5701–5702. https://doi.org/10.1093/bioinformatics/btaa1009.

88. Hao, Y., Hao, S., Andersen-Nissen, E., Mauck, W.M., 3rd, Zheng, S., Butler, A., Lee, M.J., Wilk, A.J., Darby, C., Zager, M., et al. (2021). Integrated analysis of multimodal single-cell data. Cell *184*, 3573–3587.e29. https://doi.org/10.1016/j.cell.2021.04.048.

89. Cao, J., Spielmann, M., Qiu, X., Huang, X., Ibrahim, D.M., Hill, A.J., Zhang, F., Mundlos, S., Christiansen, L., Steemers, F.J., et al. (2019). The single-cell transcriptional landscape of mammalian organogenesis. Nature *566*, 496–502. https://doi.org/10.1038/s41586-019-0969-x.

90. Risso, D., Ngai, J., Speed, T.P., and Dudoit, S. (2014). Normalization of RNA-seq data using factor analysis of control genes or samples. Nat. Biotechnol. *32*, 896–902. https://doi.org/10.1038/nbt.2931.

91. Love, M.I., Huber, W., and Anders, S. (2014). Moderated estimation of fold change and dispersion for RNA-seq data with DESeq2. Genome Biol. *15*, 550. https://doi.org/10.1186/s13059-014-0550-8.

92. Stuart, T., Butler, A., Hoffman, P., Hafemeister, C., Papalexi, E., Mauck, W.M., 3rd, Hao, Y., Stoeckius, M., Smibert, P., and Satija, R. (2019). Comprehensive integration of single-cell data. Cell *177*, 1888–1902.e21. https://doi.org/10.1016/j.cell.2019.05.031.

93. Bhattacharya, A., Hamilton, A.M., Furberg, H., Pietzak, E., Purdue, M.P., Troester, M.A., Hoadley, K.A., and Love, M.I. (2021). An approach for normalization and quality control for NanoString RNA expression data. Brief. Bioinform. *22*, bbaa163. https://doi.org/10.1093/bib/bbaa163.

STAR★METHODS

KEY RESOURCES TABLE

REAGENT or RESOURCE	SOURCE	IDENTIFIER
Antibodies		
BCAM BV421	BD Bioscience	CAT#748007; RRID:AB_2872468
CD24 APC	BioLegend	CAT#311117; RRID:AB_1877150
CD24 BV605	BioLegend	CAT#311124; RRID:AB_2562288
CD205 PE	BioLegend	CAT#342203; RRID:AB_1626209
CD235ab Biotin	BioLegend	CAT#306618; RRID:AB_2565773
CD45 Biotin BioLegend	BioLegend	CAT#103104; RRID:AB_312969
CD45 APC	BioLegend	CAT#304011; RRID:AB_314399
CD49f AF488	BioLegend	CAT#313608; RRID:AB_493635
C49f BV421	BioLegend	CAT#313624; RRID:AB_2562244
CD90 AF700	BioLegend	CAT#328119; RRID:AB_2203303
CD90 FITC	BioLegend	CAT#328108; RRID:AB_893429
EpCAM PE-Cy7	BioLegend	CAT#324222; RRID:AB_2561506
Mouse Feeder	Miltenyi	CAT#30120166; RRID:AB_2752027
Ly75 (CD205)	Biolegend	CAT#313608; RRID:AB_493635
CK5	Abcam	CAT#AB124897; RRID:AB_10976058
CK7	Abcam	CAT#AB17130; RRID:AB_443671
CK10	Sigma Aldrich	CAT#HPA007272;RRID:AB_1079181
CK13	Santa Cruz	CAT#SC-53252;RRID:AB_629835
CK14	Abcam	CAT#AB16112;RRID:AB_302267
CK15	Biolegend	CAT#905301;RRID:AB_2565048
CK17	Sigma Aldrich	CAT#HPA023910;RRID:AB_1852205
CK8	Sigma Aldrich	CAT#HPA000453;RRID:AB_1079176
CK8/18	Abcam	CAT#AB9023;RRID:AB_306948
EpCAM- efluor660 cojugated	eBioscience	CAT#BP5075;RRID:AB_979823
p63	Abcam	CAT#50-9326-42;RRID:AB_10598658
IFITM3	Thermofisher	CAT#AB735;RRID:AB_305870
FN1	Proteintech	CAT#MA5-32798;RRID:AB_2810074
THY-1 (CD90)	Biolegend	CAT#15613-1-AP;RRID:AB_2810074
deltaNTP63alpha	kindly provided by Michele De Luca	NA
BCAM	BD Bioscience	CAT#748007;RRID:AB_2872468
BCAM	Novus Biological	CAT#NBP2-31994;RRID:AB_2922815
CD24-APC conjugated	BioLegend	CAT#311117;RRID:AB_2922815
ASCL1	Abcam	CAT#AB74065;RRID:AB_1859937
TIMP1	Invitrogen	CAT#MA1-773;RRID:AB_889482
SOX2	R&D Systems	CAT#AF2018;RRID:AB_355110
CD45	Abcam	CAT#AB40763;RRID:AB_726545
CD45	Novus Biological	CAT#NBP2-34528;RRID:AB_2864384
CD3E	Origene	CAT#UM500048;RRID:AB_2629062
CD3 APC conjugated	BioLegend	CAT#300411;RRID:AB_314065
DES	Agilent/DAKO	CAT#M0760;RRID:AB_2335684
Anti-Guinea Pig AF594	Jackson Immuno	CAT#706-165-148; RRID:AB_2340460
Anti-Mouse AF488	Jackson Immuno	CAT#715-545-150; RRID:AB_2340846
Anti-Mouse AF594	Jackson Immuno	CAT#715-165-150; RRID:AB_2340813
Anti-Mouse AF647	Jackson Immuno	CAT#715-605-150; RRID:AB_2340862

(*Continued on next page*)

Continued		
REAGENT or RESOURCE	SOURCE	IDENTIFIER
Anti-Rabbit AF488	Jackson Immuno	CAT#711-545-152; RRID:AB_2313584
Anti-Rabbit AF594	Jackson Immuno	CAT#711-585-152; RRID:AB_2340621
Anti-Rabbit AF647	Jackson Immuno	CAT#711-605-152; RRID:AB_2492288
Anti-Rat AF488	Jackson Immuno	CAT#712-545-150; RRID:AB_2340683
Anti-Rat AF647	Jackson Immuno	CAT#712-605-150; RRID:AB_2340693
Anti-Chicken AF488	Jackson Immuno	CAT#703-225-155; RRID:AB_2340370
Anti-Chicken AF594	Jackson Immuno	CAT#705-585-155; RRID: AB_2340377
Anti-Goat AF488	Jackson Immuno	CAT#705-545-147; RRID:AB_2336933
Anti-Goat AF594	Jackson Immuno	CAT#705-165-147; RRID:AB_2307351
Biological Samples		
Human Postnatal Thymi	Great Ormond Street Hospital (GOSH)-London	https://www.gosh.nhs.uk
Human Foetal liver	HDBR UK	https://www.hdbr.org
Chemicals, peptides, and recombinant proteins		
Collagenase D	Roche	cat # 11088866001
Collagenase A	Roche	cat# 10103578001
Dispase II	Gibco	cat# 17105-041
ROCHE DNase Igrade II from bovine pancreas	Roche	cat# 10104159001
DAPI	Sigma-Aldrich	cat# D9542
Hoescht 33342	Sigma-Aldrich	cat# B2261
Zombie Aqua Fixable Viability Kit (DMSO)	Biolegend	cat# 423102
Pneumacult ALI Basal Medium	Stemcell Technologies	cat# 05002
Pneumacult ALI 10X supplement	Stemcell Technologies	cat# 05003
Hydrocortisone Stock Solution	Stemcell Technologies	cat# 07925
Pneumacult ALI 100X maintenance	Stemcell Technologies	cat# 05006
Buffer RLT Plus	Qiagen	cat# 1030963
Triton X-100	Sigma-Aldrich	cat# T8787
Normal Donkey Serum Powder	Jackson IMMUNO	cat# 017-000-121
RPMI 1640, GlutaMAX(TM)	Gibco	cat# 61870010
Hanks' Balanced Salt solution	Sigma-Aldrich	cat# H6648
DMEM, high glucose	Gibco	cat# 41965039
Gibco™ Ham's F-12 Nutrient Mix	Gibco	cat # 21765029
Penicillin -streptomycin solution*stabilized	Sigma-Aldrich	cat# p4333-100
Cholera Toxin from Vibrio cholerae	Sigma-Aldrich	cat# 80052
3,3′,5-Triiodo-L-thyronine sodium salt	Sigma-Aldrich	cat# T6397
Insulin, Human Recombinant	Sigma Aldrich	cat# 91077C
Hydrocortisone, Chromatographic Standard	Sigma Aldrich	cat# 386698
Sodium Deoxycholate	Sigma Aldrich	cat# 30970
DNase I from bovine pancreas	Sigma Aldrich	cat# D5025
Minimum essential medium. Non-essential amminoacids	Gibco	cat#. 11140-035
L-Glutamine 200mM	Gibco	cat# 25030-024
2-Mercaptoethanol 50mM	Gibco	cat# 31350-010
Human Epidermal Growth factor	Peprotech	cat# AF10015
Fetal Bovine Serum	Sigma-Aldrich	cat# F2442
Fetal Bovine Serum, qualified, heat inactivated, Brazil	Sigma-Aldrich	cat# 10500064
MegaCell™ Dulbecco's Modified Eagle's Medium	Sigma-Aldrich	cat# M3942
TrypLE™ Express Enzyme (1X), no phenol red	Life Technologies	cat# 12604021
HyClone Calf Serum, U.S. origin	Hyclone	cat# SH3007203

(*Continued on next page*)

Continued

REAGENT or RESOURCE	SOURCE	IDENTIFIER
Human FGF-Basic, recombinant	Sigma Aldrich	cat# F0291
HumanKine™ Recombinant Human SCF Protein	Proteintech	cat # HZ-1024
HumanKine® recombinant human FLT3 Ligand protein	Proteintech	cat# HZ-1151
HumanKine® recombinant human IL-7 protein	Proteintech	cat# HZ-1281
Rhodamine B	Sigma-Aldrich	cat# R6626
UltraPure™ Low Melting Point Agarose	Thermofisher	cat# 16520050
Benzyl benzoate	Sigma-Aldrich	cat# W213802
Benzyl alcohol	Sigma-Aldrich	cat# 108006
Paraformaldehyde reagent grade, crystalline	Sigma-Aldrich	cat# P6148
MagniSort™ Streptavidin Negative Selection Beads	Invitrogen	cat# MSNB-6002-74
O.C.T.	VWR	cat# 361603E
Mounting Medium	Abcam	cat# ab104139
HyClone Medium 199	Hyclone	cat# SH30253.01
Gelatin	Sigma Aldrich	cat# G1393
Antibiotic-Antimycotic 100X-solution	Invitrogen	cat# 15240-062
HEPES	Gibco	cat# 15630080
Heparin	Sigma-Aldrich	cat# 9041-08-1
ECGS	Sigma-Aldrich	cat# E2759
Trypan bllue solution	Sigma-Aldrich	cat# T8154
**Critical commercial assays**		
Chromium Next GEM Single Cell 3' Kit v3.1	10-x genomics	cat# PN-1000268
nCounter® Stem Cell Characterization Panel	Nanostring Technologies	cat# XT-CSO-HSCC-12
GeoMx Human Whole Transcriptome Atlas	Nanostring Technologies	Merritt et al.[52]
MagniSort™ Streptavidin Negative Selection Beads	Invitrogen	cat# MSNB-6002-74
EasySep™ Human CD34 Positive Selection Kit II	Stemcell Technologies	cat# 17856
Precision PLUS 2x 2x qPCR Master Mix with LR	Primer Design	cat# PPLUS-LR
RELIA Prep rna extraction kit	Promega	cat# Z6011
Bond Polymer Refine detection kit	Leica	cat# DS9800
Opal 520 Reagent Pack	Akoyabio	cat# FP1487001KT
Opal 690 Reagent Pack	Akoyabio	cat# FP1497001KT
Opal 570 Reagent Pack	Akoyabio	cat# FP1488001KT
**Deposited data**		
Sc-RNA-seq Human postnatal thymus	This paper	GSE220830
Sc-RNA-seq cultured human airways and skin	This paper	GSE220207
Sc-RNA-seq cultured human TEC	This paper	GSE220206
Sc-RNA-seq cultured human thymic cortical and medullary progenitor/stem cells and skin keratinocyte	This paper	GSE220829
**Experimental models: Cell lines**		
3T3-J2 cells	originally developed in Howard Green laboratory in Harvard Medical School	N/A
Human Thymic epithelial and interstitial cells patient derived cultures	derived as described from consented patients undergoing cardiothoracic surgery at the Great Ormond Street Hospital	N/A

(*Continued on next page*)

Continued		
REAGENT or RESOURCE	SOURCE	IDENTIFIER
Human Skin Keratinocytes patient derived cultures	kindly donated by Michele De Luca, University of Modena and Reggio-Emilia, Italy	N/A
Primary normal human Bronchial/Tracheal Epithelial Cells	ATCC	CAT# PCS-300-010
HuVEC-VeraVEC	Angiocrine	CAT# HVERA101
Experimental models: Organisms/strains		
Mouse	N/A	N/A
NOD.Cg-Prkdcscid Il2rgtm1Wjl/SzJ	Jackson	CAT #:005557; RRID:IMSR_JAX:005557
NOD.Cg-Foxn1em1Dvs Prkdcscid Il2rgtm1Wjl/J	Jackson	CAT# 026263; RRID:IMSR_JAX:026263
Rat Wistar Han IGS	Charles River	CAT# 273
Oligonucleotides		
Primers for Thymic epithelial cells please see Table S3	This paper	N/A
Software and algorithms		
QuPath v0.3.2	https://qupath.github.io	Bankhead et al.[85]
FlowJo 10.7.2	https://www.flowjo.com	N/A
ImageJ v2.0.0-rc-69/1.52p	http://www.imagej.net	N/A
BD FACSDiva™ Software v8.0.1	https://www.bdbiosciences.com/en-gb/products/software/instrument-software/bd-facsdiva-software	N/A
ZEISS ZEN lite blue 2.3	https://www.zeiss.com/microscopy/en/products/software/zeiss-zen-lite.html	N/A
ZEISS ZEN lite black 2.3 v14.0.21.201	https://www.zeiss.com/microscopy/en/products/software/zeiss-zen-lite.html	N/A
QuantStudio Design Analysis Software v1.4	https://www.thermofisher.com/uk/en/home/global/forms/life-science/quantstudio-3-5-software.html	N/A
Olympus cellSens Entry v1.18	https://www.olympus-lifescience.com/en/software/cellsens/	N/A
Imaris v9.5.1	http://www.bitplane.com/imaris/imaris	N/A
Icy 2.4.2.0	http://icy.bioimageanalysis.org/javadoc/	De Chaumont et al.[86]
glmGamPoi R-package version 1.2.0	https://rdrr.io/bioc/glmGamPoi	Ahlmann-Eltze et al.[87]
nSolver Analysis Software v4.0	https://nanostring.com	N/A
Prism 9.4.0	https://www.graphpad.com	N/A
GeoMx Digital Spatial Profiler v2.0	https://nanostring.com/products/geomx-digital-spatial-profiler/software-updates/v2-0/	Merritt et al.[52]
GeoMx NGS Pipeline Dataset Package	https://nanostring.com/products/geomx-digital-spatial-profiler/software-updates/v2-0/	Merritt et al.[52]
Phenochart version 1.0.2 software	https://www.akoyabio.com/support/software/phenochart-whole-slide-viewer/	N/A
inForm®	https://www.akoyabio.com/phenoimager/software/inform-tissue-finder/	N/A
R version 4.0.3	https://www.rstudio.com/products/rstudio/	https://www.R-project.org/
R-package Seurat 4.0.5	https://satijalab.org/seurat/articles/install.html	Hao et al.[88]
R-package glmGamPoi 1.2.0	https://bioconductor.org/packages/release/bioc/html/glmGamPoi.html	Ahlmann-Eltze et al.[87]
R-package monocle3 0.2.2	https://cole-trapnell-lab.github.io/monocle3/docs/installation/	Cao et al.[89]
R-package ouija 0.99.1	https://rdrr.io/github/kieranrcampbell/ouija/	Campbell et al.[51]

(*Continued on next page*)

Continued

REAGENT or RESOURCE	SOURCE	IDENTIFIER
R-package RUVSeq 1.24.0	https://bioconductor.org/packages/release/bioc/html/RUVSeq.html	Risso et al.[90]
R-package DESeq2 1.28.1	https://bioconductor.org/packages/release/bioc/html/DESeq2.html	Love et al.[91]

RESOURCE AVAILABILITY

Lead contact

Further information and requests for resources and reagents should be directed to and will be fulfilled by the lead contact, Paola Bonfanti, Email: paola.bonfanti@crick.ac.uk or p.bonfanti@ucl.ac.uk.

Materials availability

Human primary thymic cells used in this study will be shared by the lead contact upon request and signed MTA.

Data and code availability

All single cell RNA-sequencing data have been deposited at GEO and are publicly available as of the date of publication. Accession numbers are listed in the key resources table.

Microscopy data reported in this paper will be shared by the lead contact upon request.

Any additional information required to reanalyse the data reported in this paper is available from the lead contact upon request.

EXPERIMENTAL MODEL AND STUDY PARTICIPANT DETAILS

Human tissues

Postnatal thymi were donated by patients undergoing cardiothoracic surgery at the Great Ormond Street Hospital. Written informed consent was obtained from the patients or legally authorised representatives under ethical approval (REC No 15/YH/0334 and 07/Q0508/43-06-MI-13). Human foetal liver was provided by the Joint MRC/Wellcome Trust Human Developmental Biology Resource (HDBR) under informed ethical consent with Research Tissue Bank ethical approval (REC No 08/H0712/34+5 and 08/H0906/21+5).

Animal models

NOD.Cg-Prkdcscid.Il2Rγctm1Wjl (NSG) and NOD.Cg-Foxn1em1Dvs.Prkdcscid.Il2Rγctm1Wjl (NSG-Nude, Stock No: 026263) were obtained from Jackson Laboratory, re-derived and maintained at The Francis Crick Institute's biological resource facility. Mice are bred in isolators with aseptic standard operating procedures in the Biological Research Facility of The Francis Crick Institute under specific pathogen-free conditions. Once weaned, mice were kept in ventilated cages.

All animal experiments were performed in accordance with ethical approval under the UK Home Office Project License (PPL) PP9619702 in accordance with The Francis Crick Institute animal ethics committee guidance.

CD and Wistar Han rats were purchased from Charles River Laboratories.

METHOD DETAILS

Thymic epithelial cell (TEC) isolation and sorting

Thymic tissue fragments were dissociated to single cell suspension with enzymatic treatment (0.4 mg/mL Collagenase D (Roche), 0.6 mg/mL Dispase II (Gibco), 40 μg/mL DNAse I (Roche)) for around 30-45 minutes, using the Gentle MACS machine (Miltenyi). After the dissociation, the supernatant was collected, passed through a cell-strainer (100μm), centrifuged at 1200 r.p.m. for 5 minutes and counted with trypan blue (SIGMA-ALDRICH) to assess viability. One portion of total cell suspension was used for culture (see below) and the other depleted for $CD45^+$ and $CD235^+$ cells by staining them with biotinylated antibodies (see Table S2), then incubating with magnetic negative beads (Magnisort SAV Negative Beads, Invitrogen) and placing the suspension into a magnet (STEMCELL Technologies) for 10 min. The flowthrough fraction was collected, and the enrichment step was repeated at least three times. The final enriched fraction ($CD235^-CD45^-$) was stained for surface markers to isolate epithelial cells. Cells were sorted using FACS Aria III or Fusion machine (BD) and sorted events were plated in culture or were lysated in RTL (Qiagen) for transcriptomic analysis or processed for 10-X single cell sequencing.

Epithelial stem cell culture (thymus, skin and airways cells)

Thymic epithelial cells, skin keratinocytes or airways basal cells derived from the dissociation and/or sorting, were plated on a layer of sub-lethally irradiated mouse fibroblast (3T3-J2) as described previously.[9] These cells were kept in culture with cFAD medium composed by a mixture of 3:1 of DMEM1X (Gibco) and F-12 Nut Mix (Gibco), 10% Fetal Bovine Serum (SIGMA-ALDRICH), 1%

penicillin and streptomycin (100X, Sigma), Hydrocortisone (0.4 μg/ml, Calbiochem), Cholera Toxin (10^{-10} M, Sigma), Triodothyronine (T3) ($2x10^{-9}$ M Sigma) and Insulin (5 μg/ml, SIGMA-ALDRICH). All the reagents were filtered through a 0.22 μm strainer. Epithelial cultures cells were kept in incubator at 37°C in a 6% CO_2 atmosphere. Human epithelial growth factor (hEGF, 10 ng/ml, PeproTech) was added to the cultures after three days and then every other day. Epithelial cells were plated at a density of 2000-6000 cells/cm^2. Once sub-confluent, epithelial cells were harvested using TrypLe express (Gibco) for 3-5 minutes at 37°C, blocked with medium, spun down 1200 rpm and counted.

The colony forming efficiency assay (CFE) or plating efficiency (PE) was performed every other passage. A specific number of cells (i.e., 300-500 cells for cultures; 500-1500 for sorted events) were plated by a serial dilution in MW6 or 60mm dishes previously seeded with lethally irradiated 3T3-J2 cells. At day 4 and 8, the culture was supplemented with hEGF (10 ng/ml, PeproTech). After 12 days, cultured cells were fixed by 4% Paraformaldehyde (PFA, SIGMA-ALDRICH) for 10 minutes and stained with Rhodamine-B (1%, SIGMA-ALDRICH) for 15 min. The dish was washed with tap water and left to dry at room temperature.

Single cell cloning was performed as follow: thymic epithelial cells were trypsinized and counted as described above. Once a single-cell suspension was obtained, serial dilutions were made to plate one single cell in each well of 12 or 48-well plates, pre-coated with a layer of sub-lethally irradiated mouse fibroblast (3T3-J2).

Coverslips immunophenotypic analysis: epithelial cells were seeded at the density of 1200-2500 cells/cm^2 per each well (12 wells plate) onto glass coverslips pre-seeded with irradiated 3T3-J2 and cultured up to 7 days. Then, cultured cells were fixed by 4% Paraformaldehyde (PFA, SIGMA-ALDRICH) for 10 minutes and washed twice with PBS and kept at 4C until the immunofluorescence (IF) staining.

Quality control of primary cell cultures included Mycoplasma PCR screening, STR authentication to confirm unique profile and KarioStat™ Array (ThermoFisher, cat# 905403) to screen for possible chromosomal abnormalities.

Differentiation of thymic epithelial cultures

Thymic SC were plated onto a membrane insert for well plates (Greiner Bio) with a density of 400-800 cells/mm^2 and cultured in cFAD until day 2. Expansion media was replaced with differentiation media according to manufacturer's instructions (PneumaCult™, STEMCELL Technologies) at day 3 and changed every other day for 25 to 30 days when cells were harvested for immunofluorescence (IF) and gene expression analysis (qRT-PCR). For IF, the membrane was washed three times with PBS and then fixed with 4% PFA (SIGMA-ALDRICH) for 10 min. After fixation, the membrane was washed three times in PBS and stored at 4°C or used immediately for immunostaining. Alternatively, the membrane was covered and washed with Lysis buffer+Thtoglycerol buffer (Promega) for the collection of RNA and stored at -80°C until extraction.

Flow cytometry analysis

Single-cell suspensions were stained with *ad hoc* antibody mix in Hanks Balanced Salt Solution (HBSS, Life Technologies) supplemented with 2% FBS (Life Technologies) for 30min on ice. DAPI (SIGMA-ALDRICH) or Zombie Live-Dead dye (Invitrogen) was used to discriminate live from dead cells.

FACS phenotypic analysis was performed using Fortessa X-20 machine (BD Bioscience) and FlowJo™ software.

A complete list of antibodies used for FACS staining is available in Table S2.

In vivo assay: grafting thymic rat scaffolds into NSG-Nude mice

Rat thymi vascular microsurgery, perfusion and decellularization has been performed as described.

Mice humanisation protocol was performed using CD34+ isolated from 18 weeks post conception (wpc) human foetal liver samples and scaffold repopulation was achieved as previously described (35).

Sub-cutaneous implantation of the scaffold was performed in NSG and NSG-Nude mice 4 to 5 weeks post-CD34+ injection. Mice were culled and scaffold harvested at 10- and 16-week post-transplant (wpt). We carried out subcutaneous transplantation in three humanized NSG-Nude and three humanized NSG mice: all mice showed bone marrow reconstitution. The mice were implanted each with 4 repopulated scaffolds for a total of 24 repopulated scaffolds, 20 of which were retrieved.

RNA isolation and real-time quantitative PCR

Cultured cells were collected for gene expression analysis in Lysis buffer+Thyoglycerol buffer from ReliaPrep™ kit (Promega) following the manufacturer's instructions. Precipitated and dried RNA was re-suspended in nuclease free water (Qiagen). RNA concentration was measured using Nanodrop1000 (ThermoScientific). RNA was converted into cDNA with GoScript™ Reverse Transcriptase kit (Promega) according to the manufacturer's protocol. cDNA concentration was adjusted to 10ng/μl. Quantitative (q) PCR was performed using PCR master mix (PrecisionPLUS-R, Primerdesign Ltd) with low-ROX and Taqman qPCR probes (Integrated DNA Technology, Table S3) in MicroAmp Fast Optical 96 well Reaction Plates (Applied Biosystems) using the QuantStudio 3 Real-Time PCR System (Applied Biosystems).

Single-cell RNA sequencing – analysis of thymic fresh tissue and cultivated cells

Trypsinized cells and FACS-sorted events were resuspended in final volume of 50μl of HBSS+0.04%BSA solution.

Cell numbers were confirmed using an Eve automated cell counter (NanoEnTek). Where possible an appropriate volume for 10,000 cells was adjusted with nuclease-free water. Reverse transcription and library construction were prepared by following Chromium

single-cell 3′ reagent v3 protocol (10X Genomics) according to the manufacturer's recommendations. Total complementary-DNA synthesis was performed using 12 amplification cycles, with final cDNA yields ranging from ~3 ng/μl to 15 ng/μl. The 10X Genomics single cell RNA-seq libraries were constructed as described and sequenced on an Illumina HiSeq 4000.

Bioinformatics data analysis of single-cell data

10X FASTQ-files were aligned with the CellRanger toolkit (10X Genomics, version 5.0.0) toolkit to the Ensembl human GRCh38 reference transcriptome. To identify mouse feeder cells in the *in vitro* experiment, the *in vitro* dataset was also aligned to the combined human GRCh38 and mouse mm10 reference transcriptome. Mouse feeder cells were filtered from the overall cell population.

The filtered count tables output by CellRanger were further analyzed using the Seurat R-package (version 4.0.5)[92] and filtered based on the following percentages of mitochondrial genes: *in vivo* samples: mTEC < 20%; cTEC < 30%; all *in vitro* culture samples were filtered at < 20%; cells were retained if they had at least 200 detected genes (nFeature) for all *in vivo* samples and at > 750 genes for all *in vitro* samples. For *in vivo* datasets, we profiled 3872 cells for sorted EpCAM^{low}CD205^{pos} (cortex) and 4935 cells for sorted EpCAM^{high}CD205^{neg} (medulla); 1349 cells for cTEC CD49f^{pos} and 1414 for mTEC CD49f^{pos}. For *in vitro* datasets, we profiled 2798 and 1871 cells for sorted cTEC; 3463 and 3430 cells for sorted mTEC; 3463 and 3430 cells for unsorted TEC cultures; and 5509 cells for skin keratinocytes cultures after performing QC and mouse feeder layer reads removal.For the second *in vitro* dataset, two additional replicates of thymic epithelial cells (5968 and 3876 cells), one of skin keratinocytes (7633 cells) and one of basal airways cells (4237 cells) have been profiled after performing QC and mouse feeder layer reads removal. Individual samples in the *in vitro* and *in vivo* experiment were integrated using the canonical correlation analysis method of the Seurat R-package.

Counts were normalized and scaled using the NormalizeData and ScaleData functions from Seurat, using default parameters. For dimensionality reduction highly variable genes were identified using the FindVariableFeatures function and the "vst" method from Seurat, keeping the top 2000 most variable features, and using them as input for Principal Component Analysis through the RunPCA Seurat function. Then, clusters were identified running the FindClusters Seruat function using a resolution value of 0.7. Cluster markers were identified using the FindAllMarkers function, with the following parameters: logfc.threshold = 0.25, min.pct = 0.1, test.use = "roc".

Differential gene expression and Trajectory analyses

Differential gene expression analyses between various single-cell populations were carried out using the glmGamPoi R-package version 1.2.0.[87] using default parameters and building the design matrix between single clusters

Unbiased trajectory analyses were carried out using the monocle3 R-package version 0.2.2. using the PolyKRT cluster as the starting cluster, and removing the use of partitions. Marker-gene directed trajectory analyses were carried out using the Ouija R-package version 0.99.1[51]

Gene expression profile nCounter analysis

The multiplexed NanoString nCounter™Stem Cells panel enriched with a bespoke Stem Plus panel was used as expression assay for profiling of 800 genes environ (NanoString Technologies, Inc., Seattle, WA, USA). The assay was performed according to manufacturer's protocol. In brief, crude cell lysate was used as input material and sorted TEC (CD205^{pos}BCAM^{pos}, CD205^{neg} BCAM^{neg}, EpCAM^{pos}BCAM^{pos} and EpCAM^{pos}BCAM^{neg}) were lysed in RLT lysis buffer (Qiagen) at 2.000 – 10.000 cells/sample. For the multiplex NanoString nCounter™CAR-T Characterization panel, spleens were harvested from Nude-NSG mice implanted with repopulated scaffolds, dissociated to single cells, treated for red blood cell-lysis, and stained for CD45 and CD3. CD3^{pos} cells were sorted at each time point and lysed in RLT lysis buffer (Qiagen) at 1000 - 5910 cells/sample).

Samples were snap-frozen on liquid nitrogen and stored at −80°C. mRNA expression was measured on NanoString nCounter™ MAX system in a final volume of 15 μl by using 2 μl cell lysates mixed with a 3′ biotinylated Capture Probe/Capture probe^{+} and a 5′ Reporter Probe/Reporter probe^{+} tagged with a fluorescent barcode. Probes and target transcripts were hybridized overnight at 65°C for 22 hours following manufacturer recommendations.

Data were collected on an nCounter digital analyzer (NanoString™) and imported into nSolver Analysis Software v4.0 (www.nanostring.com) for data quality check, background thresholding and normalization. The quality of the run for each sample was confirmed by the quality control that considered the 6 spiked-in RNA Positive Control and the 8 Negative controls present in the panel, the FOV (fields of view per sample) counted and the binding density.

Gene expression data were normalized in two steps: (a) by using all the 12 housekeeping genes present in the panels and (b) by adjusting the number of cells/sample to 2000 cells total for each sample.

Background level was determined by mean counts of 8 negative control probes plus two standard deviations. Samples that contain less than 50% of probes above background, or that have imaging or positive control linearity flags, were excluded from further analysis. Probes that have raw counts below background in all samples were excluded from differential expression analysis to avoid false positive results.

For the differential gene expression analysis, we followed the procedure lined out previously.[93] NanoString count matrices were normalized using the RUVSeq R-package and differential gene expression was performed using the R-package DESeq2.

Histology

Human thymic samples were fixed (for 2 hrs to overnight) in 4% PFA and processed for either cryo- or paraffin-embedding. For cryo-embedding, fixed tissue was equilibrated in sucrose 25% and embedded in O.C.T. compound (VWR). Cryosections (thickness, 7 μm) were cut on a Leica Cryostat 3050. For paraffin-embedding, a Leica PelorisII tissue processor and Sakura Tissue-Tech embedding station were used. Paraffin section (thickness, 3-5 μm) were produced using ThermoFisher rotary microtome.

Cryo- or Paraffin sections were stained with haematoxylin-eosin using an automatic station (Tissue-Tek Prisma) to verify histology of each tissue and subsequently used for immunohistochemistry analysis.

Immunostaining

OCT embedded tissue sections or coverslips fixed in 4% PFA were directly blocked and permeabilised simultaneously using a solution of 5% Normal Donkey Serum (NDS, Jackson Immuno Research) in PBS, containing 0.5% of TritonX (TritonTMX-100, SIGMA-ALDRICH).

Paraffin-embedded samples underwent heat inactivated antigen retrieval process in Cytrate Buffer (Sigma-Aldrich) pH 6.0 prior to blocking.

Tissue sections/coverslips were incubated with primary antibodies 5% NDS, 0.01% TritonTMX solution overnight at 4 °C. Secondary antibodies were incubated at room temperature (RT) for 45 minutes. Nuclei were counterstained with Hoechst 33432 (10^{-6} M) or DAPI present in the Fluoroshield™ Mounting Medium (Abcam).

List of primary and secondary antibodies used are listed in A complete list of antibodies used for FACS staining is available in Table S2.

3D Immunostaining of human thymic tissue

Fresh thymic tissue was washed with PBS twice, and subsequently embedded into 8% Ultrapure LMP Agarose #16520-050) and 300μM sections cut with Vibratome (Leica). Excess of agarose was removed, and sections were blocked in 5% Normal Donkey Serum (NDS, Jackson Immuno Research) in PBS, containing 0.5% of TritonX (TritonTMX-100, SIGMA-ALDRICH). Tissues sections were stained in a 12wells plate (Corning) with primary antibodies in 5% NDS, 0.01% TritonTMX solution at RT for 48h. After 3 washes in PBS for 2h each, they were incubated with secondary antibodies in 5% NDS, 0.01% TritonTMX solution ON at RT, the day after stained with DAPI for 1h at RT and finally washed in PBS twice 30min each. Tissue dehydration was performed with ascending methanol concentrations 20min each at RT (1-25-50-75-100% in PBS). Tissue clearing was obtained by incubating with 50%BABB(-Sigma-Aldrich) in methanol for 1h RT and subsequently in 100% BABB only for 1h RT. Tissue was kept in fresh BABB 100% at 4C and imaged within 16h at Leica SP8 Microscope (10X objective), after being mounted on a metallic chamber (#A7816, Thermofisher).

Spatial profiling with GeoMx

The NanoString GeoMx Digital Spatial Profiler (DSP) enables spatially resolved RNA gene expression. Detailed methods have previously been described.[52] 5-μm-thick serial sections derived from four FFPE thymic blocks (4 months to 1.5 years old) were cut and mounted on a positively charged histology slide for use in the DSP. After baking at 65°C for 2 hrs for paraffin removal, slides were loaded onto a Leica Bond Rx for rehydration, heat-induced epitope retrieval (HIER2 for 20 minutes at 100°C) and digestion with proteinase K (1.0 μg/ml for 15 minutes at 37°C). The tissue sections were then hybridized with the Human WTA probes overnight. Following 2x 5min stringent washes in 1:1 4x SSC buffer & formamide, the slides were blocked and three antibodies (morphology markers) were used as a means to separate the ROIs into our cell populations of interest (CD3 (Origene cat# UM500048CF, conjugated by TAP with AF 647 using a kit from Thermo (cat# A20186)) and CD45 (Novus Bio Cat# NBP2-34528 – TAP also conjugated CD45 with AF647 as per above) for thymocytes, Fibronectin (FN1, Protein tech, 1:200) and anti-KRT8-18 (Acris/2BeScientific, cat# BP5075 1:200) to select for thymic epithelial cells) and perform segmental analysis. After 1.5 hr incubation with primary antibodies, slides were subsequently incubated with fluorescently conjugated secondary antibodies anti-rabbit AF594 and anti-guinea pig AF488 (all diluted 1:500). Nuclei have been counterstained with 300nM Syto83.

Four types of regions of interest (ROIs) were selected using polygon tool in cortical and medullary areas, respectively: central cortex (CC), Subcapsulae (SCap), FN1 medulla (FNm) and Hassall's Bodies region (HB). Areas have been segmented by selecting KRT8/18 positive and CD45/CD3 negative cells.

Slides were loaded onto a DSP instrument and a programmable digital micromirror device directed UV light to precisely illuminate the ROI and to cleave the photo-cleavable-oligo-labelled primary antibodies in a region-specific manner and then collected and quantified by NGS as described (52). Library preparation was performed according to manufacturer's instruction (Nanostring DSP-Genomics Library Preparation Protocol). Photocleaved oligonucleotides from each ROI were PCR amplified using Illumina's i5/i7 dual-indexing system to preserve ROI identity. PCR products were pooled and purified with two rounds of AMPure XP beads (Beckman Coulter). Bioanalyzer High Sensitivity DNA chip (Agilent technologies) was used to measure library concentration and purity. Paired end (2 × 75 bp reads) sequencing was performed on an Illumina NextSeq instrument. Reads were then aligned to analyte barcodes with Bowtie.

Sequencing quality was inspected for sufficient saturation, ensuring sensitivity of low expressors and data was normalized to the third quartile (Q3) to account for differences in cellularity and ROI Size.

AKOYA Multiplex Imaging analysis

3μm thymus sections were baked for 1hr and run on the Leica Bond Rx platform using Epitope Retrieval 1 for 20 minutes antigen retrieval and stripping steps between each antibody. Antibodies were applied with Opal™ pairings in the following order: BCAM (NBP2-31994) 1:100 with Opal520 1:1000, TP63 (Ab735) 1:100 with Opal690 1:1000 and KRT15 (HPA023910) 1:500 with TSA-DIG 1:100 followed by anti-DIG-Opal780 1:25. Bond Post-Primary and Polymer secondaries were used for antibodies raised in mouse and Vector ImmPRESS™ HRP Horse anti-Rabbit IgG Polymer (MP-7401-50) secondary was used for antibodies raised in rabbit. Slides were scanned using the PhenoImager HT (formerly Vectra Polaris). Spectral unmixing and autofluorescence removal were performed using Phenochart™ and Inform® software.

Cell quantification has been performed in QuPath Version 0.3.2. Using available software tools to annotate images, annotations have been created around 15-20 cortical and medullary regions. TP63 positive epithelial cells have been detected and counted in each area. All epithelial cells have been assessed for the expression of KRT15 and BCAM marker independently, via object classification and triple positive cells for TP63, KRT15 and BCAM and have been counted combining the single classifications in a composite classifier. At least 2000 $TP63^{pos}$ cells per cortical or medullary area were counted for each biological replicate. Triple positive cells have been represented as percentages of total epithelial cells.

Imaging

Phase contrast images of cultivated cells were acquired using an Olympus CK40 inverted microscope and Olympus SC50 camera.

Zeiss LSM880 inverted confocal microscope and Zen Black software was used to acquire immunofluorescence images on tissue thin sections as well as coverslips.

3D-thick stacks were acquired both with Leica SP8 Microscope (10X objective) and Zeiss LSM880 and analysed with Imaris 9.3 software.

Confocal images were processed using Fiji 2.9.0 and Icy Imaging Analysis Software 2.4.3.

QUANTIFICATION AND STATISTICAL ANALYSIS

In all the experiments included in this study, three or more biological replicates were performed; the exact sample size (*n*) for each experimental group/condition, is given as a discrete number and indicated in figure legends.

Statistical analysis of non-sequencing data was performed with the GraphPad Prism software (GraphPad) 9.0. Unless otherwise specified, data are presented as the mean with ± SEM and statistical significance was determined using specified test (figure legends). P values < 0.05 are considered significant.

Developmental Cell, Volume *58*

Supplemental information

Defining the identity and the niches of epithelial stem cells with highly pleiotropic multilineage potency in the human thymus

Roberta Ragazzini, Stefan Boeing, Luca Zanieri, Mary Green, Giuseppe D'Agostino, Kerol Bartolovic, Ana Agua-Doce, Maria Greco, Sara A. Watson, Antoniana Batsivari, Linda Ariza-McNaughton, Asllan Gjinovci, David Scoville, Andy Nam, Adrian C. Hayday, Dominique Bonnet, and Paola Bonfanti

Figure S1

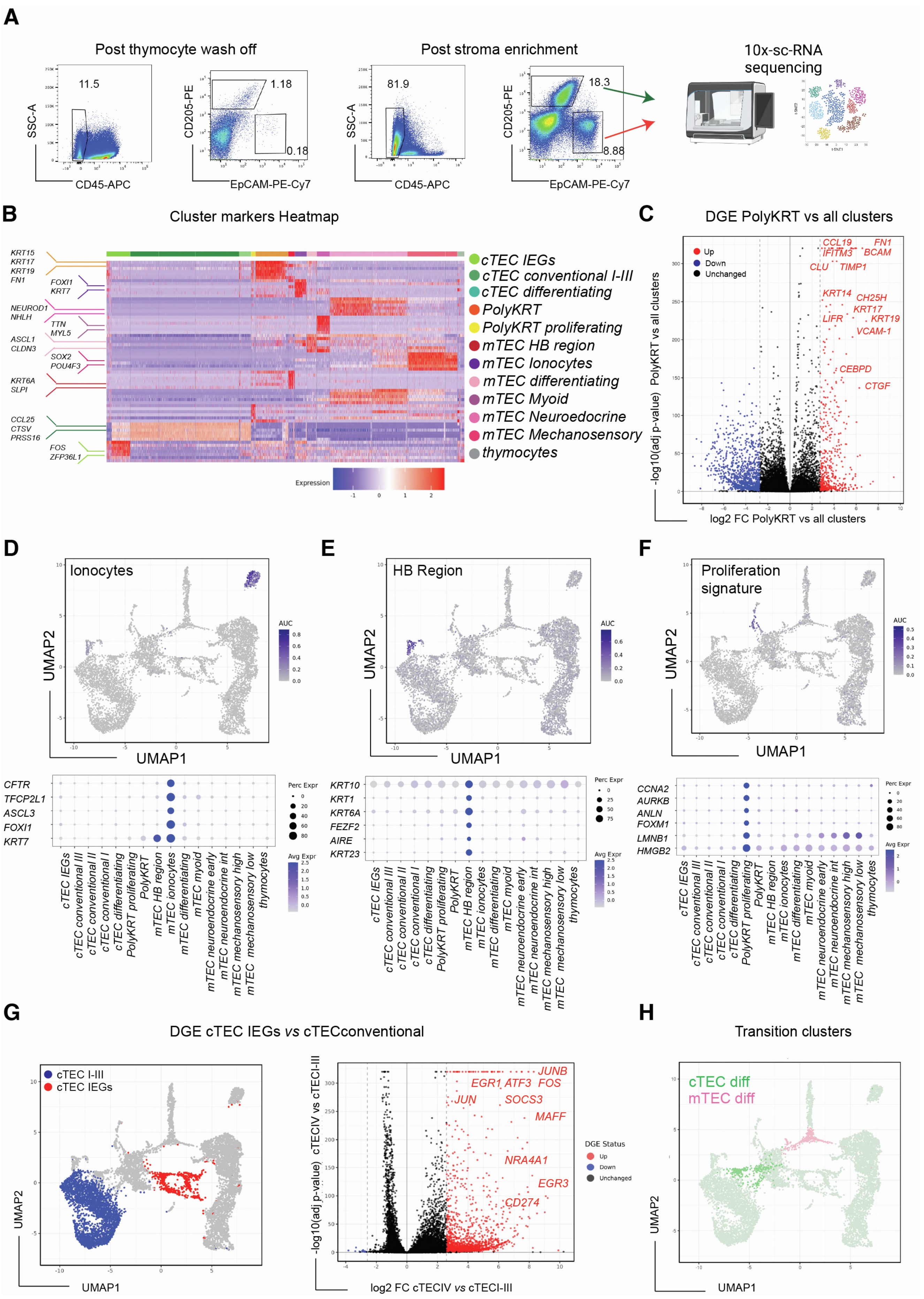

A
Post thymocyte wash off
Post stroma enrichment
10x-sc-RNA sequencing
SSC-A
CD45-APC
CD205-PE
EpCAM-PE-Cy7
11.5
1.18
0.18
81.9
18.3
8.88
B
Cluster markers Heatmap
KRT15
KRT17
KRT19
FN1
FOXI1
KRT7
NEUROD1
NHLH
TTN
MYL5
ASCL1
CLDN3
SOX2
POU4F3
KRT6A
SLPI
CCL25
CTSV
PRSS16
FOS
ZFP36L1
cTEC IEGs
cTEC conventional I-III
cTEC differentiating
PolyKRT
PolyKRT proliferating
mTEC HB region
mTEC Ionocytes
mTEC differentiating
mTEC Myoid
mTEC Neuroedocrine
mTEC Mechanosensory
thymocytes
Expression
C
DGE PolyKRT vs all clusters
Up
Down
Unchanged
CCL19
FN1
IFITM3
BCAM
CLU
TIMP1
KRT14
CH25H
KRT17
LIFR
KRT19
VCAM-1
CEBPD
CTGF
-log10(adj p-value) PolyKRT vs all clusters
log2 FC PolyKRT vs all clusters
D
Ionocytes
E
HB Region
F
Proliferation signature
UMAP2
UMAP1
AUC
CFTR
TFCP2L1
ASCL3
FOXI1
KRT7
KRT10
KRT1
KRT6A
FEZF2
AIRE
KRT23
CCNA2
AURKB
ANLN
FOXM1
LMNB1
HMGB2
Perc Expr
Avg Expr
cTEC IEGs
cTEC conventional III
cTEC conventional II
cTEC conventional I
cTEC differentiating
PolyKRT proliferating
PolyKRT
mTEC HB region
mTEC ionocytes
mTEC differentiating
mTEC myoid
mTEC neuroendocrine early
mTEC neuroendocrine int
mTEC mechanosensory high
mTEC mechanosensory low
thymocytes
G
DGE cTEC IEGs vs cTECconventional
cTEC I-III
cTEC IEGs
JUNB
EGR1 ATF3 FOS
JUN
SOCS3
MAFF
NRA4A1
EGR3
CD274
DGE Status
Up
Down
Unchanged
-log10(adj p-value) cTECIV vs cTECI-III
log2 FC cTECIV vs cTECI-III
H
Transition clusters
cTEC diff
mTEC diff

Figure S1. Human postnatal thymic epithelial cells (TECs) are highly heterogenous including a cluster with stemness traits, related to Figure 1.

(A) Representative FACS plot showing stromal CD45- (left) and cTEC and mTEC populations (right) after several washes to remove thymocytes (left panels) and after stromal enrichment (right panels). Sorted cTECs and mTECs underwent 10X-sequencing. Scheme created with *Biorender.com*.

(B) Comprehensive heatmap showing top upregulated markers for each 16 epithelial clusters identified in the UMAP; cluster names and corresponding colors are indicated on the x axes and on the right side.

(C) Volcano plot analysis of PolyKRT versus all clusters. Each dot represents one gene. A p-value of 0.05 and a fold-change of 2 are indicated by gray lines highlighting the most significantly upregulated (red) and downregulated (blue) genes.

(D-F) UMAP category feature-view and dot plots showing gene category for Ionocytes, mTEC-HB region and proliferation signature genes (*ANLN, AURKB, CCNA2, FOXM1, HMGB2, LMNB1*).

(G) Differential gene expression (DGE) analysis between cTEC-IEGs versus canonical cTEC I-III clusters (left panel). Volcano plot analysis (right panel) of cTEC-IEGs versus canonical cTEC clusters. Each dot represents one gene. A p-value of 0.05 and a fold-change of 2 are indicated by gray lines highlighting the most significantly upregulated (red) and downregulated (blue) genes.

(H) UMAP plot showing cTEC (green) and mTEC (pink) transition (differentiating) clusters.

Figure S2

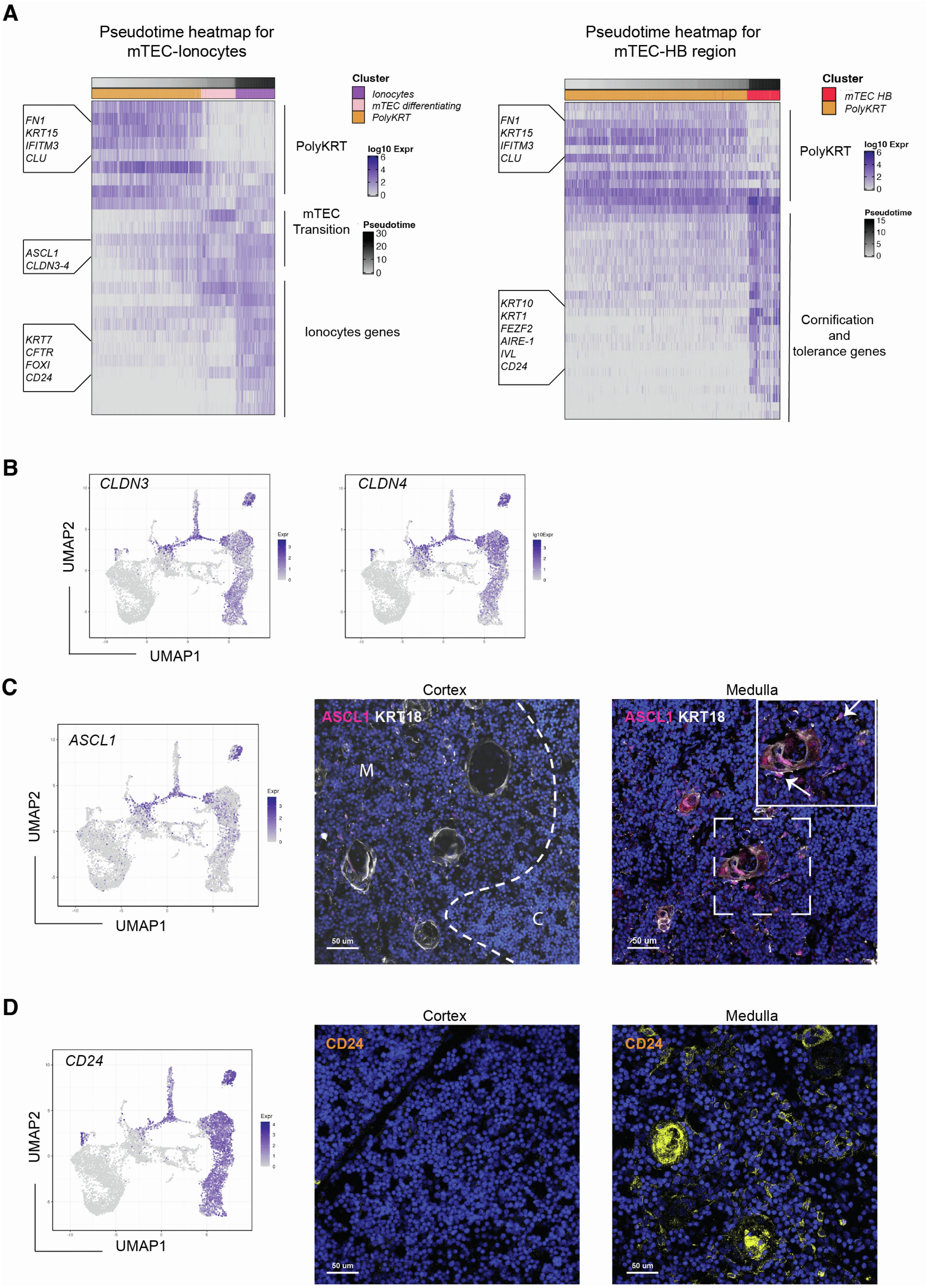

A
Pseudotime heatmap for mTEC-Ionocytes
Cluster
Ionocytes
mTEC differentiating
PolyKRT
FN1
KRT15
IFITM3
CLU
ASCL1
CLDN3-4
KRT7
CFTR
FOXI
CD24
PolyKRT
mTEC Transition
Ionocytes genes
log10 Expr
Pseudotime
Pseudotime heatmap for mTEC-HB region
Cluster
mTEC HB
PolyKRT
KRT10
KRT1
FEZF2
AIRE-1
IVL
CD24
Cornification and tolerance genes
B
CLDN3
CLDN4
UMAP1
UMAP2
C
ASCL1
Cortex
Medulla
ASCL1 KRT18
M
C
50 um
D
CD24
Cortex
Medulla
50 um

Figure S2. Trajectory analysis reveals that specialized mTECs and cTECs differentiate from PolyKRT, related to Figure 2.

(A) Pseudotime Heatmap depicting the most time-variable genes along the single-cell trajectory from PolyKRT to mTEC-differentiating and mTEC-Ionocytes (left) and mTEC-HB region (right). Cluster colors and Pseudotime are indicated at the top of x axes.

(B) UMAP featureview plot of $\log_{10}$ expression of mTEC differentiating marker *CLDN3* (left) and *CLDN4* (right) showing transient expression from PolyKRT to medullary clusters.

(**C**) UMAP featureview plot of $\log_{10}$ expression and IHC (immunohistochemistry) of ASCL1 (magenta) in the human thymus showing co-staining with KRT18 (white). ASCL1 is scattered within medulla and is also found in proximity of HB, while not in cortex. Zoom-in inset in medullary panel highlights nuclear localisation of the transcription factor. Nuclei counterstained with DAPI. Scale bar, 50 µm (n=3, human thymi).

(**D**) UMAP featureview plot of $\log_{10}$ expression and IHC of CD24 (yellow) in medullary areas included HB and not in cortex. Nuclei counterstained with DAPI. Scale bar, 50 µm (n=3, human thymi).

Figure S3

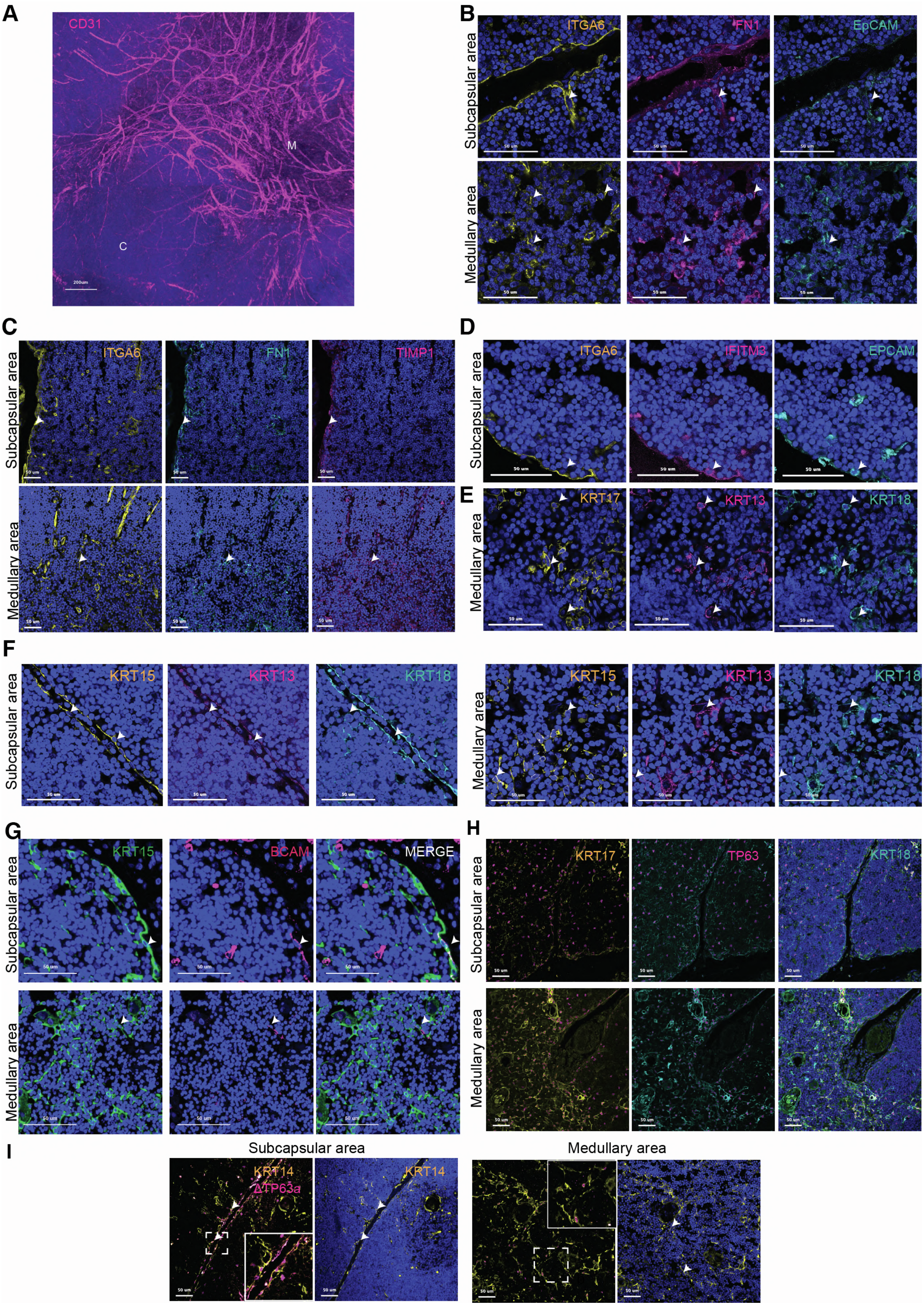
A
CD31
M
C
200um
B
ITGA6
FN1
EpCAM
Subcapsular area
Medullary area
C
ITGA6
FN1
TIMP1
Subcapsular area
Medullary area
D
ITGA6
IFITM3
EPCAM
Subcapsular area
E
KRT17
KRT13
KRT18
Medullary area
F
KRT15
KRT13
KRT18
Subcapsular area
Medullary area
G
KRT15
BCAM
MERGE
Subcapsular area
Medullary area
H
KRT17
TP63
KRT18
Subcapsular area
Medullary area
I
Subcapsular area
Medullary area
KRT14
ΔTP63a
KRT14

Figure S3. PolyKRT cells reside within subcapsular and perivascular niches *in vivo,* related to Figure 3.

(A) 3D reconstruction of thick sections (300µm) of human post-natal thymus stained with endothelial marker CD31 (magenta) in cortical (C) and medullary areas (M). CD31 stains vascular structures present mostly in the medulla. Nuclei counterstained with DAPI. Scale bar, 200 µm.

(B) High magnification images showing immunofluorescence labeling of thymic epithelial cells in human thymi co-stained with anti-ITGA6 (CD49f) antibody (yellow), FN1 (magenta) and EpCAM (cyan). Top panels show co-staining in the subcapsular region; bottom panels show co-staining in medullary area. Arrows (white) highlight individual triple positive cells. Scale bars, 50 µm (n=4, human thymi).

(C) Immunofluorescence labeling of thymic epithelial stem cells in human post-natal thymus identified by co-staining with CD49f/ITGA6 (yellow), FN1 (cyan) and TIMP1 (magenta). Top panels show co-staining in the subcapsular region. Bottom panels show staining in medullary area. Arrows (white) highlight individual triple positive cells (n=3). Scale bars, 50 µm.

(D) Co-staining of CD49f (yellow), EpCAM (cyan) and IFITM3 (magenta) at high magnification in the subcapsular region. Arrows (white) highlight individual triple positive cells. Scale bars, 50 µm.

(E) Immunostaining with anti-KRT17 antibody (yellow), KRT13 (magenta) or KRT8-18 (cyan) at high magnification mark triple positive cells in medulla. Arrows (white) highlight individual cells with stem cell signature (n=4). Scale bars, 50 µm.

(F) Immunofluorescence labeling of human post-natal thymic sections by co-staining of anti-KRT15 antibody (yellow), KRT13 (magenta) or KRT8-18 (cyan) at high magnification. Left panel shows co-staining in subcapsular area and right panel in medulla. Arrows (white) highlight individual cells with triple co-staining (n=4). Scale bars, 50 µm.

(G) Immunofluorescence labeling of thymic epithelial cells co-stained with anti-KRT15 antibody (green) and BCAM (magenta) at high magnification. Top panels show co-staining in the subcapsular region; bottom panels show co-staining in medullary area. Arrows (white) highlight individual double positive cells (n=4). Scale bars, 50 µm.

(H) Immunofluorescence labelling of thymic epithelial cells with anti-KRT17 antibody (yellow), KRT18 (cyan) and TP63 (magenta), while in **(I)** KRT14 (yellow) colocalizes with deltaNTP63alpha variant (magenta) in rare cells indicated by white arrows in both subcapsular and medullary areas (n=3). All nuclei counterstained with DAPI. Scale bar, 50 µm.

Figure S4

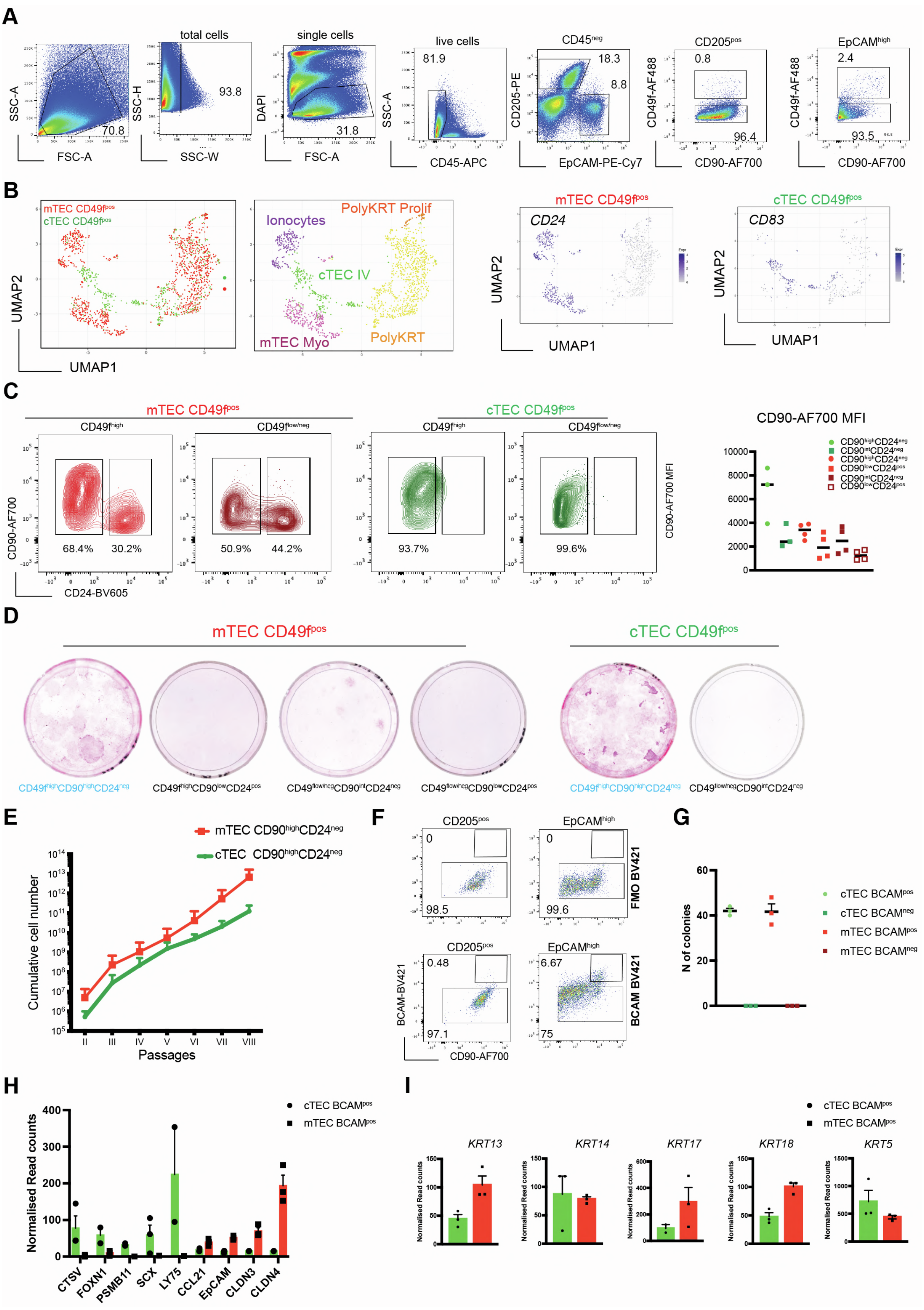
A
total cells
single cells
live cells
CD45neg
CD205pos
EpCAMhigh
SSC-A
FSC-A
SSC-H
SSC-W
DAPI
CD45-APC
CD205-PE
EpCAM-PE-Cy7
CD49f-AF488
CD90-AF700
70.8
93.8
31.8
81.9
18.3
8.8
0.8
96.4
2.4
93.5
B
mTEC CD49fpos
cTEC CD49fpos
Ionocytes
PolyKRT Prolif
cTEC IV
mTEC Myo
PolyKRT
CD24
CD83
UMAP1
UMAP2
C
CD49fhigh
CD49flow/neg
CD90-AF700
CD24-BV605
68.4%
30.2%
50.9%
44.2%
93.7%
99.6%
CD90-AF700 MFI
CD90highCD24neg
CD90intCD24neg
CD90lowCD24pos
D
CD49fhighCD90highCD24neg
CD49fhighCD90lowCD24pos
CD49flow/negCD90intCD24neg
CD49flow/negCD90lowCD24pos
E
mTEC CD90highCD24neg
cTEC CD90highCD24neg
Cumulative cell number
Passages
F
FMO BV421
BCAM BV421
BCAM-BV421
0
98.5
99.6
0.48
97.1
6.67
75
G
N of colonies
cTEC BCAMpos
cTEC BCAMneg
mTEC BCAMpos
mTEC BCAMneg
H
Normalised Read counts
CTSV
FOXN1
PSMB11
SCX
LY75
CCL21
EpCAM
CLDN3
CLDN4
I
KRT13
KRT14
KRT17
KRT18
KRT5

Figure S4. Prospective isolation of mTEC and cTEC PolyKRT, related to Figure 4.

(A) Representative FACS plots showing the gating strategy applied to separate and sort cTEC and mTEC, CD49f^{pos} (7 months old human postnatal thymus). Percentage of cells in each population is shown on the graphs.

(B) Left panels: UMAP dotplot showing scRNAseq of CD49f^{pos} mTEC (red dots) and cTEC (green dots) populations; UMAP dotplot of CD49f^{pos} mTEC and cTEC populations coloured by clusters demonstrating that PolyKRT (yellow and orange clusters) segregate with the CD49f^{pos} sorted fraction. Right panels: UMAP featureview plots of CD49f^{pos} populations show that differentiated clusters are positive for the surface marker CD24 in the medullary CD49f^{pos} (left) and for CD83 in the cortical CD49f^{pos} cells (right), while PolyKRT are negative for either CD24 or CD83.

(C) Representative FACS plots of dissociated and enriched thymic cells for N=10 human postnatal thymi (3 days to 5 years old donors). cTEC and mTEC cells were gated for CD49f^{high} expression and further subdivided and sorted according to CD24 expression. A total of four mTEC populations were isolated: CD49F^{high}CD90^{high}CD24^{neg}, CD49F^{high}CD90^{low}CD24^{pos}, CD49F$^{low/neg}$CD90^{int}CD24^{neg}, CD49F$^{low/neg}$CD90^{low}CD24^{pos}; and two cTEC populations: CD49F^{high}CD90^{high}CD24^{neg} and CD49F$^{low/neg}$CD90^{int}CD24^{neg}. On the right: Median Fluorescence Intensity (MFI) of CD90 was calculated for each of the subpopulations (n=4, human thymi).

(D) Rhodamine-B staining of each mTEC and cTEC sorted population after two passages in culture (n=4, donor-derived cultures).

(**E**) Growth curve of sorted and cultured cTEC (green) and mTEC (red) over serial passaging (x axis, number of weekly passages; y axis, cumulative cell number), mean and error plotted (n = 4, donor derived cultures).

(**F**) Representative FACS plots indicating cTEC and mTEC populations sorted for BCAM expression, (n=13, human thymi. Fluorescence Minus One Controls, FMO, included).

(**G**) Number of colonies counted for BCAM^{pos} and BCAM^{neg} expanded cells at passage II-III: 100-500 cells have been plated at day 0. (n=3, donor-derived cultures).

(H) Gene expression profiles of cortical and medullary markers expressed by BCAM^{pos} TEC fraction via nCounter NanoString Technologies (n=3, human thymi).

(I) Keratin (KRT) genes expression profile of cortical and medullary BCAM^{pos} TEC fractions via nCounter NanoString Technologies (n=3, human thymi). Differences in KRT genes expression is not significant after performing Mann-Whitney test.

Figure S5

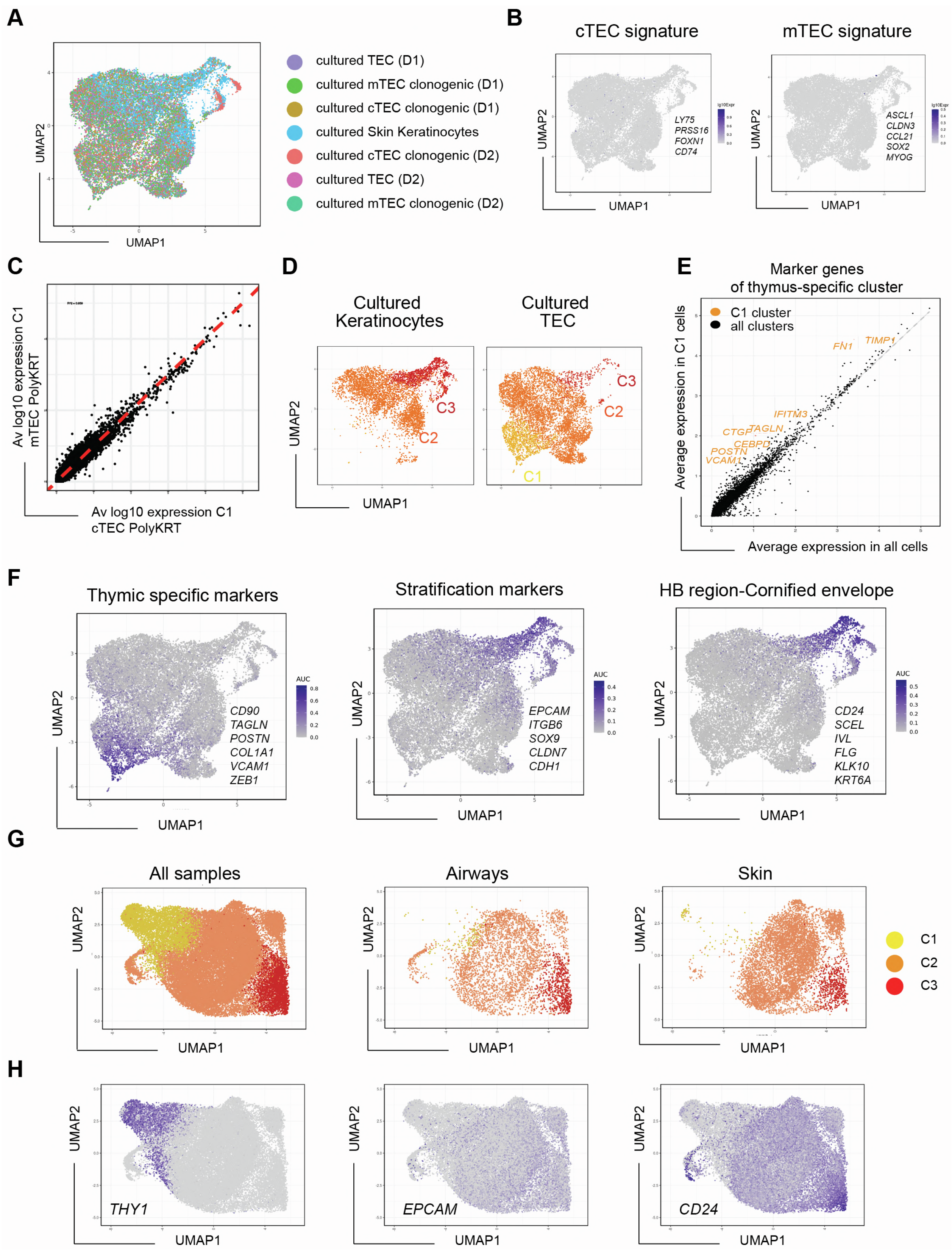
A
UMAP2
UMAP1
cultured TEC (D1)
cultured mTEC clonogenic (D1)
cultured cTEC clonogenic (D1)
cultured Skin Keratinocytes
cultured cTEC clonogenic (D2)
cultured TEC (D2)
cultured mTEC clonogenic (D2)
B
cTEC signature
mTEC signature
LY75
PRSS16
FOXN1
CD74
ASCL1
CLDN3
CCL21
SOX2
MYOG
C
Av log10 expression C1 mTEC PolyKRT
Av log10 expression C1 cTEC PolyKRT
D
Cultured Keratinocytes
Cultured TEC
C1
C2
C3
E
Marker genes of thymus-specific cluster
C1 cluster
all clusters
FN1
TIMP1
IFITM3
CTGF
TAGLN
CEBPD
POSTN
VCAM1
Average expression in C1 cells
Average expression in all cells
F
Thymic specific markers
CD90
TAGLN
POSTN
COL1A1
VCAM1
ZEB1
Stratification markers
EPCAM
ITGB6
SOX9
CLDN7
CDH1
HB region-Cornified envelope
CD24
SCEL
IVL
FLG
KLK10
KRT6A
AUC
G
All samples
Airways
Skin
C1
C2
C3
H
THY1
EPCAM
CD24

Figure S5. Single-cell RNA-seq analysis defines a thymus-specific cell cluster *in vitro,* related to Figure 5.

(A) UMAP plot showing the distribution of each sequenced epithelial cell in culture by similarity. Analysis has been performed on two donor samples (D1 and D2) for each thymic culture type.

(B) UMAP featureview plot of log_{10} expression of specialized cortical (left panel) and medullary (right panel) markers, absent in all thymic cultures.

(C) Comparison of average expression levels between cluster C1 mTEC PolyKRT and C1 cTEC PolyKRT. The red dashed line is the identity line.

(D) UMAP plot visualization of skin keratinocytes and thymic cells colored by cell cluster group per each representative sample. Cluster C1 is absent in skin keratinocytes.

(E) Average expression of C1 genes is plotted against average expression in all the other clusters. Upregulated genes in cluster C1 are displayed in orange.

(F) UMAP category featureview plots showing log_{10} expression of genes belonging to C1 thymic specific cluster: *THY1/CD90, TAGLN, POSTN, COL1A1, VCAM-1, ZEB1* (left panel); genes belonging to C2 stratified cell cluster *EPCAM, ITGB6, SOX9, CLDN7, CDH1*, and C3 cornified/differentiated cluster *CD24, SCEL, IVL, FLG, KLK10, KRT6A*.

(G) UMAP plot visualization of cultured epithelial cells colored by cell cluster group per each representative sample (thymic, skin keratinocytes and airways basal cells).

(H) UMAP plot visualisation (log_{10} expression) of marker genes across clusters confirmed expression of *THY1* in C1, *EPCAM* in C2/C3 and *CD24* in C2/C3 clusters.

Figure S6

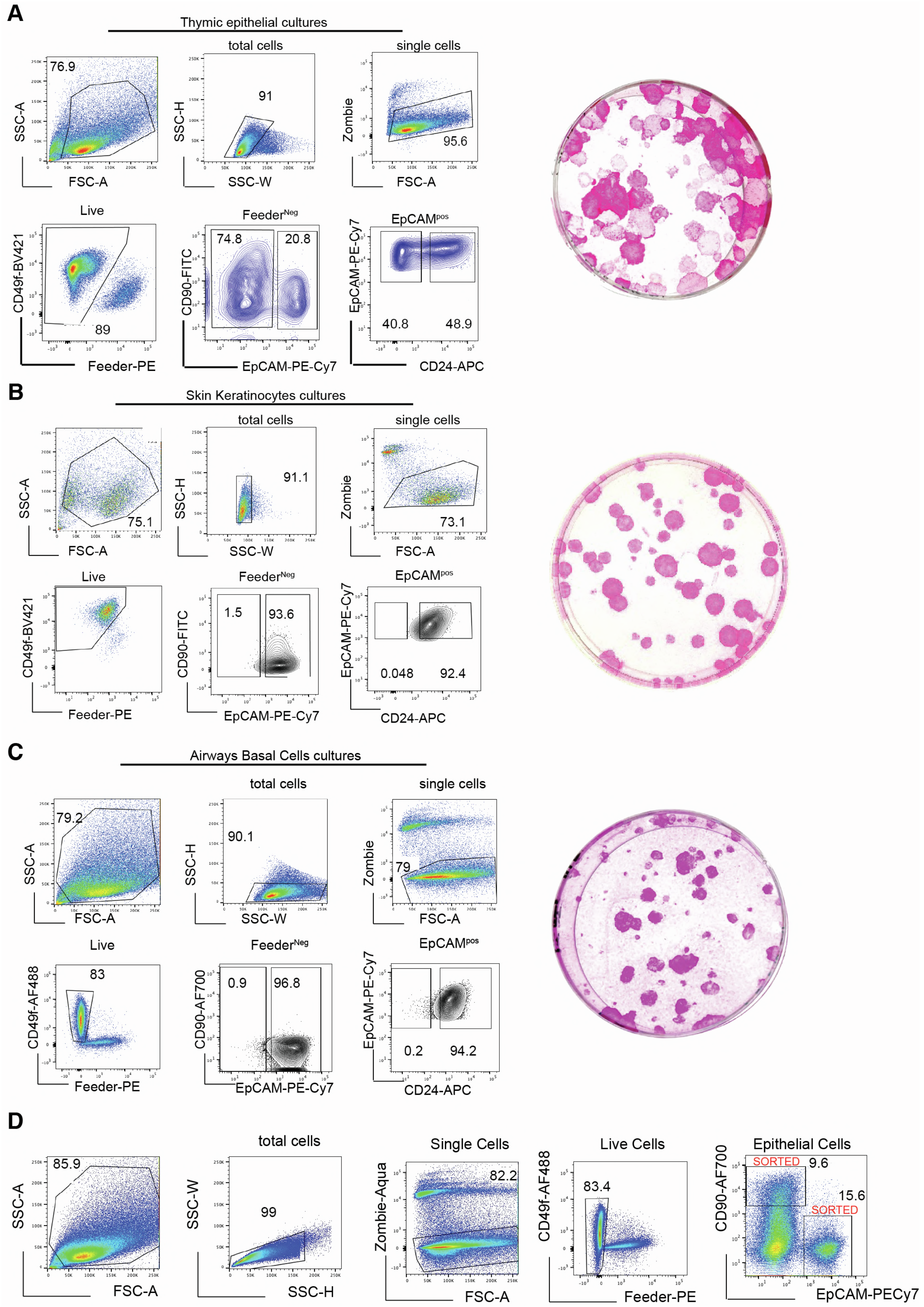
A
Thymic epithelial cultures
total cells
single cells
SSC-A
FSC-A
76.9
SSC-H
SSC-W
91
Zombie
95.6
Live
CD49f-BV421
Feeder-PE
89
FeederNeg
CD90-FITC
EpCAM-PE-Cy7
74.8
20.8
EpCAMpos
CD24-APC
40.8
48.9
B
Skin Keratinocytes cultures
75.1
91.1
73.1
1.5
93.6
0.048
92.4
C
Airways Basal Cells cultures
79.2
90.1
79
83
CD49f-AF488
CD90-AF700
0.9
96.8
0.2
94.2
D
85.9
SSC-W
SSC-H
99
Single Cells
Zombie-Aqua
82.2
Live Cells
83.4
Epithelial Cells
SORTED
9.6
15.6
EpCAM-PECy7

Figure S6. FACS analysis of epithelial SC *in vitro*, related to Figure 5.

(A) Representative FACS analysis of thymic SC showing gating strategy for live cells analysis and CD49f, CD90 (THY1), EpCAM, CD24 distribution (n=8, donor-derived cultures). Right panel: rhodamine-B staining of clonogenic TEC: 500 cells were seeded in a dish for colony-forming efficiency (CFE) assay; the dish was fixed and stained with rhodamine-B after 12 days of culture. Cells gave rise to colonies of variable sizes that stained either strongly or dim with rhodamine-B (n=8, donor-derived cultures).

(B) Representative FACS analysis of expanding cultivated skin keratinocytes reveals a homogeneous population expressing CD49f, EpCAM, CD24 and negative for CD90. Right panel: rhodamine-B staining of skin keratinocytes, performed in the same conditions as per described in (A), showed strong rhodamine-B staining (n=3).

(C) Representative FACS analysis of expanding cultivated airways basal cells shows also a homogeneous population expressing CD49f, EpCAM, CD24 and negative for CD90. Right panel: rhodamine-B staining of airways basal cells, performed in the same conditions as described in (A), showed strong rhodamine B staining (n=3).

(D) Representative FACS analysis of gating strategy for thymic SC sorted cultures (n=8, donor-derived cultures).

Figure S7

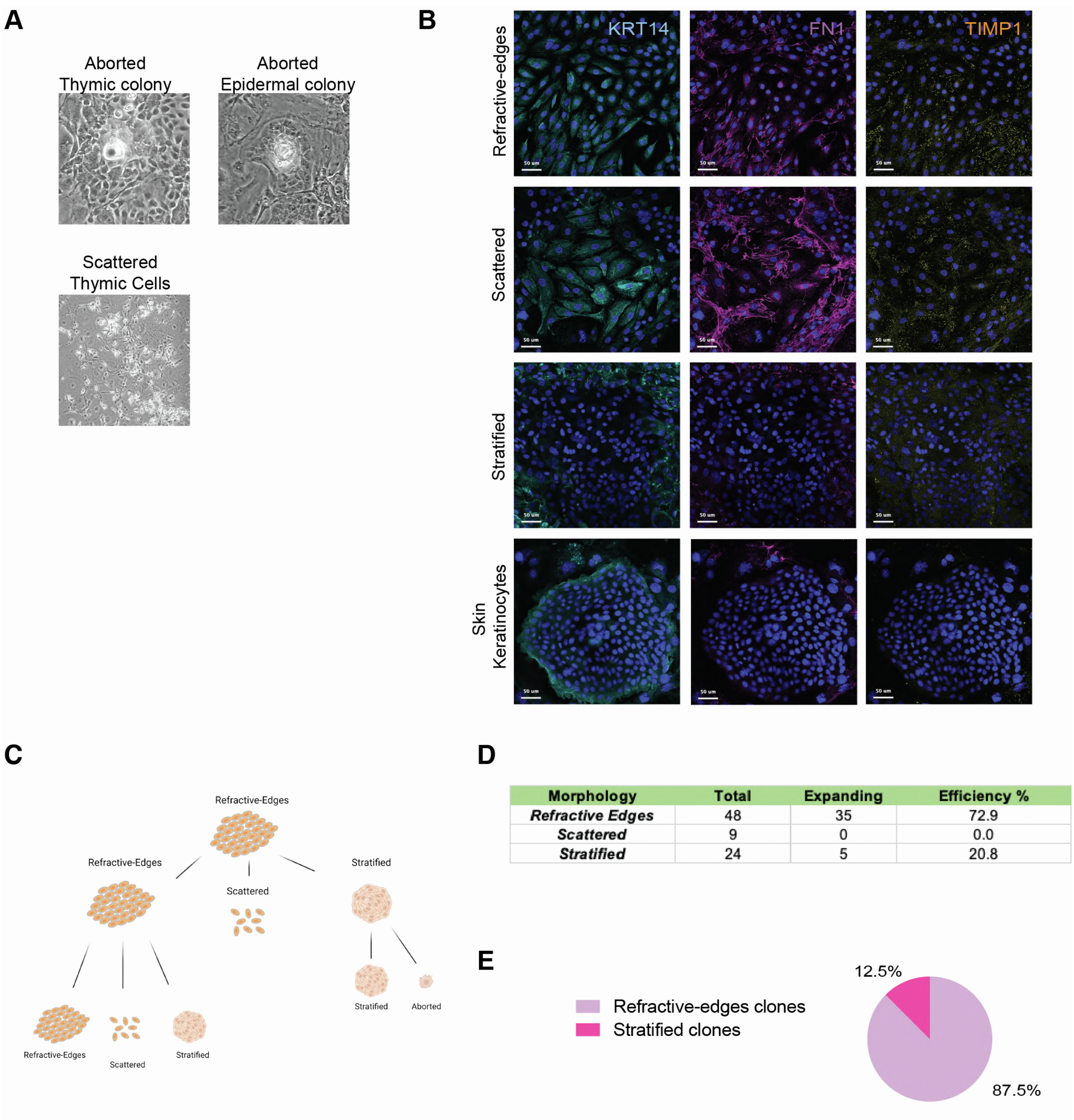

Morphology	Total	Expanding	Efficiency %
Refractive Edges	48	35	72.9
Scattered	9	0	0.0
Stratified	24	5	20.8

Figure S7. Single-cell cloning of thymic SCs, related to Figure 6.

(A) Phase contrast images of individual aborted thymic cells, keratinocyte colonies and thymic scattered cells, respectively.

(B) Immunofluorescence staining of cultivated TECs showing KRT14 (cyan), FN1 (magenta) and TIMP1 (metalloprotease inhibitor protein, in yellow) expression in refractive-edges and scattered, while FN1 and TIMP1 were not detected in stratified and keratinocyte colonies. Nuclei counterstained with DAPI. n=3, donor-derived cultures. Scale bars, 50 µm.

(C) Schematic representing the hierarchical relationship of thymic colonies created with *Biorender.com*.

(D) Table summarizing total number of single-cell clones that were amplified and relative efficiency of expansion per morphology type. Results include four independent cloning experiments (n=4, donor-derived cultures).

(E) Pie chart representing the percentage of expanding clones classified per morphology: light pink corresponds to refractive-edges colonies (87.5%), while purple to stratified clones (12.5%) in n=4 donor-derived cultures.

Figure S8

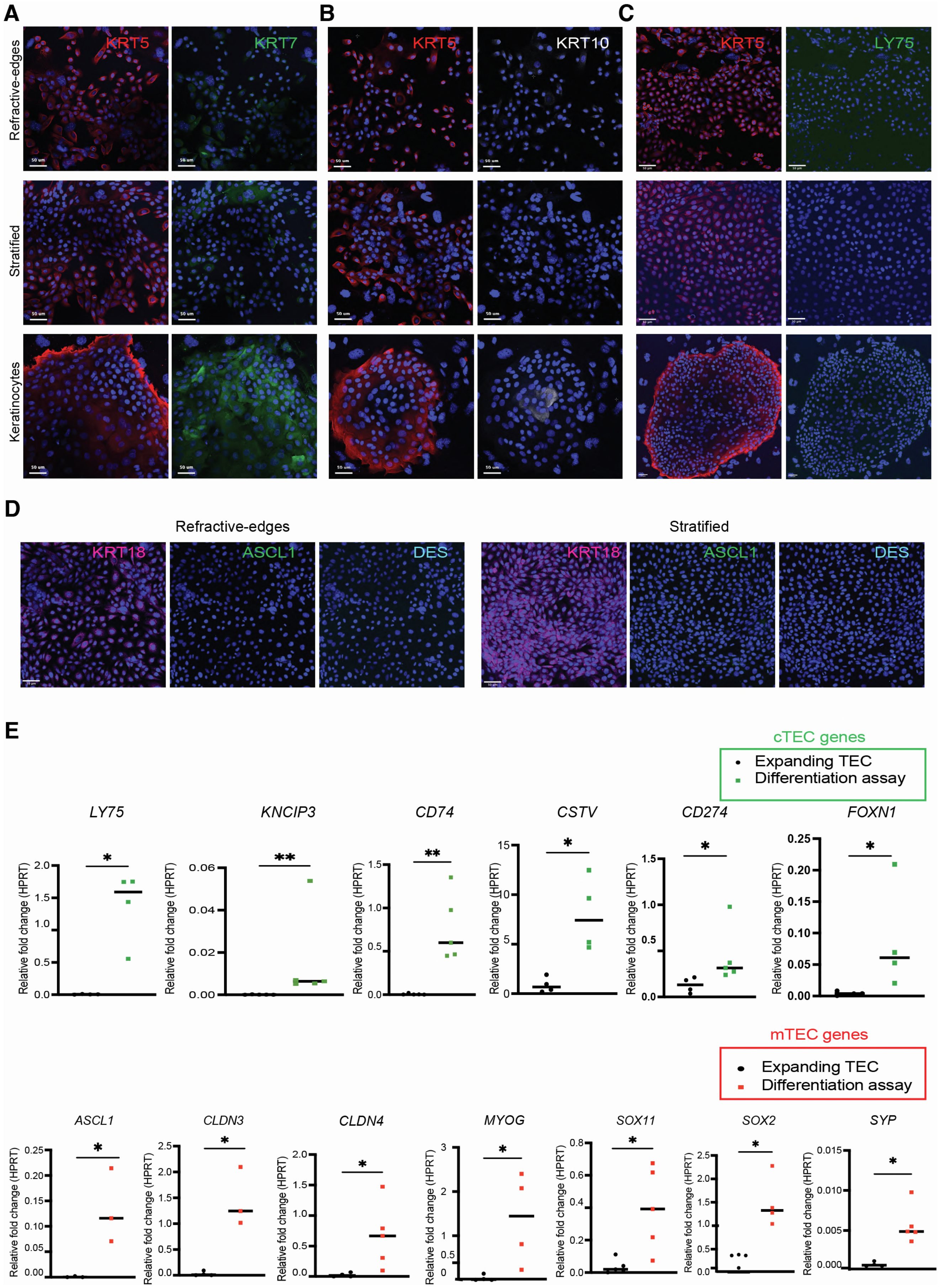

A
B
C
Refractive-edges
Stratified
Keratinocytes
KRT5
KRT7
KRT5
KRT10
KRT5
LY75
D
Refractive-edges
Stratified
KRT18
ASCL1
DES
KRT18
ASCL1
DES
E
cTEC genes
Expanding TEC
Differentiation assay
LY75
KNCIP3
CD74
CSTV
CD274
FOXN1
Relative fold change (HPRT)
mTEC genes
Expanding TEC
Differentiation assay
ASCL1
CLDN3
CLDN4
MYOG
SOX11
SOX2
SYP
Relative fold change (HPRT)

Figure S8. Thymic SCs differentiate into both cortical and medullary fates *in vitro,* related to Figure 7.

(A) Immunofluorescence staining of cultivated thymi and keratinocytes showing expression of KRT5 (red) and KRT7 (green). Thymic SC do not express KRT7 in expansion, while they express KRT5**.** Scale bars, 50 µm.

(B) Immunofluorescence staining of cultivated thymic SC and keratinocytes showing expression of KRT5 (red) and KRT10 (white). Thymic SC do not express KRT10 in expansion, while they express KRT5. Scale bars, 50 µm.

(C) Immunofluorescence staining of cultivated TEC and keratinocytes showing expression of KRT5 (red) and LY75 (green). Neither TECs nor skin keratinocytes express LY75 in expansion. Scale bars, 50 µm.

(D) Immunofluorescence staining of cultivated thymic cells showing expression of KRT18 (magenta), ASCL1 (green) and DES (cyan). Thymic SC do not express either ASCL1 and DES proteins in expansion. Nuclei counterstained with DAPI. n=3, Scale bar, 50 µm**.**

(E) RT-qPCR analysis of expanded thymic cells in 2D expansion (black dots) versus differentiation showing upregulation of cortical genes (*LY75, KCNIP3, CD74, CSTV, CD274, FOXN1*) in green (top panel) and medullary genes (*ASCL1, CLDN3, CLDN4, MYOG, ,SOX11, SOX2, SYP*) in red (bottom panel). Relative gene expression to *HPRT* housekeeping is shown in y axis; significance: Mann-Whitney test, non-parametric; *p<0.05; **p<0.01, n=5, independent clones; mean ±S.E.M.

Figure S9

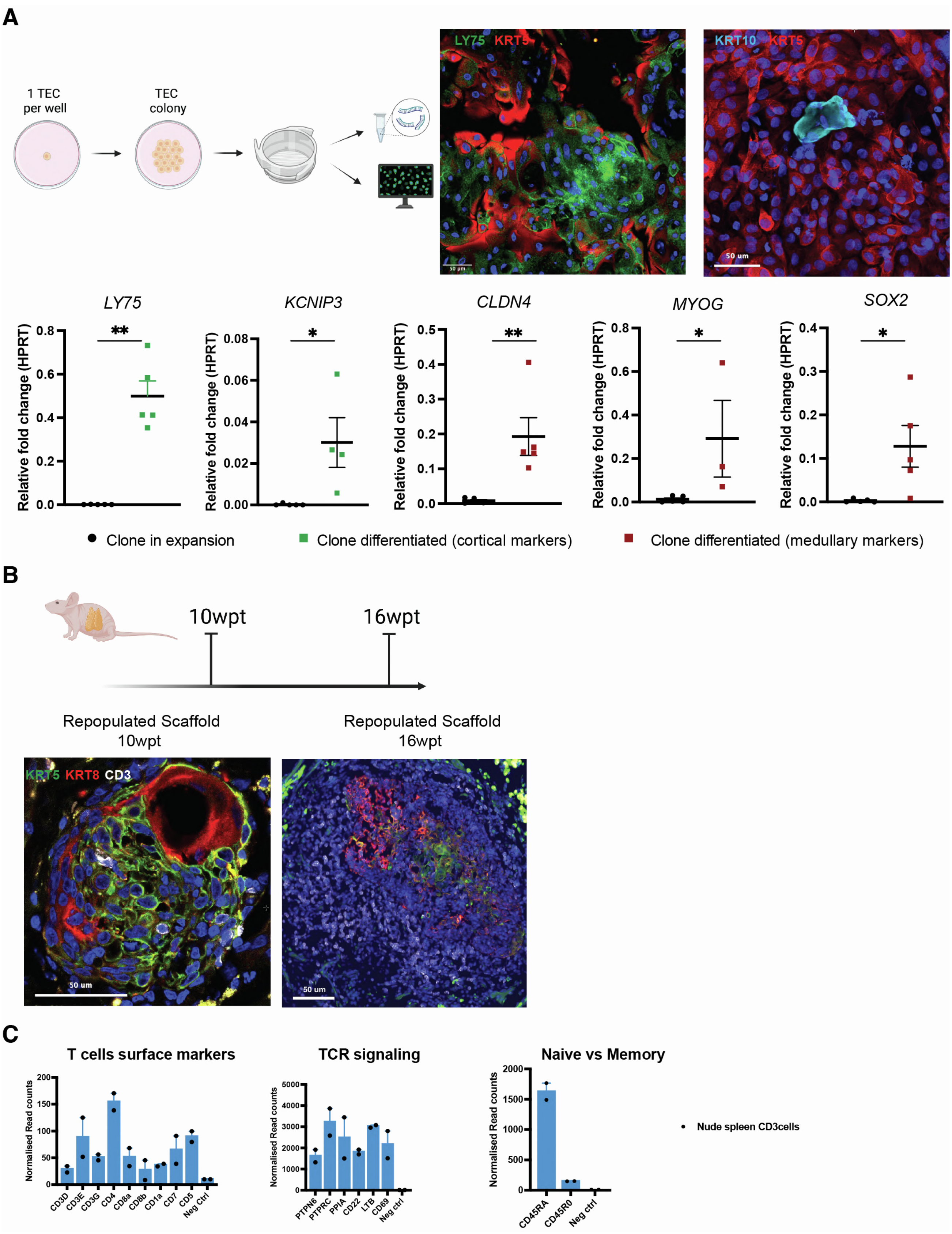
A
1 TEC per well
TEC colony
LY75 KRT5
50 µm
KRT10 KRT5
50 um
LY75
KCNIP3
CLDN4
MYOG
SOX2
Relative fold change (HPRT)
**
*
**
*
*
Clone in expansion
Clone differentiated (cortical markers)
Clone differentiated (medullary markers)
B
10wpt
16wpt
Repopulated Scaffold 10wpt
Repopulated Scaffold 16wpt
KRT5 KRT8 CD3
50 um
50 um
C
T cells surface markers
TCR signaling
Naive vs Memory
Normalised Read counts
CD3D
CD3E
CD3G
CD4
CD8a
CD8b
CD1a
CD7
CD5
Neg Ctrl
PTPN6
PTPRC
PPIA
CD22
LTB
CD69
Neg ctrl
CD45RA
CD45R0
Neg ctrl
Nude spleen CD3cells

Figure S9. Thymic SCs differentiate into both cortical and medullary fates *in vitro* and *in vitro,* related to Figure 7.

(A) Schematic workflow for TEC differentiation assay from a single TEC clone. Image created with *Biorender.com*. TEC clone, differentiated after expansion, shows cells positive for KRT5 (red), cortical cells (LY75-positive, green) on the left and areas with Hassall's Bodies (HB) structures positive for KRT10 (cyan) on the right. Nuclei counterstained with DAPI. Scale bar, 50 µm (representative image, n=5, independent clones). Bottom panels: RT-qPCR analysis of cultivated TEC clones in 2D expansion (black dots) versus TEC clones after differentiation indicated upregulation of cortical genes (*LY75, KCNIP3*) in green dots and medullary genes (*CLDN4, MYOG, SOX2*) in red squares. Relative gene expression to *HPRT* housekeeping is shown in y axis; significance: Mann-Whitney test, non-parametric; *$p<0.05$; **$p<0.01$, n=5, independent clones; mean ±S.E.M.

(B) Schematic illustrating timeline of *in vivo* transplantation of scaffolds repopulated with TEC and TIC (thymic interstitial cells) into humanized nude athymic mice (top panel). Immunofluorescent images of thymic scaffold grafts sections (7µm) at week 10 and 16 post transplantation stained for human KRT5 (green), human KRT8-18 (red) and human CD3 (gray). CD3 cells progressively colonize (10 weeks) and increase their presence within repopulated human epithelial areas in the scaffolds by 16 weeks. Nuclei counterstained with DAPI. Scale bars, 50 µm (n=8, scaffolds per time point).

(C) Single gene expression profiles of T cell surface markers (left), TCR signaling components (centre) and naïve versus memory markers (right) expressed in $CD3^+$ cells sorted from spleen samples of grafted NSG-Nude mice and analyzed by nCounter NanoString (n=4 implanted scaffolds grouped for each time point).

Table S1. Pseudotime of thymic SC *in vitro*, related to Figure 5
Pseudotime Heatmap (Monocle) depicting the most time-variable genes along the single-cell trajectory from cluster C1 to C2 and C3. Single genes along the trajectory are indicated on the right side of the graph. Log_{10} expression, Pseudotime value and clusters are indicated in the legends on the right side. Cluster colors and Pseudotime are indicated at the top of x axes (N=8, thymus-derived cultures).

Table S2. Antibody List, related to STAR Methods

Table S3. qPCR probes, related to STAR Methods

Table S1. Pseudotime of thymic SC *in vitro*, related to Figure 5

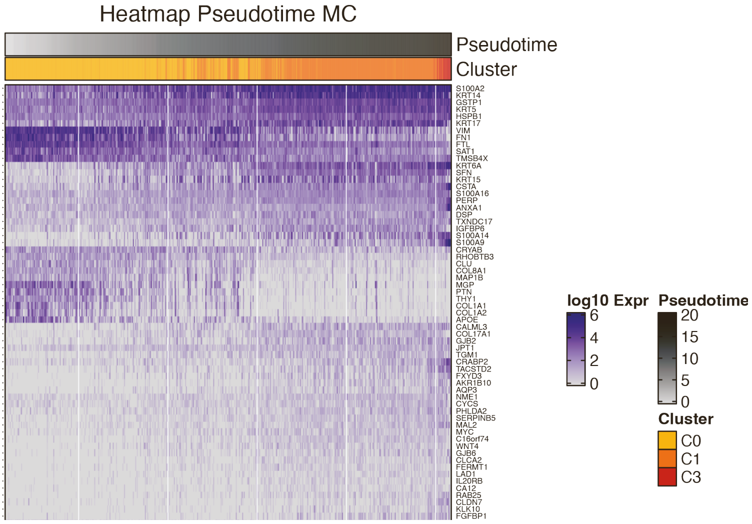

Table S1 related to STAR METHODS

FACS antibodies list

Antigen	Fluorocrome	Dilution	Company	Cat.No.
BCAM	BV421	1:100	BD Bioscience	748007
CD24	APC	1:100	BioLegend	311117
CD24	BV605	1:100	BioLegend	311124
CD205	PE	1:400	BioLegend	342203
CD235ab (glycophorin A and B)	Biotin	1:100	BioLegend	306618
CD45	Biotin	1:100	BioLegend	103104
CD45	APC	1:200	BioLegend	304011
CD49f	AF488	1:200	BioLegend	313608
CD49f	BV421	1:200	BioLegend	313624
CD90	AF700	1:200	Biolegend	328119
CD90	FITC	1:200	Biolegend	328108
EpCAM (CD326)	PE-Cy7	1:100	BioLegend	324222
Feeder	PE	1:100	Miltenyi	130120166
Zombie Aqua™ Fixable Viability Kit		1:200	Biolegend	423102
DAPI		1:300	Sigma	D9542

Primary and Secondary Antibodies list

Antigen	Host	Dilution	Company	Cat.No.
CD49f- AF488 conjugated	Rat	1:100	Biolegend	313608
Ly75 (CD205)	Rabbit	1:100	Abcam	AB124897
CK5	Mouse	1:100	Abcam	AB17130
CK7	Rabbit	1:100	Sigma Aldrich	HPA007272-100UL
CK10	Mouse	1:200	Santa Cruz	SC-53252
CK13	Mouse	1:100	Abcam	AB16112
CK14	Chicken	1:200	Biolegend	905301
CK15	Rabbit	1:100	Sigma Aldrich	HPA023910
CK17	Rabbit	1:100	Sigma Aldrich	HPA000453
CK8	Mouse	1:50	Abcam	AB9023
CK8/18	Guinea pig	1:100	Acris/2BeScientific	BP5075
EpCAM- efluor660 cojugated	Mouse	1:100	eBioscience	50-9326-42
p63	Mouse	1:50	Abcam	AB735
IFITM3	Rabbit	1:100	Thermofisher	MA5-32798
FN1	Rabbit	1:300	proteintech	15613-1-AP
THY-1 (CD90)	Mouse	1:100	Biolegend	328108
deltaNTP63alpha	Rabbit	1:100000		kindly donated by Michele De Luca
BCAM	Mouse	1:100	BD Bioscience	748007
BCAM	Rabbit	1:100	Novus Biological	NBP2-31994
CD24-APC conjugated	Mouse	1:100	BioLegend	311117
ASCL1	Rabbit	1:100	Abcam	AB74065
TIMP1	Mouse	1:50	Invitrogen	MA1-773
SOX2	Goat	1:100	R&D Systems	AF2018
CD45	Rabbit	1:200	Abcam	AB40763
CD45	Mouse	1:100	Novus Biological	NBP2-34528
CD3E	Mouse	1:100	Origene	UM500048
CD3 APC conjugated	Mouse	1:100	BioLegend	300411
Desmin	Mouse	1:100	Agilent/DAKO	M0760

Secondary Antibodies

Anti-Guinea Pig AF594	Donkey	1:500	Jackson Immuno	706-165-148
Anti-Mouse AF488	Donkey	1:500	Jackson Immuno	715-545-150
Anti-Mouse AF594	Donkey	1:500	Jackson Immuno	715-165-150
Anti-Mouse AF647	Donkey	1:500	Jackson Immuno	715-605-150
Anti-Rabbit AF488	Donkey	1:500	Jackson Immuno	711-545-152
Anti-Rabbit AF594	Donkey	1:500	Jackson Immuno	711-585-152
Anti-Rabbit AF647	Donkey	1:500	Jackson Immuno	711-605-152
Anti-Rat AF488	Donkey	1:500	Jackson Immuno	712-545-150
Anti-Rat AF647	Donkey	1:500	Jackson Immuno	712-605-150
Anti-Chicken AF488	Donkey	1:500	Jackson Immuno	703-225-155
Anti-Chicken AF594	Donkey	1:500	Jackson Immuno	705-585-155
Anti-Goat AF488	Donkey	1:500	Jackson Immuno	705-545-147
Anti-Goat AF594	Donkey	1:500	Jackson Immuno	705-165-147

Table S2 related to STAR METHODS
qPCR probes list

Gene	Assay name	Probe	Primer 1	Primer 2
HPRT1	Hs.PT.SSv.45621572	5'-/56-FAM/ AGCCT AAGATGAGAGTTCAAGTTGAGTTTGG /36-TAMSp/-3'	5'-TTGTTGT AGGAT ATGCCCTTGA-3'	5'-GCGATGTCAATAGGACTCCAG-3'
LY75	Hs.PT.583240196	5'-/56-FAM/ TGATAGCTATTGCTGTGTAATCAGGGCC /36-TAMSp/-3'	5'-CCGCCCATGAGAACTAAGATAC-3'	5'-GGTTTTGGAAGAGTTGTCTGC-3'
KCNIP3	Hs.PT.58.20750533	5'-/56-FAM/ CCTGGGCGCTGAAGAAAACTGTTG /36-TAMSp/-3'	5'-TGTGAAGTGGGGAGGCT-3'	5'-ACTTGATACCCTCCTTCTTGC-3'
CD74	Hs.PT.58.4651852	5'-/56-FAM/ CCAGCGCGACCTT ATCTCCAACA /36-TAMSp/-3	5'-GGAAGATCAGAAGCCAGTCAT-3'	5'-CAGAGTCACCAGGATGGAAA-3'
CTSV	Hs.PT.58.370453	5'-/56-FAM/ AGCCAACCACCAGAACACCATGAT /36-T AMSp/-3'	5'-CCATTCGTCCTTCCAGTTCTAC-3'	5'-TTGACGAGCCAATACTTGCT-3'
CLDN4	Hs.PT.58.1326185.g	5'-/56-FAM/ ACCAGTTCCTTCCAGCCGACAG /36-TAMSp/-3'	5'-CCATATAACTGCTCAACCTGTCC-3'	5'-AGATAAAGCCAGTCCTGATGC-3'
MYOG	Hs.PT.58.38897870	5'-/56-FAM/ ATGCCCGGCTTGGAAGACAATCT /36-TAMSp/-3'	5'-GACAGCATCACAGTGGAAGA-3'	5'-AGAAGTAGTGGCATCTGTGG-3'
SOX2	Hs.PT.58.237897.g	5'-/56-FAM/ CACCTACAGCATGTCCTACTCGCA /36-TAMSp/-3'	5'-GTACAACTCCATGACCAGCTC-3'	5'-CTTGACCACCGAACCCAT-3'
SYP	Hs.PT.58.27207712	5'-/56-FAM/ TGTACTTTGATGCACCCACCTGCC /36-TAMSp/-3'	5'-AGTCCCCAACTAAGAAGACCT-3'	5'-ACAAGACCGAGAGTGACCT-3'
ASCL1	Hs.PT.56a.23464.gs	5'-/56-FAM/ CGATCACCCTGCTTCCAAAGTCCA /36-TAMSp/-3'	5'-AGCTTCTCGACTTCACCAAC-3'	5'-CAACGCCACTGACAAGAAAG-3'
CLDN3	Hs.PT.58.45414673.g	5'-/56-FAM/ TCCACAGGCCCTCCCAGATG /36-TAMSp/-3'	5'-CAACATCATCACGTCGCAGA-3'	5'-GAGTCGTACACCTTGCACTG-3'
SOX11	Hs.PT.58.2452961 5.g	5'-/56-FAM/ ACACAACAGCCTCACCACCGAA /36-TAMSp/-3'	5'-ACTGAT AGAAACTCGCATCGC-3'	5'-GAATTCTCTCCTGCCACCTC-3'
CD274	Hs.PT.58.20308441	5'-/56-FAM/ CCCAAGGACCTATATGTGGTAGAGTATGGT /36-TAMSp/-3'	5'-CTACTGGCATTTGCTGAACG-3'	5'-CCAATAGACAATTAGTGCAGCCA-3'
FOXN1	Hs.PT.58.40389553	5'-/56-FAM/ TCTCGCTGACGGGAAAGGCTC /36-TAMSp/-3'	5'-CCCAAAACCCATCTATTCCTACA-3'	5'-GTCTTGAAGTAAGGAAAGTGCTC-3'
